# Singlet spin order in spin pairs coupled via non-bonded interactions

**DOI:** 10.3389/fchem.2024.1511720

**Published:** 2025-01-30

**Authors:** Giuseppe Pileio, Dolnapa Yamano, Craig D. Eccles, Graham J. Tizzard, Sam Thompson

**Affiliations:** ^1^ School of Chemistry, University of Southampton, Southampton, United Kingdom; ^2^ Magritek GmbH, Aachen, Germany; ^3^ UK National Crystallography Service, School of Chemistry, University of Southampton, Southampton, United Kingdom

**Keywords:** long-lived spin states, nuclear singlet spin order, through-space scalar coupling, NMR, fluorine NMR

## Abstract

Fluorine spin pairs that are constrained in spatial proximity show large scalar spin-spin couplings, despite the atoms being separated by several bonds. This is due to a non-bonded atomic interaction related to partial overlapping of fluorine p-orbitals. In this paper we exploit this phenomenon to create long-lived singlet spin order on the fluorine spin pair. This form of order, which, in this example molecule, is more than an order of magnitude longer than longitudinal order, has the potential to be useful in magnetic resonance imaging and molecular tracing experiments, because of the lack of endogenous fluorine in the human body and the high sensitivity achievable in ^19^F NMR.

## 1 Introduction

Molecular systems containing two fluorine atoms that are separated by several chemical bonds but close in space, exhibit significant indirect scalar spin-spin coupling constants. In a first observation in 1956, Saika and Gutowsky were surprised by the large ^5^ $J_{FF}=16$ Hz measured in ${\left({\text{CF}}_{3}\right)}_{2}{\text{NCF}}_{2}{\text{CF}}_{3}$ , where the three-bond coupling was ^3^ $J_{FF}\approx 1$ Hz (Saika and Gutowsky, 1956) and it was common to expect a monotonic decrease of the scalar coupling constant as the number of chemical bonds between the coupled atoms increases. Since then the phenomenon was observed in many other systems including, saturated organic compounds (Petrakis and Sederholm, 1961), 1-substituted 4,5-difluoro-8-methylphenanthrenes (Servis and Fang, 1968), 4-substituted 1,8-difluoronaphthalenes (Mallory et al., 1974), benzophenanthrenes (Mallory et al., 1985), fluoroallyl cations (Bakhmutov et al., 1985), and difluorometacyclophanes (Ernst et al., 1994; Ernst and Ibrom, 1995). The anomalous size of this coupling interaction in these systems was later rationalised in terms of molecular orbital theory (Mallory et al., 1996) and correlated to the spatial distance between the two fluorine nuclei (Mallory et al., 2000), hence the label “through-space”, which is clearly improper in the NMR literature since this same label is commonly used to denote the dipole-dipole interaction between spins that has a truly through-space nature. In such difluoro-substituted systems, geometrical constraints give rise to a certain degree of overlap between the p-orbitals (lone pairs) of the two fluorine atoms and this produces electron-filled molecular orbitals with a weakly bonding ( $\sigma_{FF}$ ) and a weakly anti-bonding $\sigma_{FF}^{*}$ character. Despite this molecular orbital configuration not leading to a net bond between the two fluorine nuclei, it results in a very effective scalar coupling interaction between the two nuclear spins, effectively mediated by the four electrons in those weak molecular orbitals. Since the p-orbital overlap is distance dependant, the magnitude of the observed scalar coupling rapidly decays with increasing distance between the fluorine atoms. It is also worth remembering that there is indeed a through bond contribution to those couplings but this is typically smaller than the “through-space” one. ^19^F-^19^F scalar coupling values of between 60–80 Hz have been measured in substituted 1,8-difluoronaphthalene derivatives (Mallory et al., 1974) and scalar coupling as large as 160–180 Hz were found in substituted 4,5-difluorophenanthrene derivatives (Servis and Fang, 1968).

Systems of two scalar-coupled spin-1/2 nuclei, can be prepared to support singlet spin order, a particular type of nuclear spin order that has been shown to decay much slower than longitudinal and transverse magnetization, the latter two forms of spin order being used in all magnetic resonance experiments. Such a slower decay has been exploited in many applications including storage of hyperpolarization (Vasos et al., 2009; Pileio et al., 2013; Kiryutin et al., 2019), investigation of weak ligand-protein binding (Salvi et al., 2012), measurements of slow chemical exchange (Sarkar et al., 2007), small diffusion coefficients (Cavadini et al., 2005), diffusion and tortuosity in porous media (Dumez et al., 2014; Pileio et al., 2015; Pileio and Ostrowska, 2017; Tourell et al., 2018; Melchiorre et al., 2023). Most of these applications were so far based on singlet order created either in ^1^H spin-1/2 pairs (due to the molecule of interest or to maximize sensitivity) or in ^13^C- or ^15^N-doubly enriched molecules, carefully designed to maximize singlet order lifetimes. Recently, examples of singlet spin order in ^31^P (Korenchan et al., 2022) and ^103^Rh spin pairs (Harbor-Collins et al., 2024) were also reported.

In this paper, we report long-lived singlet spin order in molecules containing pairs of ^19^F nuclei coupled via non-bonded scalar interactions of “through-space” type. Accessing and exploiting singlet spin order in molecules containing pairs of ^19^F nuclei is interesting because ^19^F has a large gyromagnetic ratio (high NMR sensitivity), an essentially 100% natural abundance (no need for isotopic labelling), and because there is no endogenous fluorine in living organisms. This means there is no background signal in bio-medical applications, which contrasts with the intrinsic limitation in singlet NMR of molecules containing ^1^H pairs. However, a large chemical shift range of several hundreds of ppm’s and a typically small scalar coupling^1^ of molecules containing ^19^F spin pairs (Dolbier, 2016) has not played in favour of singlet order applications because this form of spin order is an eigenoperator of the Hamiltonian superoperator only in condition of nearly-magnetic-equivalence, i.e., when the chemical shift frequency difference between the two coupled nuclei is small compared to their spin mutual scalar coupling frequency. Conversely, some of the systems displaying “through-space” coupling, such as substituted 1,8-difluoronaphthalenes and 4,5-difluorophenanthrenes have the potential to meet the conditions for near-magnetic-equivalence, capitalising on their large “through-space” coupling, which is of the order of 170–190 Hz. In addition, and as exploited below, one can benefit from modern desktop NMR spectrometers and use their relatively low static magnetic field (typically less than 2 T) to reduce the chemical shift frequency difference between the two ^19^F spins to meet the conditions for near-magnetic-equivalence in a larger pool of ^19^F spin pairs, while maintaining good sensitivity and high spectral resolution characteristic of these machines.

## 2 Materials and methods

### 2.1 Molecular design, chemical synthesis and characterisation

**SCHEME 1:**
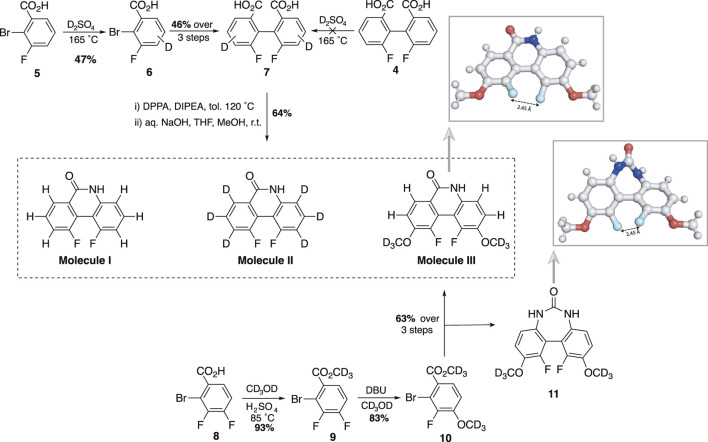
Synthesis of molecules **I**, **II**, **III** (DBU, 1,8-diazabicyclo (5.4.0)undec-7-ene; DPPA, diphenylphosphoryl azide; DIPEA, N,N-diisopropylethylamine; THF, tetrahydrofuran; Me, ${\text{CH}}_{3}$ ; with x-ray diffraction data for molecule **III** and urea **11** (grey, carbon/hydrogen; blue, nitrogen; red, oxygen; cyan, fluorine).

In seeking a molecular scaffold to support a ^19^F spin pair of nearly-magnetic-equivalence, we speculated, based on visual inspection, that molecule **I** might be a suitable candidate. Moreover, the synthetic route described by Murai et al. (2022). appeared amenable for modification such that hydrogen atoms providing deleterious singlet relaxation mechanisms may be substituted with alternative groups possessing more desirable magnetic properties for our purposes. Accordingly, we prepared molecule **I**, and derivative molecules **II** and **III**, bearing deuterium and methoxy- ${\text{d}}_{3}$ groups respectively (Scheme 1). Attempts to access molecule **II** by performing hydrogen/deuterium exchange on a relatively advanced precursor **4** of the synthetic route to molecule **I**, *via* electrophilic substitution under acidic conditions, was unsuccessful. However, treating commercially available acid **5** with the same conditions gave the trideuterobenzoic acid **6** as the major isotopologue, along with small amounts of the dideutero- and monodeutero-isotopologues (11 percent in each case), both as isotopomeric mixtures. Subjecting this material to the *Murai* synthetic route provided molecule **II**. Esterification of the 3,4-difluorobenzoic acid **8** with methanol- ${\text{d}}_{4}$ , followed by nucleophilic aromatic substitution with methanol- ${\text{d}}_{4}$ and DBU gave **10** as a single regioisomer in 77 percent yield over two steps. Again, following the route of *Murai* furnished molecule **III** along with a small amount of the urea **11**. Vapour diffusion provided diffraction quality single crystals of both molecule **III** and **11**, from which coordinates were collected^2^. For molecule **III**, the major occupant of the unit cell (c. 75 percent) has an F-F distance of 2.45 Å and the dihedral angle between the carbon fluorine bonds is 46.5° (see supplementary information).

### 2.2 NMR samples

Each of the three difluoro-substituted molecules **I**, **II** and **III** were dissolved in DMSO- ${\text{d}}_{6}$ and the solution transferred into a 5 mm OD LVP J-Young valved NMR tube for the NMR experiments described below. These solutions were degassed via ${\text{N}}_{2}$ bubbling to remove paramagnetic ${\text{O}}_{2}$ traces. The naming and details of the three samples so prepared are reported in Table 1 together with their main spin system parameters as obtained from ^19^F and ^19^F-{^1^H} spectra.

**TABLE 1:** Sample naming, formulation and spin system parameters.

Sample	Molecule	[ ] (M)	$J_{FF}$ (Hz)	$\Delta \delta_{FF}$ (ppm)
A	I	0.11	171.4	0.142
B	II	0.15	173.1	0.117
C	III	0.15	175.7	1.028

### 2.3 NMR experiments

**FIGURE 1:**
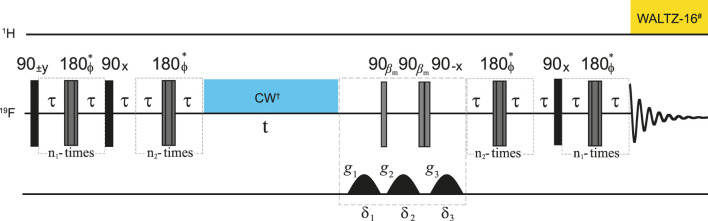
Pulse sequence used to measure the lifetime of singlet order in ^19^ ${\text{F}}_{2}$ -enriched molecules. ^#^ WALTZ-16 decoupling during acquisition was only used for sample A and C to decouple the protons. Experiments were done with and without CW decoupling during the time interval t as described in the text. Gradients have half-sinusoidal shape on Bruker and rectangular shape on Magritek spectrometers. Asterisks indicate a composite 180 ° pulse built as 90 × 180y90x. The phase ϕ is cycled as [x,x,-x,-x,-x,x,x,-x,-x,-x,x,x,x,-x,-x,x] within the train of 180 ° pulses. The total echo time is $\tau_{e}=\tau_{p}+2\tau =1/\left(2{\left(J^{2}+\Delta \omega^{2}\right)}^{1/2}\right)$ where $\tau_{p}=4*\tau_{90}$ is the duration of the composite pulse and $\tau_{90}$ the duration of the 90 ° pulse. $n_{1}=\pi J/\left(2\Delta \omega \right)$ , $n_{2}=n_{1}/2$ and $\beta_{m}=\text{arctan}\left(\sqrt{2}\right)$ .

NMR experiments where performed using two different NMR spectrometers: a 9.4 T Bruker magnet coupled to an Avance II console and equipped with a selective fluorine probehead for observation of ^19^F with ^1^H decoupling and z-gradients, and a 1.02 T Magritek SpinSolve 40 Carbon desktop spectrometer operating at 43.4 MHz for proton. Measurements of ${\text{T}}_{1}$ decay constants were performed using conventional inversion recovery methods (Hahn, 1949). For the measurement of singlet order decay constants, ${\text{T}}_{\text{S}}$ , we used the methodology based on magnetization-to-singlet pulse sequence schemes described elsewhere (Pileio, 2017) and shown in Figure 1. Note that ^19^F signal detection for Sample **A** and Sample **C** (see below) was done in the presence of ^1^H decoupling to collapse multiplets and obtain a neater signal from which to measure the decay constant (WALTZ-90° pulse duration $\tau_{w}^{1\text{H}}=65.7\mu \text{s}$ , corresponding to a nutation frequency of 3.8 kHz). The measurements of ${\text{T}}_{\text{S}}$ was done with and without a CW irradiation scheme (nutation frequency 2 kHz) applied on the ^19^F channel during the singlet storage time (t) in order to verify the presence of relaxation through scalar coupling of the second kind mechanism.

The parameters occurring in the pulse sequence in Figure 1 are reported in Table 2.

**TABLE 2:** The parameters used to run the pulse sequence in Figure 1.

Sample	Instrument	Field (T)	τ (ms)	$n_{1}$	$n_{2}$	$\left\{g_{1},g_{2},g_{3}\right\}$ (% of max)	$\left\{\delta_{1},\delta_{2},\delta_{3}\right\}$ (ms)	$\tau_{90}$ ( μ s)	$\tau_{w}^{1\text{H}}$ ( μ s)
A	AVII-400	9.4	1.385	4	2	{15,-15,-15}	{2.4,1.4,1.0}	7.0	65.7
B	AVII-400	9.4	1.400	6	3	{15,-15,-15}	{2.4,1.4,1.0}	7.0	-
C	Spinsolve 40	1.02	1.330	7	3	{100,-100,-100}	{4,2,2}	60.0	-

## 3 Results and discussion

### 3.1 Sample **A**

**FIGURE 2:**
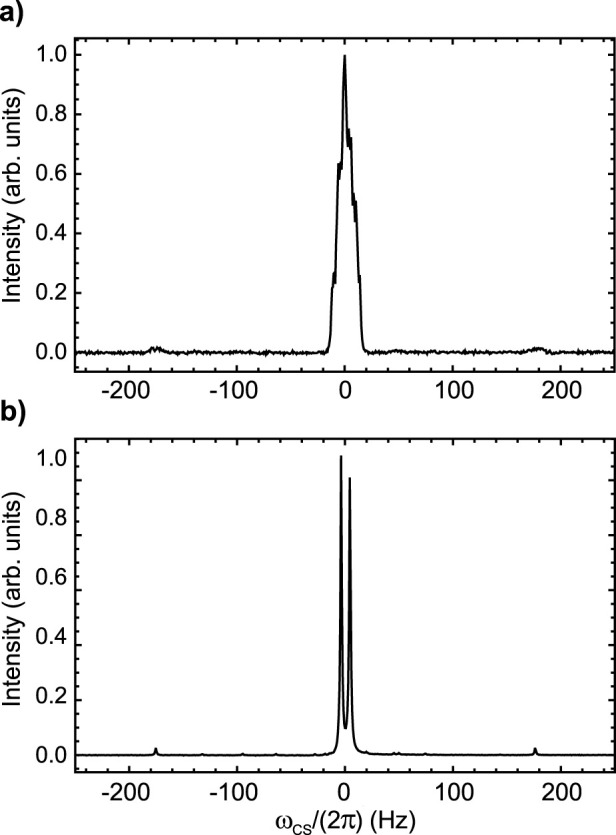
^19^F NMR spectra of Sample **A** taken at 9.4 T and 25°C without **(A)** and with **(B)** proton decoupling. The intensity is in arbitrary units and the peak has been centred at 0 Hz for simplicity (the chemical shift at the centre of the multiplet is −101.7 ppm).

The ^19^F-NMR spectrum of Sample **A**, taken at 9.4 T and 25°C, is reported in Figure 2A and shows a complex multiplet. The spectrum is shown using a frequency scale in Hz and has been centred at 0 Hz for simplicity (the chemical shift at the centre of the multiplet is −101.7 ppm). Based on visual inspection of the linewidth, it is clear that the fluorine nuclei are strongly coupled to each other and weakly coupled to possibly all other protons of the aromatic rings. This is easily confirmed by the ^19^F-{^1^ H } NMR spectrum of Sample **A**, taken at the same field and temperature (shown in Figure 2B), where a strong AB pattern is what remains of the ^19^F peaks after proton decoupling. From the frequency value of the four peaks in Figure 2B we measured a $J_{FF}=171.4$ Hz and a difference in chemical shift frequencies of the two fluorine atoms of $\Delta \delta_{FF}=0.142$ ppm.

It is therefore clear that the ^19^F spin pair is not isolated from the other spins in the molecule and all coupled spins should be considered in the discussion. The presence of scalar coupling to neighbouring protons may give rise to three types of problem: i) The spin Hamiltonian contains cross terms that connect the ^19^F singlet state to the triplet states. In fact, ^19^F singlet spin order may be not a good eigenoperator of the Hamiltonian superoperator; ii) The scalar couplings between ^19^F and ^1^H nuclei can give rise to relaxation via a scalar coupling of the second kind mechanism (S2K), which will shorten the lifetime of singlet order proportionally to the magnitude of the spin couplings involved and the size of the proton ${\text{T}}_{1}$ decay constant (Elliott et al., 2019; Pileio and Levitt, 2007); iii) The through-space dipolar couplings to the neighbouring protons give rise to an out-of-pair dipolar coupling relaxation mechanism (oDD) that contributes, and possibly dominates, the lifetime of ^19^F singlet order by reducing it by an extent that is proportional to the internuclear distances between ^19^F and ^1^H nuclei (Pileio, 2011).

Fortunately, the large value of $J_{FF}$ stabilises the singlet spin order between the two ^19^F spins and ^19^F singlet order is expected to remain a good eigenoperator of the spin Hamiltonian superoperator (Pileio and Levitt, 2007). The presence of a contribution to singlet relaxation due to scalar coupling of the second kind can be qualitatively probed (and, eventually, minimized) by applying a Continuous Wave (CW) irradiation on the ^19^F channel (or, equivalently, on the ^1^H or ^2^H channel) as explained in Elliott et al. (2019). Unfortunately, the out-of-pair dipole-dipole ^19^F-^1^H interactions cannot be suppressed or minimised with radio-frequency-based techniques and this is likely to be the major deleterious contribution to the size of the ^19^F singlet order lifetime in this sample. Clearly, such interactions can only be removed by chemical substitution - see below.

With this in mind, we measured the relaxation decay constant of ^19^F longitudinal spin order ( ${\text{T}}_{1}$ ) and that of ^19^F singlet spin order ( ${\text{T}}_{\text{S}}$ ) in Sample **A** at 9.4 T and 25 ° C (data reported in Table 3). While the presence of ^19^F singlet order is guaranteed by the singlet-filter in the pulse sequence (it only allows through singlet order) its decay constant is disappointingly short and comparable to ${\text{T}}_{1}$ . This is somewhat expected for the reasons explained above, i.e., because both scalar and dipolar couplings to the neighbouring protons generate a significant relaxation mechanism for both ${\text{T}}_{\text{S}}$ and ${\text{T}}_{1}$ . To probe the presence and importance of a S2K relaxation mechanism, we measured the singlet order decay constant ${\text{T}}_{\text{S}}$ using the pulse sequence in Figure 1, but where a CW irradiation scheme is applied during the singlet storage period (t) in order to decouple the protons from the fluorine atoms and therefore minimize an eventual S2K relaxation contribution (Elliott et al., 2019) (data reported in Table 3). Since the resultant value of ${\text{T}}_{\text{S}}$ was only marginally longer than that measured in the absence of proton irradiation (compare column 5 and 6 in Table 3), we conclude that a scalar coupling of the second kind relaxation mechanism is not the limiting factor for the ^19^F singlet order lifetime in sample **A**. It is therefore reasonable to think that the short relaxation decay constant observed for ^19^F singlet order in Sample **A** is due to an oDD mechanism proportional to the size of ^19^F-^1^H dipole-dipole couplings in the molecule. Clearly, we expect to have a contribution from chemical shift anisotropy (CSA) relaxation, a known important source of relaxation in ^19^F-NMR spectroscopy (Gerig, 2001), but, given the small difference between the ${\text{T}}_{1}$ at the two magnetic fields, this may not be the dominant one in this sample. Note that the chemical shift anisotropy mechanism acts differently on ${\text{T}}_{\text{S}}$ than on ${\text{T}}_{1}$ (Pileio, 2017) so the conclusion above can be challenged; however, it has not been possible to measure the ${\text{T}}_{\text{S}}$ at 1.02 T for Sample **A** because of the large number of echoes required in the pulse sequence (due to the small chemical shift frequency difference compared to the very large scalar coupling) and the relatively short ${\text{T}}_{2}$ .

**TABLE 3:** The^19^F ${\text{T}}_{1}$ and ${\text{T}}_{\text{S}}$ decay constants measured for all samples in this work. Measurements at 1.02 T were taken at 27 ° C while those at 9.4 T were taken at 25 ° C.

Sample	${\text{T}}_{1}$ (1.02 T)	${\text{T}}_{1}$ (9.4 T)	${\text{T}}_{\text{S}}^{*}$ (1.02 T)	${\text{T}}_{\text{S}}^{*}$ (9.4 T)	${\text{T}}_{\text{S}}^{†}$ (9.4 T)
A	2.92 ± 0.23	1.26 ± 0.03	-	1.60 ± 0.05	1.65 ± 0.04
B	2.74 ± 0.17	1.34 ± 0.02	-	2.26 ± 0.03	2.31 ± 0.03
C	2.25 ± 0.11	0.48 ± 0.01	29.1 ± 2.4	0.53 ± 0.0 $1^{**}$	1.61 ± 0.0 $5^{‡}$

^*^ measured without CW, irradiation during t;^†^ measured with CW, irradiation during t.^**^ measured with pulse sequence in Sarkar et al. (2007);^‡^ measured with pulse sequence in Sarkar et al. (2007) and CW, irradiation on^19^F channel during t.

**FIGURE 3:**
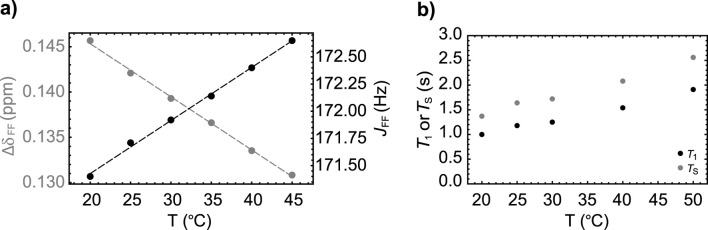
**(A)** The temperature dependence of the chemical shift difference between the two fluorine nuclei (gray circles) and their mutual scalar coupling constant (black circles) in Sample **A**. Dashed lines are best fit to the experimental points. **(B)** The variation of ${\text{T}}_{1}$ (black circles) and ${\text{T}}_{\text{S}}$ (gray circles) with temperature measured on Sample **A**.

Given that the non-bonding nature of the ^19^F-^19^F scalar coupling relies on partial orbital overlap, we decided to explore whether the magnitude of the $J_{FF}$ coupling depends upon temperature. To do so, we monitored the value of the ^19^F-^19^F scalar coupling and the difference in chemical shift between the two fluorine nuclei over a small temperature range (see Figure 3A). We observe a small linear decrease (slope = −0.586 ppb/°C) in the chemical shift difference as the temperature is increased. Similarly, the value of $J_{FF}$ increases linearly, but slowly, with temperature (slope = 0.05 Hz/°C). Additionally, we measured ${\text{T}}_{1}$ and ${\text{T}}_{\text{S}}$ decay constants at some selected temperature values between 20°C and 50°C, finding that both these constants increase almost linearly with temperature, although not by much (see Figure 3B). Such a trend correlates well with the reduction in viscosity of the solvent (literature data on (CH_3_)_2_SO show a reduction from 2.24 cP to 1.286 cP as the temperature is increased from 20 to 50 ° C).

### 3.2 Sample **B**

**FIGURE 4:**
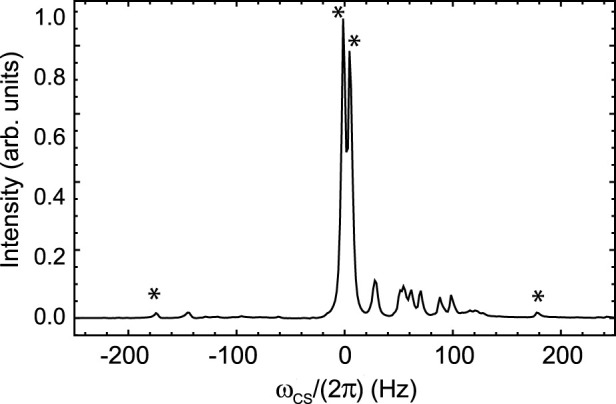
^19^F NMR spectra of Sample **B** taken at 9.4 T and 25°C. The intensity is in arbitrary units and the peak has been centred at 0 Hz for simplicity (the chemical shit at the centre of the AB pattern is −101.8 ppm). The expected AB pattern arising from the ^19^F spin pair is marked with asterisks. All unmarked peaks arise from isotopomers containing protons in place of deuterium.

In order to remove dipolar interactions with the neighbouring proton, we synthesised molecule **II** (prepared as Sample **B**, see Materials and Methods), which is an analogue of molecule **I** with the aromatic ring hydrogens substituted with deuteriums so to scale down the size of the ^19^F dipolar couplings by a factor of about six (the ratio between hydrogen and deuterium gyromagnetic ratios). The ^19^F-NMR spectrum of Sample **B**, taken at 9.4 T and 25°C, is reported in Figure 4, where a set of asterisks mark the four peaks of the expected AB pattern for the two fluorine peaks in the sample (couplings to neighbouring deuterium nuclei fall within the linewidth). Other than these four marked peaks, the spectrum shows a series of other minor peaks that are due to the trace isotopologues containing mixtures of hydrogen and deuterium atoms (see Section 2.1 and supplementary information).

A measurement of the longitudinal order decay constant at 9.4 T and 25°C on this sample resulted in a rather short value of ${\text{T}}_{1}=1.31\pm 0.02$ , which is only marginally longer than the one found for Sample **A** (see Table 3). This is not surprising since the value of ${\text{T}}_{1}$ for these two samples are expected to be dominated by the ^19^F-^19^F dipolar interaction relaxation mechanism. Unfortunately, the measurement of the singlet order decay constant in this sample, done in the same conditions and using the pulse sequence in Figure 1 and the parameters in Table 2, still resulted in a rather short value of ${\text{T}}_{\text{S}}$ that is only marginally longer than ${\text{T}}_{1}$ by a factor of 1.7 (see Table 3). These results led us to hypothesise that either the CSA relaxation mechanism dominates, or in substituting hydrogens with deuteriums we may have gained by minimising dipolar coupling interactions to hydrogens but reintroduced a more serious S2K mechanism. Unfortunately, it has not been possible to measure the ${\text{T}}_{\text{S}}$ of Sample **B** at 1.02 T, for the same reasons occurring in Sample **A**, nor the CSA contribution can be easily calculated in absence of the full chemical shift tensors of the two fluorine nuclei. Similarly, the S2K contribution depends on both the size of the scalar coupling between the fluorines and the deuterons and the relaxation time of these latter nuclei (for deuterium this is dominated by its quadrupolar coupling interaction) and therefore it is not easy to predict whether a deuterium is actually worse than a hydrogen in the vicinity of the fluorine, as it could be the case here. Fortunately, one can experimentally probe the presence of the S2K mechanism by measuring the ${\text{T}}_{\text{S}}$ decay constant with the pulse sequence in Figure 1 and in the presence of a CW irradiation applied during the time interval t. The results, obtained with a CW nutation frequency of 2 kHz applied on the ^19^F channel and reported in Table 3, did not show an extension in ${\text{T}}_{\text{S}}$ and therefore led us to conclude that S2K is not a limiting relaxation mechanism for Sample **B**.

**FIGURE 5:**
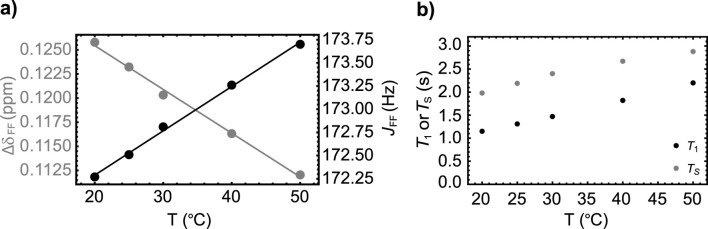
**(A)** The variation with temperature of the chemical shift difference between the two fluorine nuclei (gray circles) and their mutual scalar coupling constant (black circles) in Sample **B**. Dashed lines are best fit to the experimental points. **(B)** The variation of ${\text{T}}_{1}$ (black circles) and ${\text{T}}_{\text{S}}$ (gray circles) with temperature measured on Sample **B**.

As done for Sample **A**, we report the results of a series of experiments to study the temperature dependence of the spin system parameters (Figure 5A) and the relaxation decay constants ${\text{T}}_{1}$ and ${\text{T}}_{\text{S}}$ (see Figure 5B) for Sample **B**. Similarly to what seen in the previous section, even for Sample **B** we found a slow decrease (slope = −0.454 ppb/ ° C) of the difference in chemical shift between the two fluorine nuclei and a slow increase in the value of the ^19^F-^19^F scalar coupling (slope = 0.048 Hz/°C) as temperature is increased. Moreover, both ${\text{T}}_{1}$ and ${\text{T}}_{\text{S}}$ increase slightly with temperature but this seems again consistent with the decrease in solvent viscosity over that temperature range.

### 3.3 Sample **C**

**FIGURE 6:**
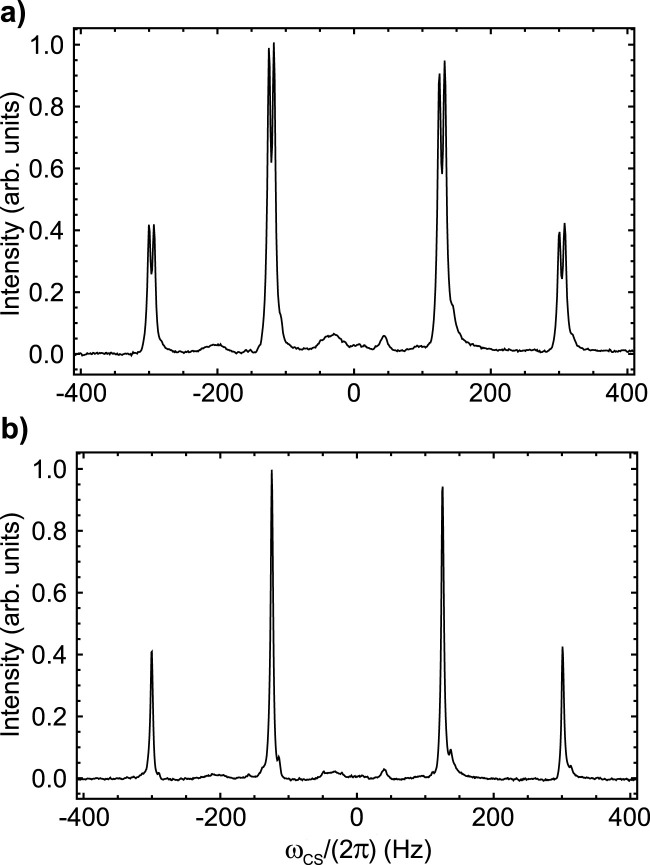
^19^F NMR spectra of Sample **C** taken at 9.4 T and 25°C without **(A)** and with **(B)** proton decoupling. The intensity is in arbitrary units and the peak has been centred at 0 Hz for simplicity (the chemical shit at the centre of the multiplet is −124.6 ppm).

With the aim of removing any immediate nuclear spin coupled to the fluorine atoms, we synthesised a third molecule (molecule **III** in Scheme 1, prepared as Sample **C** as described in Materials and Methods) where we aimed to replace the closest spin to the two fluorine nuclei with - ${\text{OCD}}_{3}$ groups. The ^19^F-NMR spectrum of Sample **C**, taken at 9.4 T and 25°C, is reported in Figure 6A. It consists of an AB pattern where each transition is further split into two. This is compatible with a strongly-coupled ^19^F spin pair which is further coupled to one proton on the aromatic ring (and possibly to the other, although with a scalar coupling that falls within the linewidth). The pattern is compatible with these spin system parameters: $J_{FF}=175.7$ Hz, $\Delta \delta_{FF}=1.028$ ppm and $J_{FH}=7.5$ Hz. The proton-decoupled fluorine spectrum of the same sample is reported in Figure 6B and shows the simple AB pattern expected for the proton-decoupled ^19^F spin pair. The relatively large chemical shift difference between the two fluorine atoms corresponds to a chemical shift frequency difference of 387.2 Hz at 9.4 T. At such magnetic field, the condition $J_{FF}<\Delta \omega_{FF}$ is not met and therefore singlet order is not a good eigenoperator of the spin Hamiltonian anymore and an RF irradiation (Pileio and Levitt, 2009) is required in order to *lock* such form of order. Moreover, the pulse sequences in Figure 1, designed for strongly-coupled/nearly-equivalent spin pairs, performs badly at producing singlet order in this regime. For this reason the pulse sequence reported by Sarkar et al. (2007) was used to measure the ${\text{T}}_{\text{S}}$ of Sample **C** at 9.4 T. The resulting ${\text{T}}_{\text{S}}$ values, reported in Table 3 with and without CW irradiation on the ^19^F channel, still show a relatively short singlet lifetime which is longer than ${\text{T}}_{1}$ by only a factor of around three. Note the importance of the CW decoupling which is in this case required because of the presence of a large chemical shift difference between the two fluorine spins (Pileio and Levitt, 2009).

At this point, we found it very interesting to prepare ^19^F singlet spin order in the low magnetic field of a desktop spectrometer operating at 1.02 T, where the chemical shift frequency difference is only 42.1 Hz and the two fluorine nuclei would remain strongly-coupled. For this, we coded the pulse sequence in Figure 1 into a Spinsolve and measured both ${\text{T}}_{1}$ and ${\text{T}}_{\text{S}}$ values of Sample **C** at 1.02 T, data reported in Table 3. The value of ${\text{T}}_{1}$ is essentially the same as in the other two samples, again this may simply reinforce the idea that ${\text{T}}_{1}$ in these molecules is dominated by the dipolar interaction between the two fluorine nuclei, with a small contribution from CSA (Ahuja et al., 2007; Pileio et al., 2012; Stevanato et al., 2015) (compare the values of ${\text{T}}_{1}$ at the two magnetic fields for all three molecules reported in columns 2 and 3 of Table 3). ${\text{T}}_{\text{S}}$ at 1.02 T, however, is appreciably longer than ${\text{T}}_{1}$ of the same sample at the same field, and also longer than the values of ${\text{T}}_{\text{S}}$ measured for the other two samples in this paper. While there is a clear contribution from CSA in determining such a long lifetime, this alone cannot justify the measured value (CSA scales with the square of the magnetic field). Hence, the mechanism(s) that set the limit to the observed value of ${\text{T}}_{\text{S}}$ are not fully clear from this set of experiments and further work needs to be done in terms of experimental measurements and numerical simulations. Preliminary relaxation data taken on ^1^H and ^13^C nuclei in these molecules may suggest the presence of dimerization (or some other form of molecular aggregation) that, if present, would reduce the molecular tumbling correlation time and therefore scale down the absolute value of both ${\text{T}}_{1}$ and ${\text{T}}_{\text{S}}$ achievable in these molecules. Given the nature of the scalar coupling in these systems, arising from orbital overlap under steric constraints, it is also possible that scalar coupling of the first kind (S1K) plays a role in determining the size of singlet relaxation decay constants.

## 4 Conclusion

We have demonstrated that the strong scalar coupling between ^19^F spin pairs separated by many bonds, but constrained in spatial proximity, can be deployed to prepare long-lived singlet spin order. In proof of principle experiments conducted on three newly-synthesized molecules containing spatially-constrained ^19^F pairs that display very large scalar couplings of “through-space” nature, we have shown a 13-fold extension in spin memory. The work has evidenced a significant contribution of chemical shift anisotropy to singlet order relaxation and hinted at the possibility of other complementary mechanisms such as scalar coupling of the first kind or the presence of molecular aggregation. A full understanding of these relaxation contributions requires supplementary experiments and numerical simulations that are the subject of future work. For example, it would be interesting to run field-cycling experiments to measure ${\text{T}}_{1}$ and ${\text{T}}_{\text{S}}$ over a larger ranges of magnetic fields so to figure out the CSA contribution, but this would require a fast sample shuttle coupled to a fluorine probe, currently not available in our laboratory. A second generation molecular core might be a better candidate to investigate the underlying relaxation mechanisms. The lack of endogenous ^19^F in the human body and the high sensitivity achievable in ^19^F NMR mean that singlet spin order prepared on ^19^F spin pairs has potentially far reaching applications in the field of magnetic resonance imaging and molecular tracing.

## Data Availability

CCDC 2390768-2390769 contains the supplementary crystallographic data for this paper. The data can be obtained free of charge from The Cambridge Crystallographic Data Centre via www.ccdc.cam.ac.uk/structures.

## Footnotes

1 The ^19^F-^19^F scalar coupling can be quite large in vicinal ^19^F pairs but then the ^19^F nuclei are often chemically equivalent and therefore singlet order cannot be accessed.

2 CCDC #2390769 (molecule **III**), #2390768 (**11**).
