# Supplementary material for: Singlet spin order in spin pairs coupled via non-bonded interactions
Source: Front Chem. 2025 Jan 30;12:1511720. doi: 10.3389/fchem.2024.1511720 (PMC11821957; doi:10.3389/fchem.2024.1511720)

Supplementary Material

Singlet spin order in spin pairs via non-bonded interactions

Giuseppe Pileio,*[1] Dolnapa Yamano,[1] Craig Eccles,[2] Graham J. Tizzard,[3] and Sam Thompson*[1]

[1] *School of Chemistry, University of Southampton, Southampton, SO17 1BJ, United Kingdom*

[2] *Magritek GmbH, Philipsstraße 8, 52068 Aachen, Germany*

[3] *UK National Crystallography Service, School of Chemistry, University of Southampton, Southampton, SO17 1BJ, United Kingdom*

Correspondence: g.pileio@soton.ac.uk and st3a15@soton.ac.uk

Table of Contents

1. GENERAL EXPERIMENTAL 2

1.1 SOLVENTS AND REAGENTS 2
1.2 CHROMATOGRAPHY 2
1.3 SPECTROSCOPY 2
1.4 CRYSTALLOGRAPHY 3

2 EXPERIMENTAL PROCEDURES AND CHARACTERISATION DATA 3

2.1 MOLECULE I: 1,10-DIFLUOROPHENANTHRIDIN-6(5*H*)-ONE 3
2.2 MOLECULE II: 1,10-DIFLUOROPHENANTHRIDIN-6(5*H*)-ONE-2,3,4,7,8,9-*D*6 4
2.3 MOLECULE III: 1,11-DIFLUORO-2,10-BIS(METHOXY-*D*3)-5,7-DIHYDRO-6*H*-DIBENZO[*D*,*F*][1,3]DIAZEPIN-6-ONE 10

3. X-RAY CRYSTALLOGRAPHY 17

4. REFERENCES 20

5. NMR SPECTRA 21

1. General Experimental

1.1 Solvents and Reagents

Reactions were carried out under an argon atmosphere in oven-dried glassware unless otherwise stated. Standard inert atmosphere techniques were used in handling all air- and moisture-sensitive reagents. All reagents were purchased from Sigma-Aldrich, ThermoFisher Scientific, or Fluorochem and used without further purification. Anhydrous and deuterated solvents were purchased from Merck or Fluorochem and were used as supplied. Aqueous solutions are saturated unless specified otherwise.

1.2 Chromatography

Flash column chromatography was carried out using Merck 60 silica gel. Thin-layer chromatography was carried out using Merck Kieselgel 60 F254 (230-400 mesh) fluorescent treated silica, visualized under UV light (254 nm) or by staining with aqueous potassium permanganate solution, ninhydrin or ceric ammonium molybdate solutions.

1.3 Spectroscopy

^{1}H, ^{2}H, ^{13}C, and ^{19}F NMR spectra were recorded using a Bruker spectrometer (500MHz) running TopSpin™ software and are quoted in ppm for measurement against residual solvent peaks or $Si(CD_3)_4$. Chemical shifts (δ) are given in parts per million (ppm) and coupling constants (*J*) are given in Hertz (Hz). The ^{1}H and 2H NMR spectra are reported as follows: δ (number of hydrogens/deuteriums, multiplicity, coupling constant). Multiplicity is abbreviated as follows: s = singlet, d = doublet, t = triplet, q = quartet, quint. = quintet, m = multiplet, br = broad. Systematic compound names are those generated by ChemDraw™ (CambridgeSoft) following IUPAC nomenclature. The numbering scheme used for NMR assignment is arbitrary and does not follow any convention. Numbering of compounds is illustrated on the spectra themselves, *vide infra*. The ^{2}H, ^{13}C and ^{19}F NMR spectra are reported in δ / ppm. ^{13}C and ^{19}F NMR spectra are proton decoupled unless specified otherwise. Where necessary, two-dimensional (COSY, HSQC, HMBC) NMR experiments were used to assist the assignment of signals in the ^{1}H and ^{13}C NMR spectra.

High-resolution ESI mass spectra were undertaken at the University of Southampton using a MaXis (Bruker Daltonics, Bremen, Germany) time of flight (TOF) mass spectrometer or a solariX (Bruker Daltonics, Bremen, Germany) mass spectrometer equipped with a 4.7 T magnet and FT-ICR cell.

Infra-red (IR) spectra were recorded using a ThermoFisher Scientific Nicolet iS5 spectrometer from a thin film deposited onto a diamond ATR module. Sixteen scans were acquired at a resolution of 4 cm^{-1}. Only selected maximum absorbances (ν_{max}) of the most intense peaks are reported (cm^{-1}).

Melting points were recorded using a Stuart (Bibby Scientific Limited) SMP20 machine and reported uncorrected in degrees Celsius (˚C).

1.4 Crystallography

Data were collected from crystals mounted on a MITEGEN holder in oil using a Rigaku 007HF diffractometer with Arc)Sec VHF Varimax (**Molecule III**) or HF Varimax (**11**) confocal mirrors and a UG2 goniometer and HyPix Arc-100 detector equipped with an Oxford Cryosystems low-temperature device operating at T = 100(2) K.[1] The structures were solved with the ShelXT 2018/2[2,3] solution program using dual methods and by using Olex2 1.5-dev[4] as the graphical interface. The models were refined with olex2.refine 1.5-dev[5] (**Molecule III**) or ShelXL 2018/3[2,3] (**11**) using full matrix least squares minimisation on $\boldsymbol{F}^2$.

2 Experimental Procedures and Characterisation Data

2.1 Molecule I: 1,10-difluorophenanthridin-6(5*H*)-one

Prepared according to the procedure of Murai *et al.*[6]

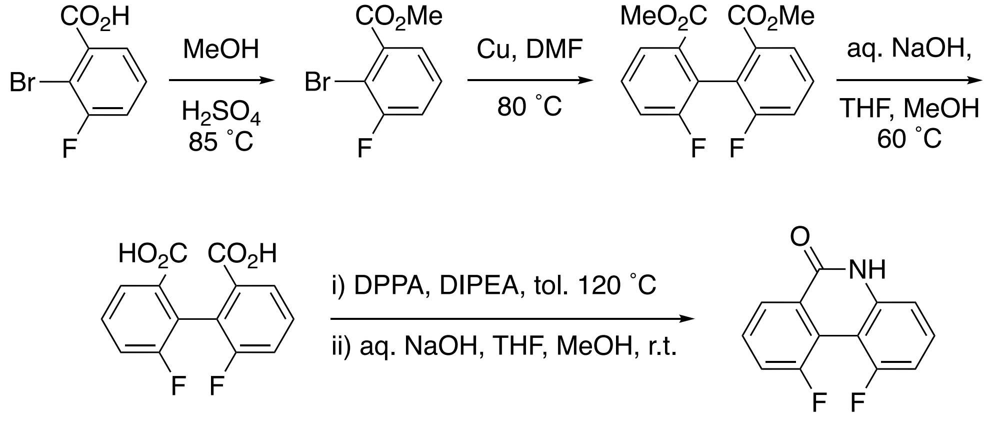

Molecule I

Scheme S1. Synthetic route to **Molecule I**.

To a stirred solution of 6,6'-difluoro-[1,1'-biphenyl]-2,2'-dicarboxylic acid (0.110 g, 0.40 mmol) in toluene (8.0 mL), diphenylphosphoryl azide (DPPA, 0.17 mL, 0.80 mmol) and *N*,*N*-diisopropylethylamine (0.42 mL, 2.40 mmol) were added. After heating for 16 h at 120 ˚C, the

reaction mixture was concentrated *in vacuo*. A solution of the resulting residue in THF/MeOH/2 *N* aq. NaOH (1:1:1, 25 mL) was stirred at room temperature for 16 h, and the reaction mixture was extracted with ethyl acetate (20 mL). The organic layer was separated, washed with water (15 mL) and brine (15 mL), dried over magnesium sulfate, filtered, and concentrated *in vacuo*. The resulting residue was recrystallized (diethyl ether/hexane, 3:97) to give *the title compound* ***Molecule I*** (0.06 g, 0.26 mmol, 65% yield) as a white solid.

R_f 0.32 (ethyl acetate/petroleum ether, 4/6).

δ_H (500 MHz, $(CD_3)_2SO$) 11.96 (1H, s), 8.24-8.20 (1H, m), 7.77- 7.72 (1H, m), 7.59-7.54 (1H, m), 7.23 (1H, dd, *J* 8.2, 1.1), 7.13- 7.08 (1H, m).

δ_C (126 MHz, $(CD_3)_2SO$) 159.6 (d, *J* 1.6), 159.6- 156.6 (2C, m), 138.6 (dd, *J* 4.6, 2.3), 131.1 (dd, *J* 7.6, 4.0), 129.9 (dd, *J* 6.6, 3.5), 128.7, 123.6, 120.6 (dd, *J* 17.8, 7.7), 118.9 (dd, *J* 9.7, 2.1), 111.9, 109.7 (dd, *J* 16.3, 9.0), 103.2 (dd, *J* 10.9, 4.1).

δ_F {^{1}H} (471 MHz, $(CD_3)_2SO$) –101.38 (d, *J* 171.69), –101.78 (d, *J* 171.69).

MP 309-312 (lit = >300).[6]

2.2 Molecule II: 1,10-difluorophenanthridin-6(5*H*)-one-2,3,4,7,8,9-d_6

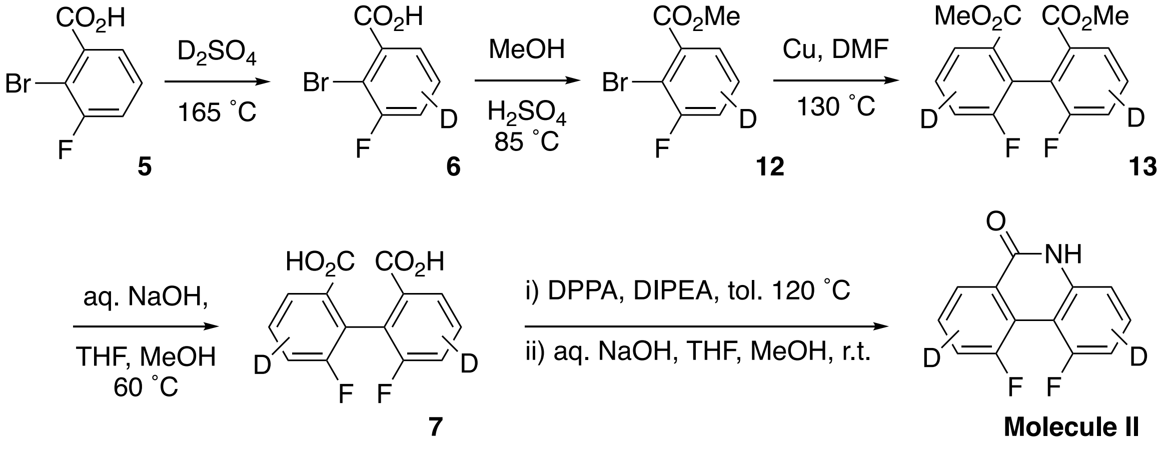

Scheme S1. Synthetic route to **Molecule II**.

2-Bromo-3-fluorobenzoic-4,5,6-*d*$_3$ acid 6

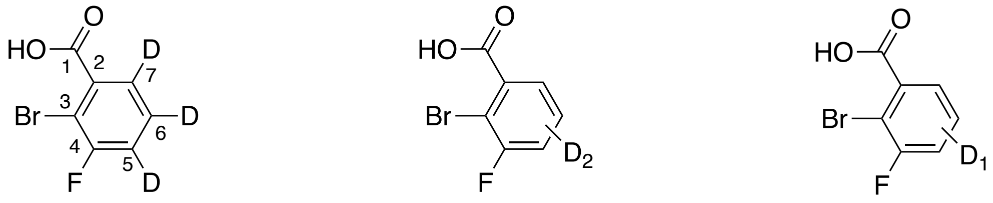

ratio 78 11 11

Based on a procedure by Perrin *et al*,[7] a stirred solution of 2-bromo-3-fluorobenzoic acid **5** (2.50 g, 11.42 mmol) in D_2SO_4 (8.5 mL) was heated at 165 °C for 72 hours. After cooling to room temperature, the resultant dark brown solution was slowly poured into ice and extracted with diethyl ether (3 x 80 mL). The organic layers were combined, dried over magnesium sulfate, filtered, and concentrated *in vacuo* to give a brown solid. The resulting residue was recrystallised (acetone/diethyl ether, 10/90) to give *the title compound* ***6*** (1.20 g, 5.4 mmol, 47% yield) as a white solid. Integration of resonances in the ^{19}F NMR spectrum indicated a 78:11:11 ratio of 2-bromo-3-fluorobenzoic-*d*$_3$ acid, 2-bromo-3-fluorobenzoic-*d*$_2$ acid and 2-bromo-3-fluorobenzoic-*d*$_1$ acid respectively. The mixture was carried forward for subsequent reactions with spectral data given for the major (*d*$_3$) isomer.

R_f 0.30 (ethyl acetate/petroleum ether, 4/6).

MP 158-160.

Spectral data for 2-bromo-3-fluorobenzoic-*d*$_3$ acid **6** only:

δ_H (500 MHz, $CDCl_3$) 9.19 (1H, br, OH).

δ_C (126 MHz, $CDCl_3$) 170.5 (d, *J* 2.6, C1), 159.9 (d, *J* 247.3, C4), 132.5 (d, *J* 12.1, C2), 128.6-127.3 (m C6), 126.7-126.1 (C7), 120.5-119.8 (C5), 110.6 (d, *J* 22.6, C3).

δ_F {^{1}H} (471 MHz, $CDCl_3$) –102.38.

δ_D (77 MHz, $CHCl_3$) 7.89 (1D, br, D7), 7.48 (1D, br, D6), 7.42 (1D, br, D5).

ν_{max} 2859, 2600, 1695, 1553, 1490, 1428, 1382, 1294, 1265, 1024, 983, 927, 896, 790, 676.

HRMS (ESI) found $[M+H]^-$ 219.9491, $C_7D_3{}^{79}BrFO_2$ requires 219.9494.

Methyl 2-bromo-3-fluorobenzoate-4,5,6-d_3 12

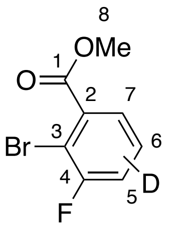

To a solution of 2-bromo-3,4-difluorobenzoic acid **6** (1.20 g, 5.40 mmol) in methanol (5.4 mL), at 0 °C, concentrated H_2SO_4 (0.11 mL) was added dropwise. The reaction mixture was heated to 85 °C for 16 h. After cooling to room temperature, the reaction mixture was concentrated *in vacuo*. The residue was suspended in ethyl acetate (40 mL) and water (80 mL) was added. The organic layer was separated and washed sequentially with $NaHCO_3$ solution (40 mL), water (40 mL), brine (40 mL), and dried over magnesium sulfate, filtered, and concentrated *in vacuo*. The residue was purified by flash column chromatography (ethyl acetate/petroleum ether, 5/95) to give *the title compound **12*** (1.19 g, 5.04 mmol, 93% yield) as a colourless oil.

R_f 0.33 (ethyl acetate/petroleum ether, 1/20).

δ_H (500 MHz, $CDCl_3$) 3.93 (3H, s, H8).

δ_C (126 MHz, $CDCl_3$) 165.9 (d, *J* 2.9, C1), 159.6 (d, *J* 247.3, C4), 134.3 (d, *J* 12.9, C2), 128.5-127.8 (m C6), 126.7-126.1 (C7), 119.3-118.6 (C5), 109.5 (d, *J* 22.5, C3), 52.8 (C8).

δ_F {^{1}H} (471 MHz, $CDCl_3$) –103.19.

δ_D (77 MHz, $CHCl_3$) 7.69 (1D, br, D7), 7.46 (1D, br, D6), 7.37 (1D, br, D5).

ν_{max} 2954, 1730, 1553, 1437, 1416, 1390, 1241, 1196, 1167, 1026, 381, 896, 789, 677, 619, 582.

HRMS (ESI) found $[M+H]^+$ 235.9789, $C_8H_4D_3{}^{79}BrFO_2$ requires 235.9796.

Dimethyl 6,6'-difluoro-[1,1'-biphenyl]-2,2'-dicarboxylate-3,3',4,4',5,5'-d_6 13

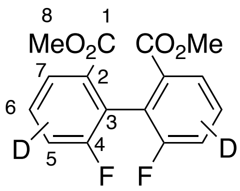

A solution of methyl 2-bromo-3-fluorobenzoate-4,5,6-d_3 **12** (1.14 g, 4.83 mmol) and copper(0) powder (2.46 g, 38.64 mmol) in *N,N*-dimethylformamide (5.0 mL) was refluxed at 130 °C for 4 hours. After the reaction was cooled to room temperature, the solids were removed by filtration. Water (80 mL) was added to the eluent, and it was extracted with dichloromethane (40 mL). The organic layer was washed with brine (40 mL), dried over magnesium sulfate, filtered, and concentrated *in vacuo*. The residue was purified by flash column chromatography (ethyl acetate/petroleum ether, 4/6) to give *the title compound **13*** (0.43 g, 1.35 mmol, 51% yield) as a white solid.

R_f 0.33 (ethyl acetate/petroleum ether, 1/4).

δ_H (500 MHz, $CDCl_3$) 3.70 (6H, s, H8).

δ_C (126 MHz, $CDCl_3$) 166.1 (d, *J* 3.2, C1), 159.7 (d, *J* 244.8, C4), 131.5 (d, *J* 11.4, C2), 129.5-128.7 (m, C6), 126.2-125.6 (C7), 123.9 (d, *J* 18.9, C3), 119.3-118.6 (C5), 52.2 (C8).

δ_F {^{1}H} (471 MHz, $CDCl_3$) –113.13.

δ_D (77 MHz, $CHCl_3$) 7.85 (1D, br, D7), 7.41 (1D, br, D6), 7.27 (1D, br, D5).

ν_{max} 3008, 2954, 1719, 1559, 1434, 1381, 1243, 1195, 1176, 1017, 989, 899, 785, 709, 607.

HRMS (ESI) found $[M+Na]^+$ 335.0964, $C_{16}H_6D_6F_2NaO_4$ requires 335.0972.

MP 116-118.

6,6'-Difluoro-[1,1'-biphenyl]-3,3',4,4',5,5'-d_6-2,2'-dicarboxylic acid 7

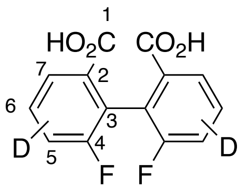

To a solution of bis(methyl-d_3) 6,6'-difluoro-[1,1'-biphenyl]-2,2'-dicarboxylate-3,3',4,4',5,5'-d_6 **13** (0.34 g, 1.08 mmol) in THF/MeOH (2:1, 5.0 mL), 2 *N* aqueous NaOH (1.3 mL) was added at room temperature. After stirring at 60 °C for 16 h, the reaction mixture was washed with diethyl ether (20 mL). The aqueous layer was acidified with 2 *N* aqueous HCl, until pH2 was attained, followed by extraction with ethyl acetate (20 mL). The organic layer was washed with brine (20 mL), dried over magnesium sulfate, filtered, and concentrated *in vacuo* to give *the title compound 7* (0.30 g, 1.04 mmol, 96% yield) as a white solid.

R_f 0.33 (ethyl acetate/petroleum ether, 4/1).

δ_C (126 MHz, CD_3OD) 168.7 (d, *J* 2.5, C1), 161.1 (d, *J* 242.9, C4), 133.6 (d, *J* 9.6, C2), 130.2-129.6 (m C6), 127.2-126.7 (C7), 125.4(d, *J* 18.7, C3), 119.8-119.1(C5).

δ_F {^{1}H} (471 MHz, CD_3OD) –111.64.

δ_D (77 MHz, CH_3OH) 8.70 (1D, br, D7), 8.30 (1D, br, D6), 8.16 (1D, br, D5).

ν_{max} 2854, 2621, 1733, 1681, 1588, 1550, 1435, 1377, 1251, 1017, 930, 841, 757, 693, 677.

HRMS (ESI) found $[M+H]^-$ 283.0700, $C_{14}HD_6F_2O_4$ requires 283.0694.

MP 310-314.

1,10-Difluorophenanthridin-6(5*H*)-one-2,3,4,7,8,9-d_6 'Molecule II'

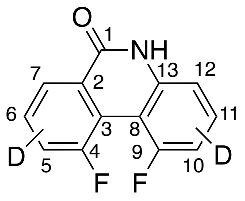

To a solution of 6,6'-difluoro-[1,1'-biphenyl]-3,3',4,4',5,5'-d_6-2,2'-dicarboxylic acid **7** (0.15 g, 0.53 mmol) in toluene (10 mL), diphenylphosphoryl azide (0.23 mL, 1.05 mmol) and *N*,*N*-diisopropylethylamine (0.55 mL, 3.16 mmol) were added at room temperature. After refluxing for 16 h, the reaction mixture was concentrated *in vacuo*. A solution of the resulting residue in THF/MeOH/2 *N* aqueous NaOH (1:1:1, 25 mL) was then stirred at room temperature for 16 h, and the reaction mixture was extracted with ethyl acetate (20 mL). The organic layer was separated, washed with water (40 mL) and brine (40 mL), dried over magnesium sulfate, filtered, and concentrated *in vacuo*. The resulting residue was recrystallised (diethyl ether/hexane, 3/97) to give *the title compound* ***'Molecule II'*** (0.80 g, 0.34 mmol, 64% yield) as a white solid.

R_f 0.32 (ethyl acetate/petroleum ether, 4/6).

δ_H (500 MHz, $(CD_3)_2SO$) 11.94 (1H, s, NH).

δ_C (126 MHz, $(CD_3)_2SO$) 160.0 (C1), 159.7 (d, *J* 250.8, C9), 158.1 (d, *J* 255.3, C4), 139.0 (d, *J* 4.5, C13), 131.5-131.4 (C11), 130.0-129.5 (C6), 129.1 (d, *J* 7.7, C2), 124.0-123.5 (C7), 121.0-120.6 (C5), 119.4 (dd, *J* 9.7, 2.1, C3), 112.4-111.7 (C12), 110.2-109.5 (C10), 103.7 (dd, *J* 10.6, 4.5, C8).

δ_F {^{1}H} NMR (471 MHz, $(CD_3)_2SO$) –101.78 (dd, *J* 172.3), –102.00 (dd, *J* 172.3).

δ_D (77 MHz, $(CH_3)_2SO$) 7.86 (1D, br, CD), 7.39 (1D, br, CD), 7.36 (1D, br, CD), 7.19 (1D, br, CD), 6.85 (1D, br, CD), 6.74 (1D, br, CD).

ν_{max} 3010, 2854, 1979, 1662, 1594, 1540, 1496, 1456, 1394, 1347, 1312, 1266, 1236, 1222, 1104, 997, 879, 822, 787, 740, 665.

HRMS (ESI) found $[M+H]^+$ 238.0940, $C_{13}H_2D_6F_2NO$ requires 238.0945.

MP 314-316 (decomposition).

2.3 Molecule III: 1,11-difluoro-2,10-bis(methoxy-*d*3)-5,7-dihydro-6*H*-dibenzo[*d*,*f*][1,3]diazepin-6-one

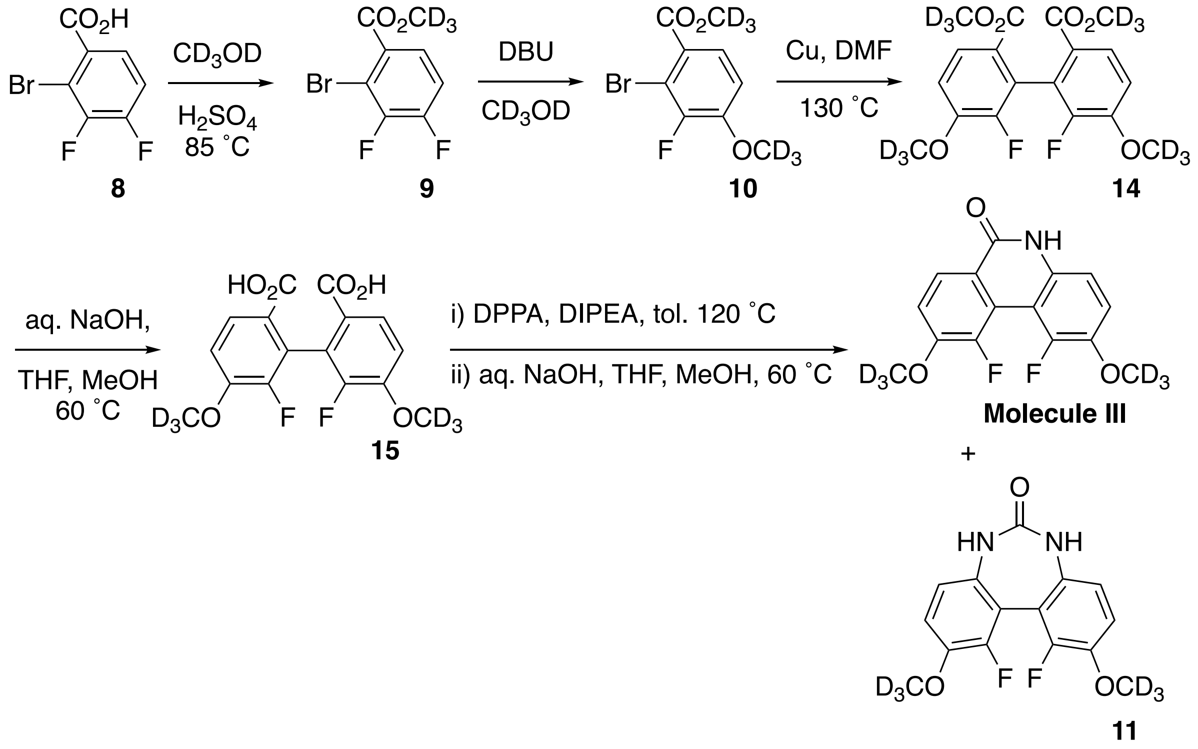

Scheme S3. Synthetic route to **Molecule III**.

Methyl-d_3 2-bromo-3,4-difluorobenzoate 9

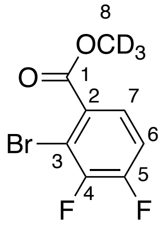

To a solution of 2-bromo-3,4-difluorobenzoic acid **8** (3.00 g, 12.66 mmol) in CD_3OD (6.0 mL), concentrated H_2SO_4 (0.25 mL) was added dropwise to the reaction at 0 °C. The reaction mixture was heated to 85 °C for 16 h. After cooling to room temperature, the reaction mixture was concentrated *in vacuo*. The residue was taken up in ethyl acetate (80 mL) and water (100 mL) was added. The organic layer was separated and washed sequentially with $NaHCO_3$ (80 mL), water (80 mL), brine (80 mL), dried over magnesium sulfate, filtered, and concentrated *in vacuo*. The residue was purified by flash column chromatography (ethyl acetate/petroleum ether, 1/4) to give *the title compound* ***9*** (3.00 g, 11.81 mmol, 93% yield) as a colourless liquid.

R_f 0.35 (ethyl acetate/petroleum ether, 1/4).

δ_H (500 MHz, $CDCl_3$) 7.66 (1H, ddd, *J* 8.8, 5.2, 2.2, H7), 7.20 (1H, ddd, *J* 8.9, 8.8, 7.2, H6).

δ_C (126 MHz, $CDCl_3$) 165.0 (C1), 152.8 (dd, *J* 258.5, 14.2, C5), 148.7 (dd, *J* 248.3, 14.2, C4), 128.9 (dd, *J* 3.8, 0.8, C2), 127.2 (dd, *J* 7.7, 4.3, C7), 166.0 (d, *J* 18.0, C6), 112.0 (dd, *J* 19.1, 0.8, C3), 52.1 (sept., *J* 22.5, C8).

δ_F {^{1}H} (471 MHz, $CDCl_3$) –125.10 (d, *J* 21.9), –128.17 (d, *J* 21.9)

δ_F (471 MHz, $CDCl_3$) –125.10 (ddd, *J* 21.9, 7.3, 2.1), –128.17 (ddd, *J* 21.9, 8.9, 5.3).

δ_D (77 MHz, $CHCl_3$) 3.91 (3D, br, OCD_3).

ν_{max} 3098, 3042, 2958, 1928, 1724, 1598, 1488, 1414, 1284, 1261, 1199, 1123, 999, 909, 841, 769.

HRMS (ESI) found $[M+H]^+$ 253.9704, $C_8H_3D_3{}^{79}BrF_2O_2$ requires 253.9702.

Methyl-d_3 2-bromo-3-fluoro-4-(methoxy-d_3) benzoate 10

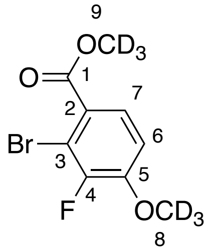

Based on a patent procedure,[8] to a solution of methyl-d_3 2-bromo-3,4-difluorobenzoate **9** (3.00 g, 11.81 mmol) in CD_3OD (10.0 mL), 1,8-diazabicyclo[5.4.0]undec-7-ene (DBU, 2.65 mL, 17.81 mmol) was added at room temperature, and the reaction mixture was stirred at 70 °C for 3 h. A solution of $NaHCO_3$ (100 mL) was added, and the mixture was extracted with ethyl acetate (80 mL). The combined organic layers were washed with brine (80 mL), dried over magnesium sulfate, and concentrated *in vacuo*. The residue was purified by flash column chromatography (ethyl acetate/petroleum ether, 1/4) to give *the title compound* ***10*** (2.60 g, 9.77 mmol, 83% yield) as a white solid.

R_f 0.27 (ethyl acetate/petroleum ether, 1/4).

δ_H (500 MHz, $CDCl_3$) 7.68 (1H, dd, *J* 8.8, 2.0, H7), 6.90 (1H, t, *J* 8.3, H6).

δ_C (126 MHz, $CDCl_3$) 165.4 (C1), 151.3 (d, *J* 12.3, C5), 149.7 (d, *J* 245.6, C4), 127.6 (d, *J* 4.1, C7), 124.0 (C2), 111.1-110.9 (m, C6, C3), 55.8 (sept., *J* 22.2, C8), 51.7 (sept., *J* 22.5, C9).

δ_F {^{1}H} (471 MHz, $CDCl_3$) –123.2.

δ_F (471 MHz, $CDCl_3$) –123.2 (dd, *J* 7.7, 2.0).

δ_D (77 MHz, $CHCl_3$) 3.91 (3D, br, CO_2CD_3), 3.89 (3D, br, $COCD_3$).

ν_{max} 3100, 2921, 2616, 2268, 2242, 2186, 2123, 2079, 1716, 1601, 1490, 1425, 1298, 1273, 1211, 1161, 1103, 1084, 1010, 997, 958, 866, 809, 763.

HRMS (ESI) found $[M+H]^+$ 269.0088, $C_9H_3D_6{}^{79}BrFO_3$ requires 269.0090.

MP 118-120.

Bis(methyl-*d₃*) 6,6'-difluoro-5,5'-bis(methoxy-*d₃*)-[1,1'-biphenyl]-2,2'-dicarboxylate 14

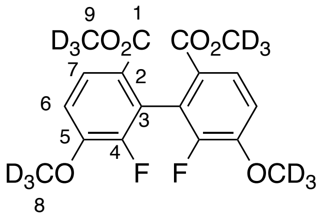

A solution of methyl-*d₃* 2-bromo-3-fluoro-4-(methoxy-*d₃*) benzoate **10** (2.50 g, 9.29 mmol) and copper(0) powder (4.72 g, 74.34 mmol) in *N,N*-dimethylformamide (10 mL) was heated at 130 ˚C for 4 h. After the reaction was cooled to room temperature, the solids were removed by filtration. Water (100 mL) was added to the eluent, and it was extracted with dichloromethane (80 mL). The organic layer was washed with brine (80 mL), dried over magnesium sulfate, filtered, and concentrated *in vacuo*. The residue was purified by flash column chromatography (ethyl acetate/petroleum ether, 4/6) to give *the title compound* ***14*** (1.55 g, 4.1 mmol, 88% yield) as a white solid.

R_f 0.33 (ethyl acetate/petroleum ether, 4/6).

δ_H (500 MHz, $CDCl_3$) 7.92 (2H, dd, *J* 8.8, 1.6, H7), 7.02 (2H, t, *J* 8.3, H6).

δ_C (126 MHz, $CDCl_3$) 165.7 (C1), 151.0 (d, *J* 11.6, C5), 149.1 (d, *J* 243.8, C4), 127.1 (dd, *J* 14.4, 3.5, C7), 125.0 (d, *J* 16.7, C3), 122.2 (C2), 111.6 (d, *J* 3.3, C6), 55.8 (sept., *J* 22.0, C8), 51.7 (sept., *J* 22.6, C9).

δ_F {^{1}H} (471 MHz, $CDCl_3$) –134.9.

δ_F (471 MHz, $CDCl_3$) –134.9 (d, *J* 8.5).

δ_D (77 MHz, $CHCl_3$) 3.88 (3D, br, CO_2CD_3), 3.56 (3D, br, $COCD_3$).

ν_{max} 2242, 2081, 1710, 1612, 1565, 1502, 1485, 1430, 1279, 1205, 1165, 1152, 1105, 1084, 996, 900, 864, 767, 722, 662.

HRMS (ESI) found $[M+Na]^+$ 401.1559, $C_{18}H_4D_{12}F_2NaO_6$ requires 401.1560.

MP 164-166.

6,6'-Difluoro-5,5'-bis(methoxy-*d*$_3$)-[1,1'-biphenyl]-2,2'-dicarboxylic acid 15

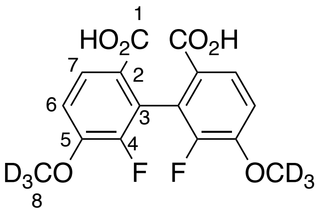

To a solution of bis(methyl-*d*$_3$) 6,6'-difluoro-5,5'-bis(methoxy-*d*$_3$)-[1,1'-biphenyl]-2,2'-dicarboxylate **14** (1.50 g, 3.96 mmol) in THF/MeOH (2:1, 16 mL), 2 *N* aqueous NaOH (5 mL) was added at room temperature. After stirring at 60 °C for 16 h, the reaction mixture was washed with diethyl ether (60 mL). The aqueous layer was acidified with 2 *N* aqueous HCl until pH2 was attained, followed by extraction with ethyl acetate (60 mL). The organic layer was washed with brine (60 mL), dried over magnesium sulfate, filtered, and concentrated *in vacuo* to give *the title compound **15*** (1.23 g, 3.57 mmol, 90% yield) as a white solid.

R_f 0.28 (ethyl acetate/petroleum ether, 4/1).

δ_H (500 MHz, CD_3OD) 7.93 (2H, dd, *J* 8.8, 1.5, H7), 7.16 (2H, t, *J* 8.1, H6).

δ_C (126 MHz, CD_3OD) 168.6 (C1), 152.4 (d, *J* 11.8, C5), 150.3 (d, *J* 242.3, C4), 128.4 (d, *J* 3.5, C7), 126.4 (d, *J* 16.8, C3), 123.8 (C2), 112.6 (d, *J* 1.5, C6), 55.8 (sept., *J* 22.1, C8).

δ_F {^{1}H} (471 MHz, CD_3OD) –137.1.

δ_F (471 MHz, CD_3OD) –137.1 (d, *J* 8.5).

δ_D (77 MHz, CH_3OH) 4.66 (6D, br, OCD_3).

ν_{max} 2821, 2562, 2078, 1672, 1603, 1567, 1470, 1428, 1275, 1210, 1147, 1098, 992, 945, 826, 777, 724, 676.

HRMS (ESI) found $[M+H]^-$ 343.0907, $C_{16}H_5D_6F_2O_6$ requires 343.0906.

MP 268-270.

1,10-Difluoro-2,9-bis(methoxy-d_3) phenanthridin-6(5*H*)-one 'Molecule III'

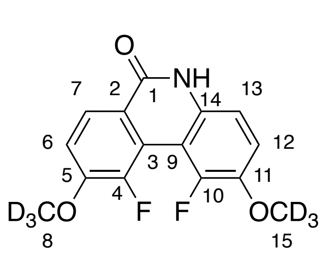

major compound
Molecule III

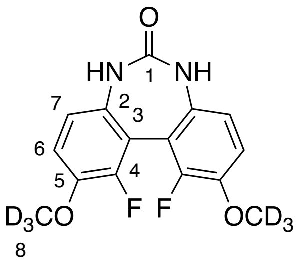

minor compound
11

To a solution of 6,6'-difluoro-5,5'-bis(methoxy-d_3)-[1,1'-biphenyl]-2,2'-dicarboxylic acid **15** (1.20 g, 3.47 mmol) in toluene (50 mL), diphenylphosphoryl azide (DPPA, 1.49 mL, 6.92 mmol) and *N*,*N*-diisopropylethylamine (3.62 mL, 20.76 mmol) were added at room temperature. After heating at 120 °C for 16 h, the reaction mixture was concentrated *in vacuo*. A solution of the resulting residue in THF/MeOH/2 *N* aqueous NaOH (1:1:1, 200 mL) was then stirred at 60 °C for 4 h, and the reaction mixture was extracted with ethyl acetate (60 mL). The organic layer was separated, washed with water (80 mL) and brine (80 mL), dried over magnesium sulfate, filtered, and concentrated *in vacuo*. The residue was purified by flash column chromatography (ethyl acetate/petroleum ether 1/1 to 100% ethyl acetate) to give *the title compound* ***'Molecule III'*** (0.82 g, 2.76 mmol, 80% yield) as a white solid. N.B. **Molecule III** was preceded by elution of a trace amount of **Molecule III** and 1,10-difluoro-2,9-bis(methoxy-*d*3)phenanthridin-6(5*H*)-one **11** as a mixture, from which diffraction quality single crystals of **11** were grown by vapour diffusion of dichloromethane into a saturated solution of hexafluoro-2-propanol.

Molecule III

R_f 0.32 (ethyl acetate/petroleum ether, 1:1).

δ_H (500 MHz, $(CD_3)_2SO$) 11.51 (1H, s, NH), 8.17 (1H, dd, *J* 8.8, 1.4, H7), 7.55 (1H, dd, *J* 8.8, 7.4, H6), 7.42 (1H, t, *J* 8.6, H12), 7.12 (1H, dd, *J* 9.0, 1.5, H13).

δ_C (126 MHz, $(CD_3)_2SO$) 159.6 (d, *J* 2.2, C1), 151.7 (d, *J* 11.6, C5), 148.3 (dd, *J* 245.3, 8.6, C10), 146.8 (dd, *J* 249.2, 6.0, C4), 143.1 (d, *J* 11.8, C11), 131.6 (d, *J* 5.5, C14), 124.6 (d, *J* 3.3, C7), 120.1 (d, *J* 2.1, C2), 119.3 (dd, *J* 11.6, 2.1, C3), 116.8 (d, *J* 2.1, C12), 114.2 (C6), 111.0 (d, *J* 3.8, C13), 104.4 (dd, *J* 15.0, 2.4, C9), 56.7-55.6 (m, C8, C15).

δ_F {^{1}H} (471 MHz, $(CD_3)_2SO$) –124.1 (d, *J* 175.7), –125.1 (d, *J* 175.7).

δ_F (471 MHz, $(CD_3)_2SO$) –124.1 (dd, *J* 175.5, 8.2), –125.1 (dd, *J* 175.5, 7.4).

δ_D (77 MHz, $(CH_3)_2SO$) 4.82 (3D, br, OCD_3), 4.68 (3D, br, OCD_3).

ν_{max} 2878, 2235, 2076, 1720, 1664, 1605, 1513, 1464, 1423, 1376, 1309, 1292, 1250, 1194, 1145, 1100, 1059, 992, 957, 875.

HRMS (ESI) found $[M+H]^+$ 298.1148, $C_{15}H_6D_6F_2NO_3$ requires 298.1156.

MP 274-276.

1,10-Difluoro-2,9-bis(methoxy-*d*3)phenanthridin-6(5*H*)-one 11

δ_H (500 MHz, $(CD_3)_2SO$) 8.78 (2H, s, NH), 7.23 (2H, m, H7), 6.96 (2H, d, H6).

δ_C (126 MHz, $(CD_3)_2SO$) 155.3 (C1), 148.0 (dd, *J* 261.1, 12.6, C4), 143.8 (m, C5), 135.1 (C2), 116.6 (C7), 114.8 (C6), 113.2 (C3), 56.5 – 55.4 (m, C8).

δ_F $\{^1H\}$ (471 MHz, $(CD_3)_2SO$) –131.70 (s).

δ_F (471 MHz, $(CD_3)_2SO$) –131.70 (m).

3. X-Ray Crystallography

1,10-Difluoro-2,9-bis(methoxy-d_3) phenanthridin-6(5H)-one 'Molecule III'

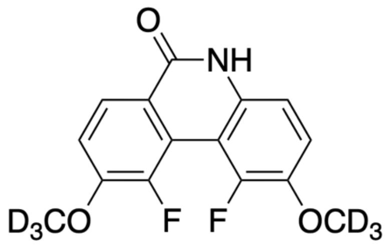

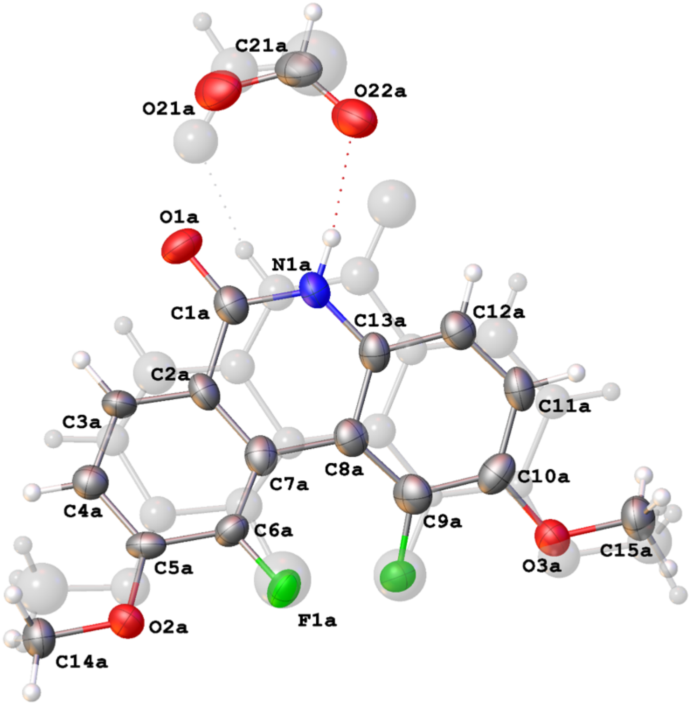

Experimental. Single colourless needle-shaped crystals of **Molecule III** were recrystallized by slow evaporation of 5% formic acid in 1,2-difluorobenzene. A suitable crystal with dimensions 0.20 × 0.02 × 0.01 mm^3 was selected and data were collected as described above (1.4 Crystallography).

The structure was solved and the space group $Pca2_1$ (# 29) determined by the ShelXT 2018/2[1,2] structure solution program using dual methods and refined by full matrix least squares minimisation on $\boldsymbol{F}^2$ using version of olex2.refine 1.5-dev.[4] All non-hydrogen atoms of the major disorder component were refined anisotropically (see below). Hydrogen atom positions were calculated geometrically and refined using the riding model.

The structure was refined as an inversion twin (BASF = 0.238) and exhibits whole molecule disorder. The occupancies of the disorder components were refined to ca.75:25 before being fixed at these values. Due to poor data to parameter ratio, the minor disorder component was refined isotropically. 1,2 and 1,3 equal distance restraints (SADI) with standard value ESDs were applied between chemically equivalent atom pairs of each disorder component. Thermal restraints (RIGU) with standard value ESDs were applied to all anisotropic non-H atoms.

Crystal Data. $C_{16}H_6D_6F_2NO_5$, M_r = 336.274, orthorhombic, $Pca2_1$ (No. 29), a = 22.2710(8) Å, b = 3.7377(2) Å, c = 16.8973(7) Å, $a = b = g = 90^{\circ}$, V = 1406.57(11) Å^3, T = 100(2) K, Z = 4, Z' = 1, m(Cu

K_a) = 1.176, 14080 reflections measured, 2662 unique (R_{int} = 0.0452) which were used in all calculations. The final wR_2 was 0.1301 (all data) and R_1 was 0.0484 ($I \geq 2\ s(I)$).

Selected measurements

F1a······F2a: 2.455(6) Å

F2b······F1b: 2.29(3) Å

F1a-C6a-C9a-F2a torsion angle: 46.5(6)°

F2b-C9b-C6b-F1b torsion angle: 30(2)°

Discussion

Due to the asymmetry of Molecule **III** and the puckering along the plane bisecting the middle ring, it exists in two subtly different conformations related by reflection in the crystal structure (see figure below)

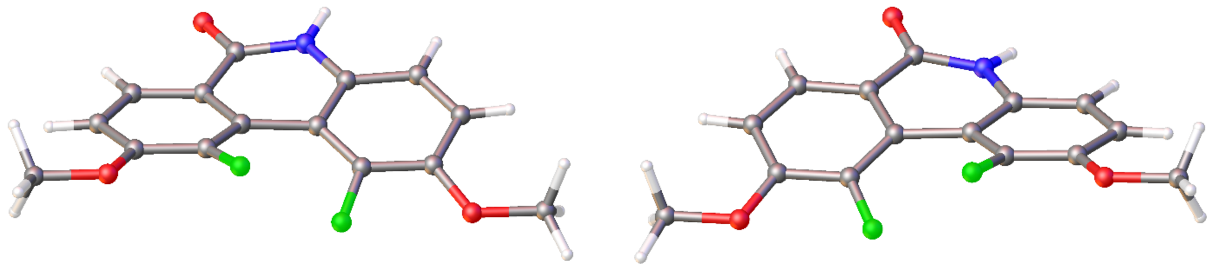

These two conformations are related by crystallographic symmetry and are present in a 1:1 ratio in the solid-state structure. This is not related to the 3:1 major:minor conformation ratio described previously which simply differ in the degree of puckering (both of these conformations and their reflected forms are present in 1:1 ratio). From the space-filling diagram shown below, it is not expected that the energy barrier to swap conformations is high, so the molecule is expected to be fluxional in solution at room temperature. Computational modelling might confirm this but is beyond the scope of this work.

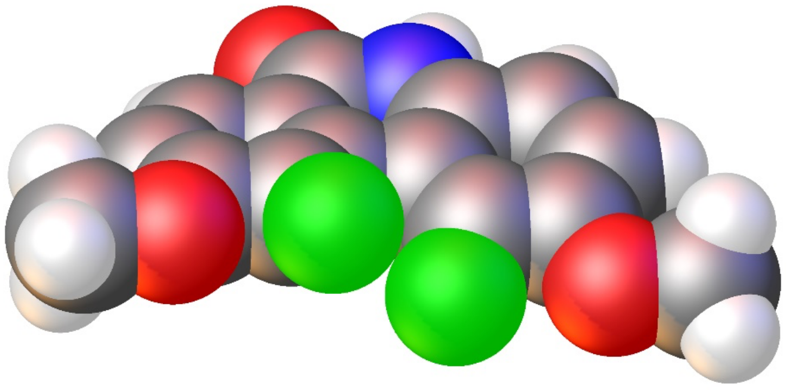

1,10-Difluoro-2,9-bis(methoxy-*d*3)phenanthridin-6(5*H*)-one 11

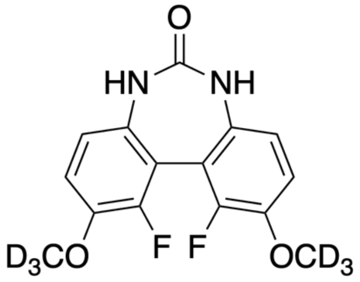

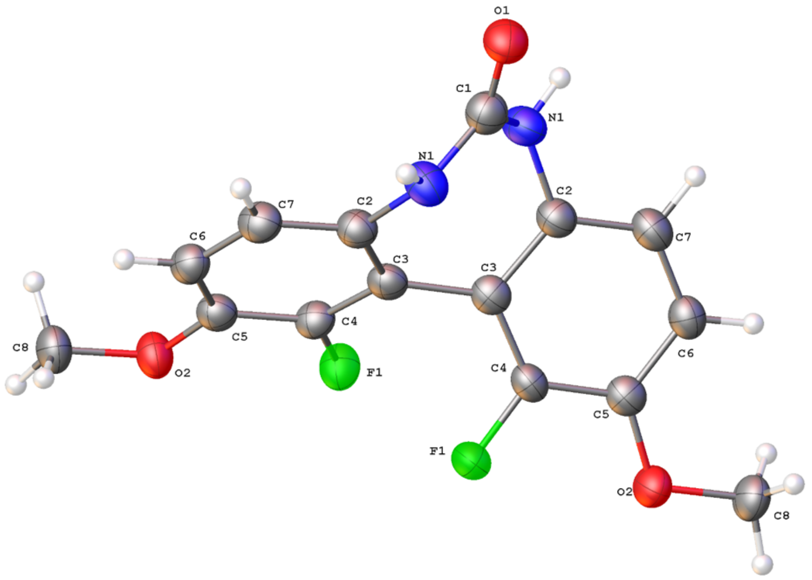

Experimental. Single colourless plate-shaped crystals of **11** were recrystallised by vapour diffusion of DCM into a saturated solution of hexafluoro-2-propanol. A suitable crystal 0.03×0.02×0.01 mm^3 was selected and data were collected as described above. The structure was solved in the space group *C*2/*c* (# 15) by using dual methods using the ShelXT 2018/2[1,2] structure solution program and refined by full matrix least squares minimisation on $\boldsymbol{F}^2$ using ShelXL 2018/3.[1,2] All non-hydrogen atoms were refined anisotropically. All hydrogen atom positions were calculated geometrically and refined using the riding model, except the amine H-atom which was located in the difference map and had a geometrical distance restraint (DFIX) applied and was refined with the riding model.

The value of Z' is 0.5. This means that only half of the formula unit is present in the asymmetric unit, with the other half consisting of symmetry equivalent atoms.

Crystal Data. $C_{15}H_6D_6F_2N_2O_3$, M_r = 312.30, monoclinic, *C*2/*c* (No. 15), a = 8.0810(5) Å, b = 12.6675(7) Å, c = 12.5057(10) Å, *b* = 103.715(7)°, *a* = *g* = 90°, *V* = 1243.66(15) Å^3, *T* = 100(2) K, *Z* = 4, *Z'* = 0.5, *m*(Cu K_a) = 1.161 mm^{-1}, 5706 reflections measured, 1165 unique (R_{int} = 0.0433) which were used in all calculations. The final wR_2 was 0.1217 (all data) and R_1 was 0.0456 (I≥2 *s*(I)).

4. References

1. CrysAlisPro Software System, Rigaku Oxford Diffraction, Poland, (2024).

2. Sheldrick, G.M., ShelXT-Integrated space-group and crystal-structure determination, *Acta Cryst.*, **2015**, *A71*, 3-8.

3. Sheldrick, G.M., Crystal structure refinement with ShelXL, *Acta Cryst.*, **2015**, *C71*, 3-8.

4. O.V. Dolomanov and L.J. Bourhis and R.J. Gildea and J.A.K. Howard and H. Puschmann, Olex2: A complete structure solution, refinement and analysis program, *J. Appl. Cryst.*, **2009**, *42*, 339-341.

5. L.J. Bourhis and O.V. Dolomanov and R.J. Gildea and J.A.K. Howard and H. Puschmann, The Anatomy of a Comprehensive Constrained, Restrained, Refinement Program for the Modern Computing Environment - Olex2 Disected, *Acta Cryst. A*, **2015**, *A71*, 59-71.

6. T. Murai, Y. Xing, M. Kurokawa, T. Kuribayashi, M. Nikaido, E. E. Elboray, S. Hamada, Y. Kobayashi, T. Sasamori, T. Kawabata and T. Furuta, *J. Org. Chem.*, **2022**, *87*, 5510–5521.

7. C. L. Perrin, Y. Dong, *J. Am. Chem. Soc.*, **2007**, *129*, 4490-4497.

8. Novartis AG, Basel (CH), WO 2023/031801, PCT/IB2022/058133.

5. NMR Spectra

Molecule I: 1,10-difluorophenanthridin-6(5*H*)-one

^{1}H, 500 MHz, $(CD_3)_2SO$

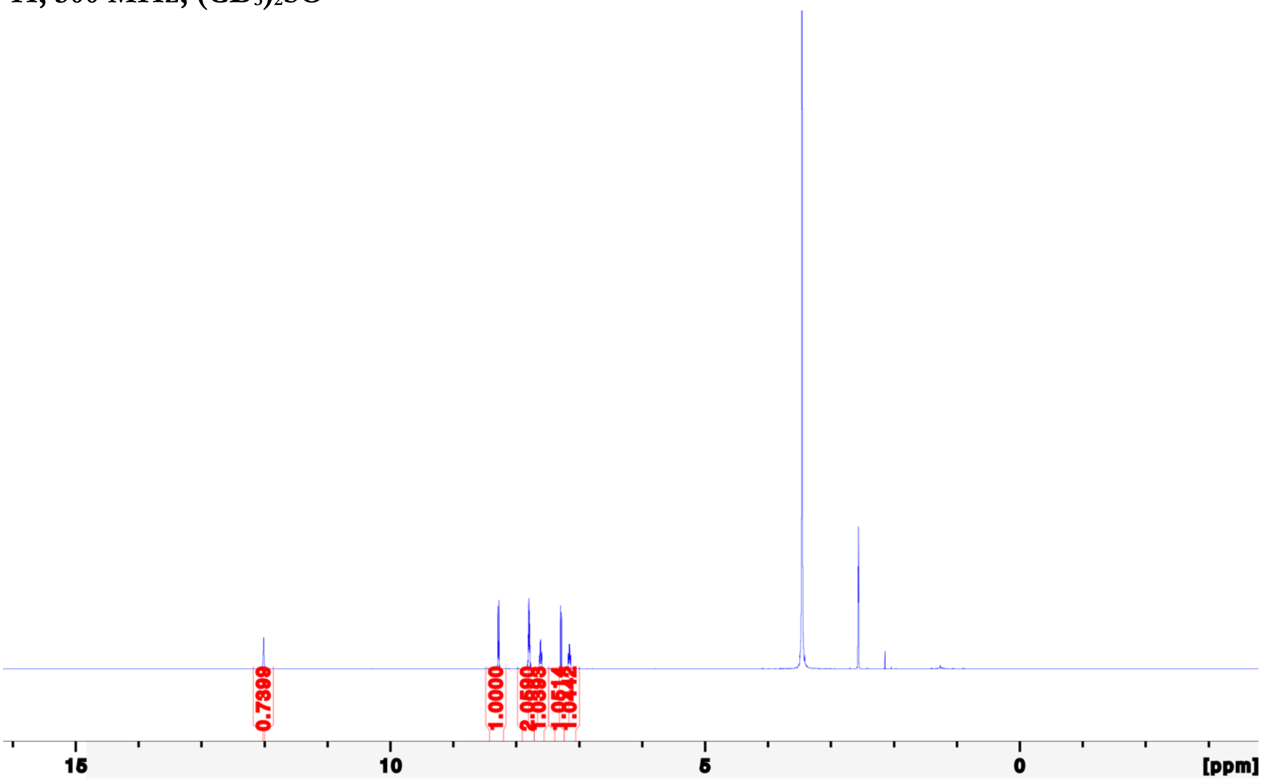

^{13}C, 126 MHz, $(CD_3)_2SO$

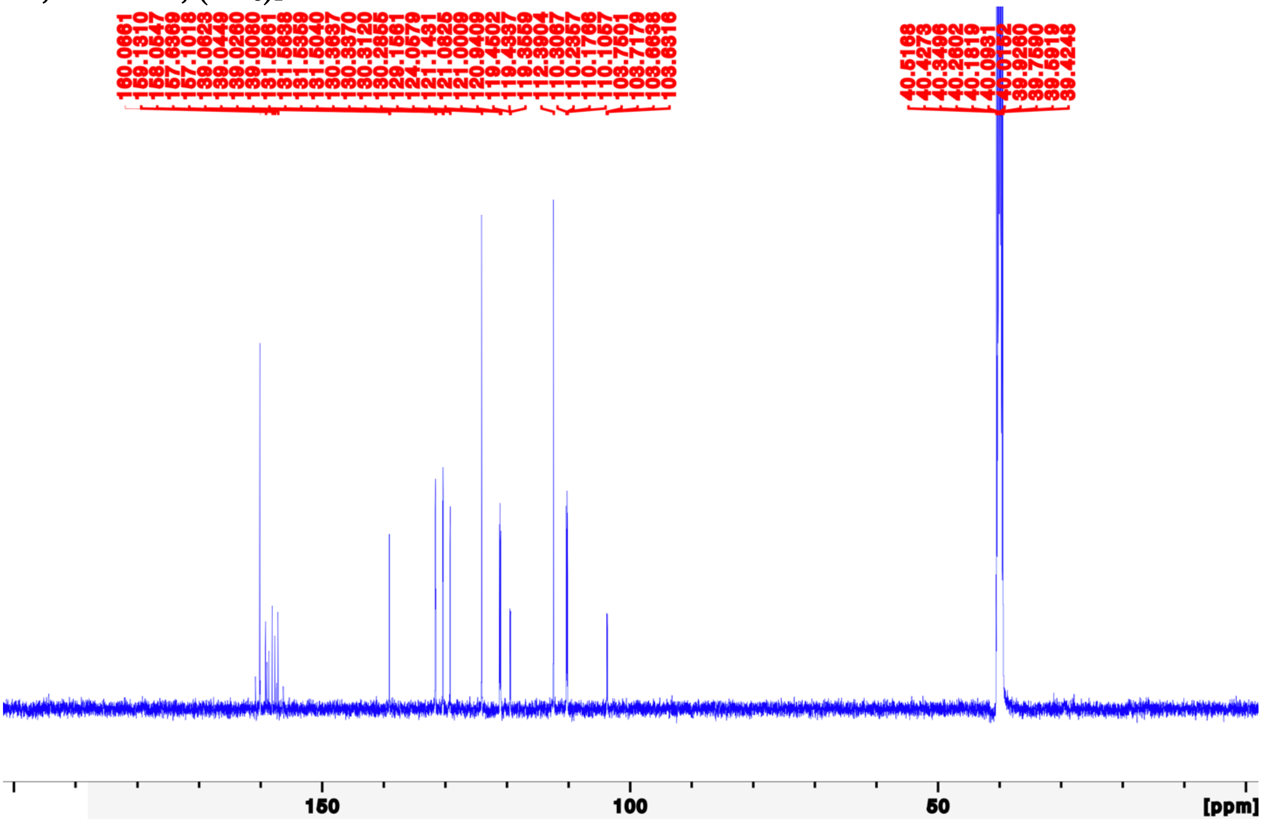

^{19}F, 471 MHz, $(CD_3)_2SO$

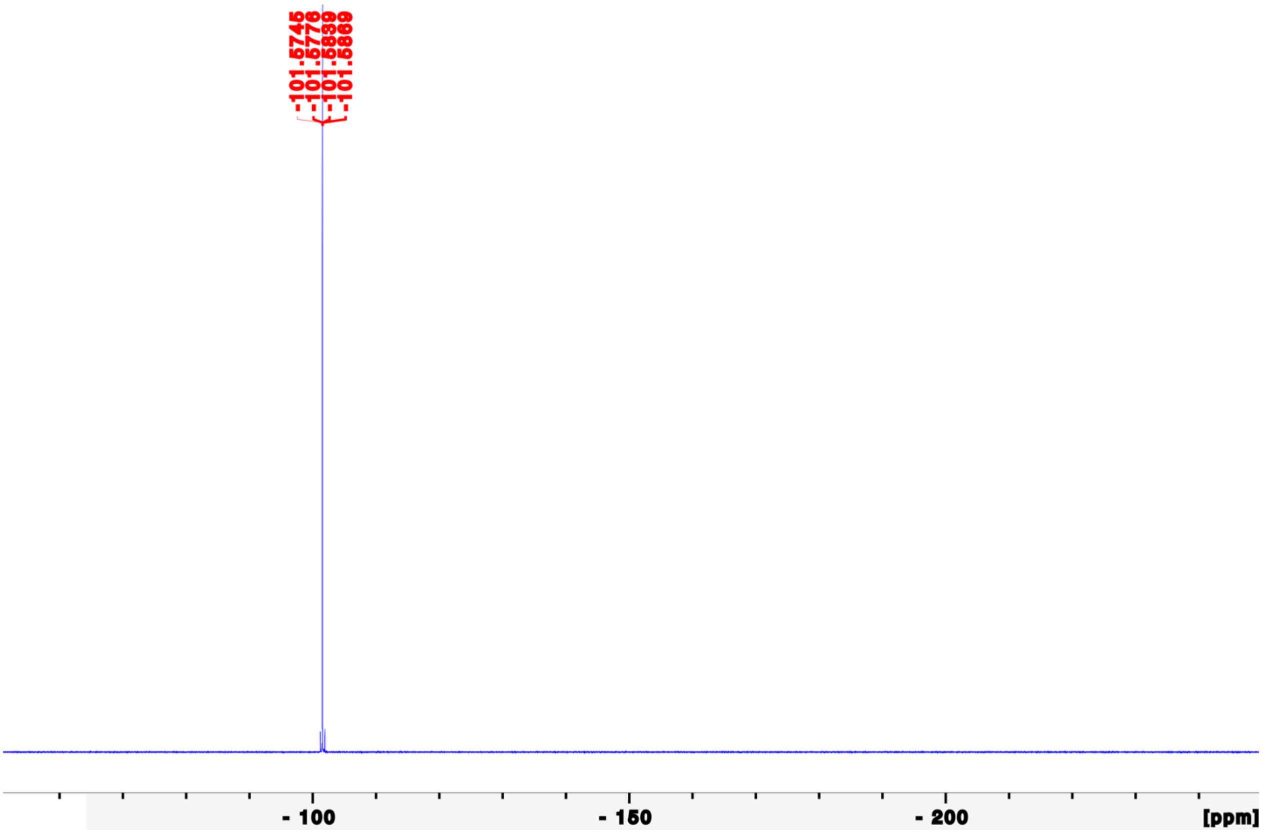

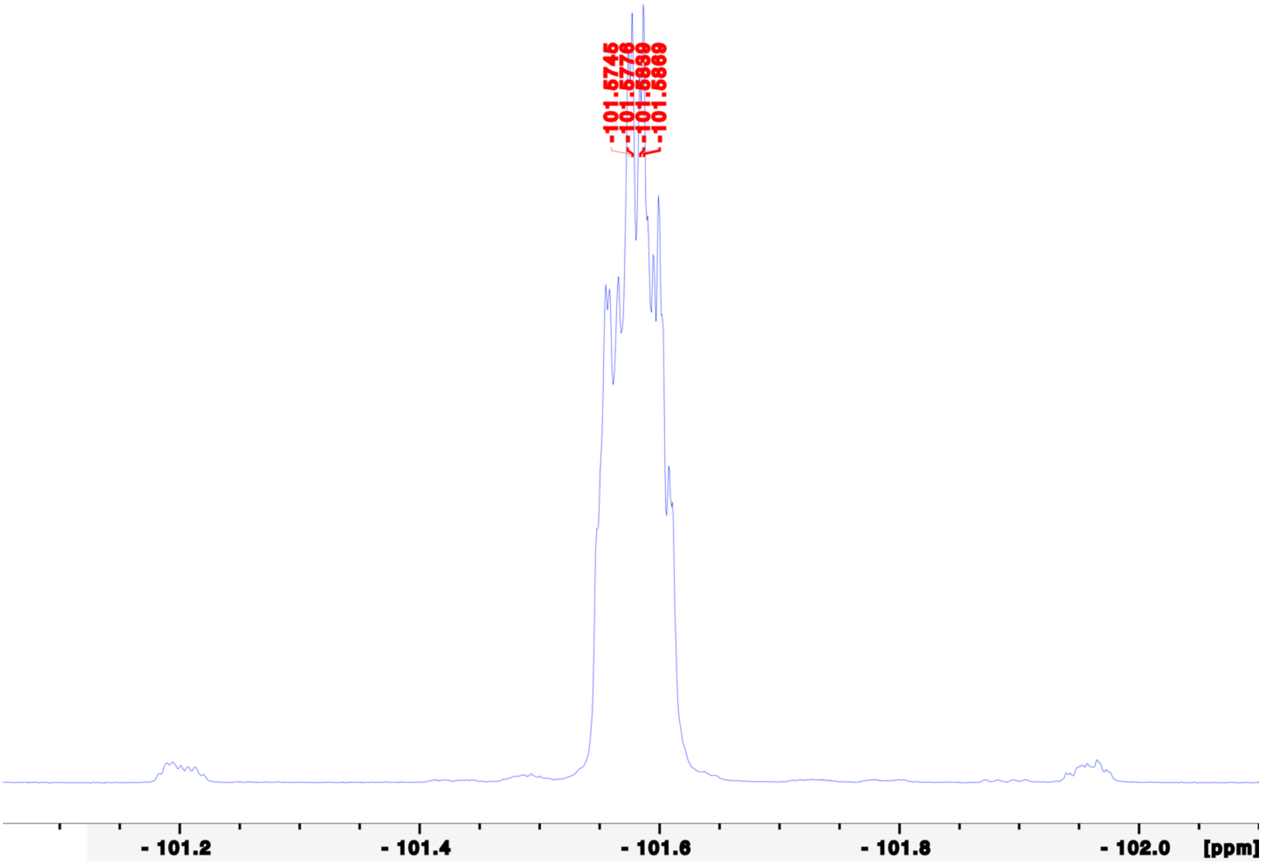

$^{19}F\ \{^1H\}$, 471 MHz, $(CD_3)_2SO$

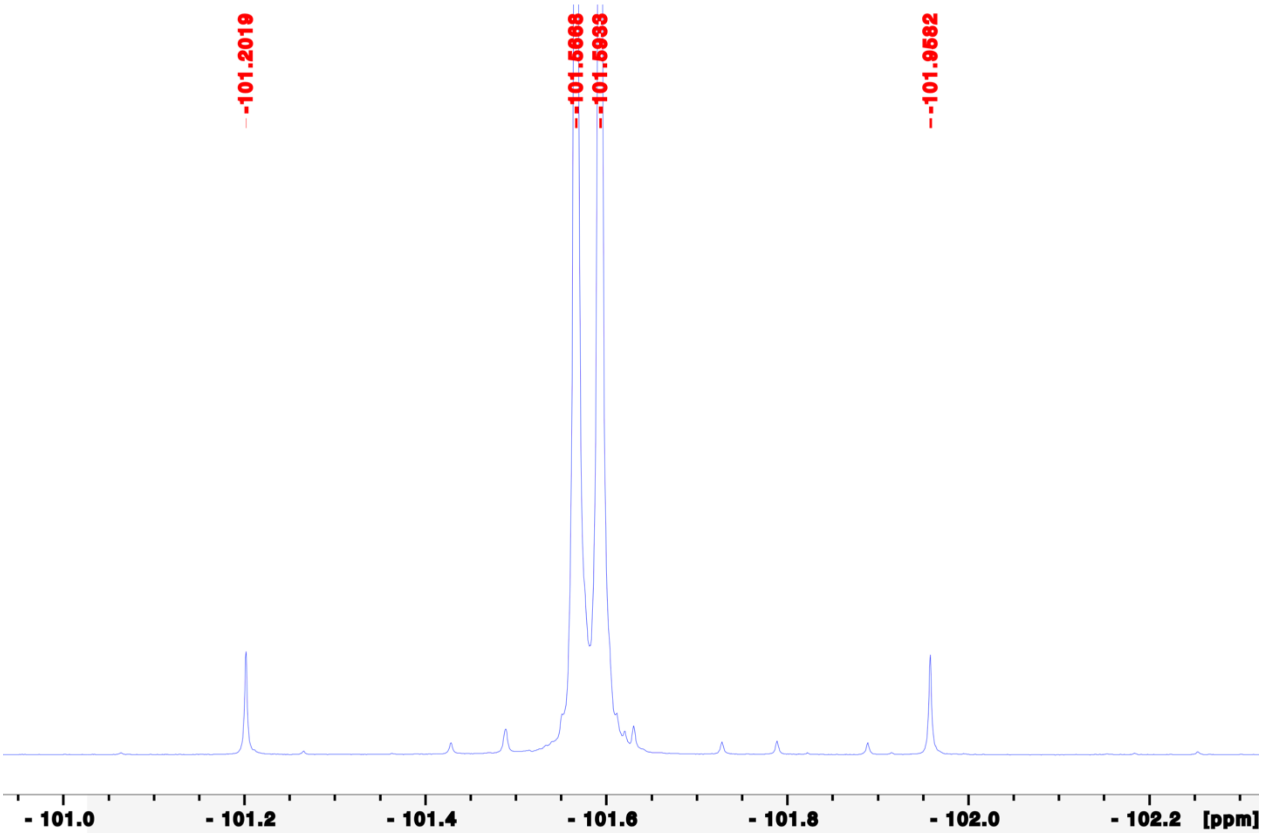

2-Bromo-3-fluorobenzoic-4,5,6-d_3 acid 6

^{1}H, 500 MHz, $CDCl_3$

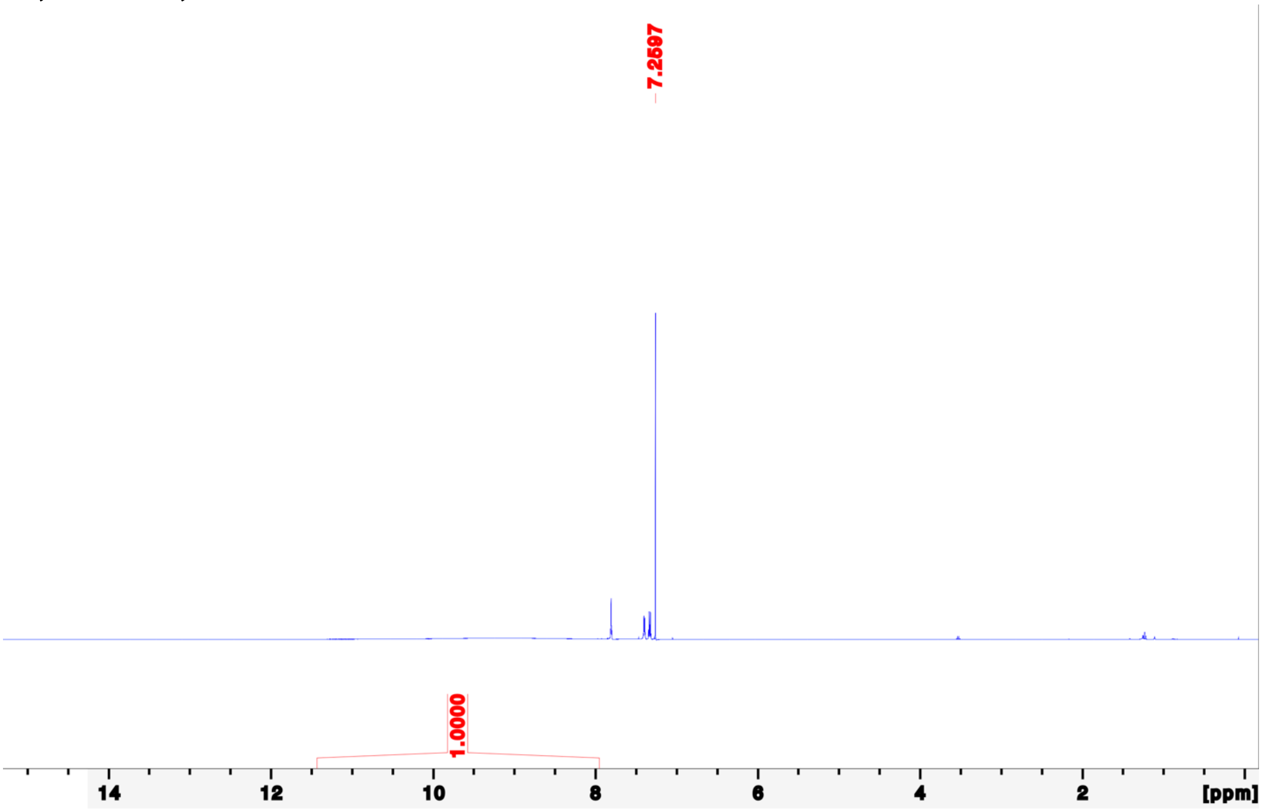

^{13}C, 126 MHz, $CDCl_3$

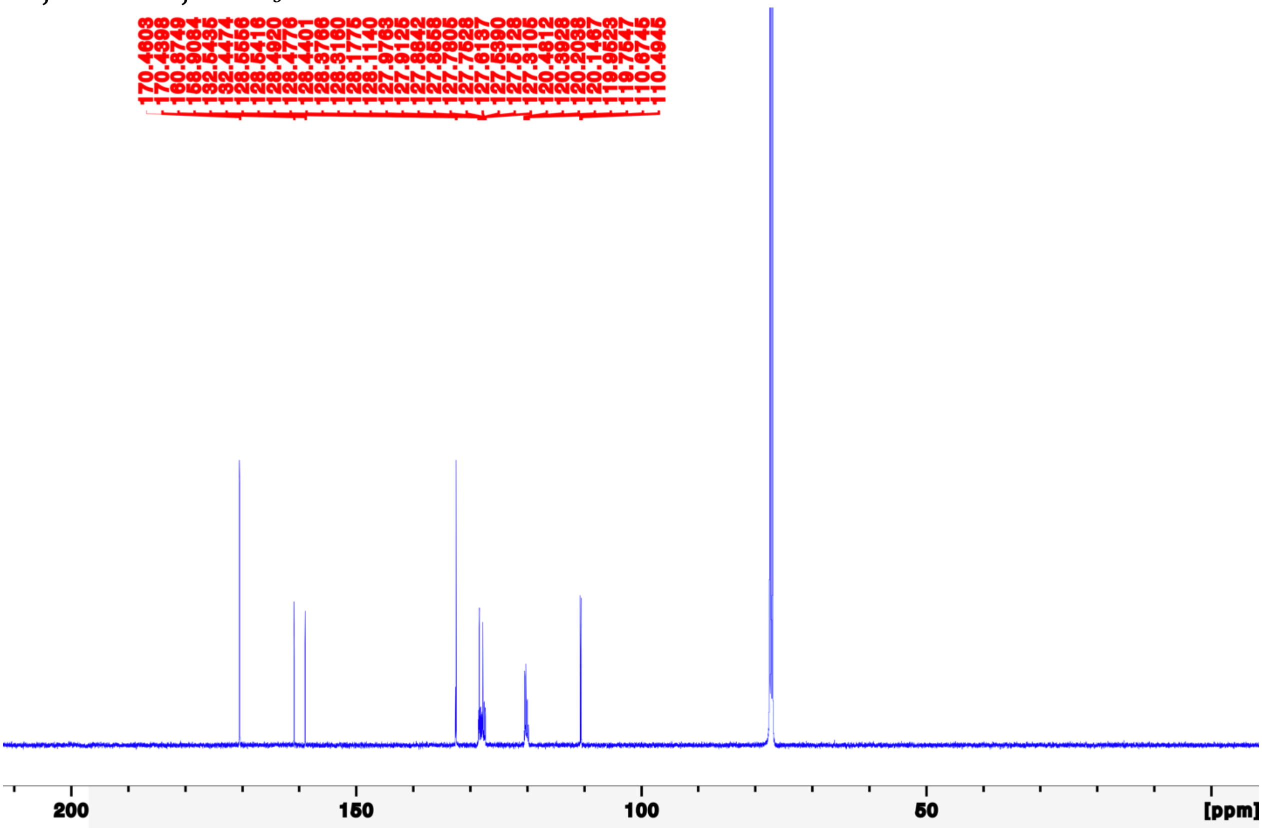

^{19}F, 471 MHz, $CDCl_3$

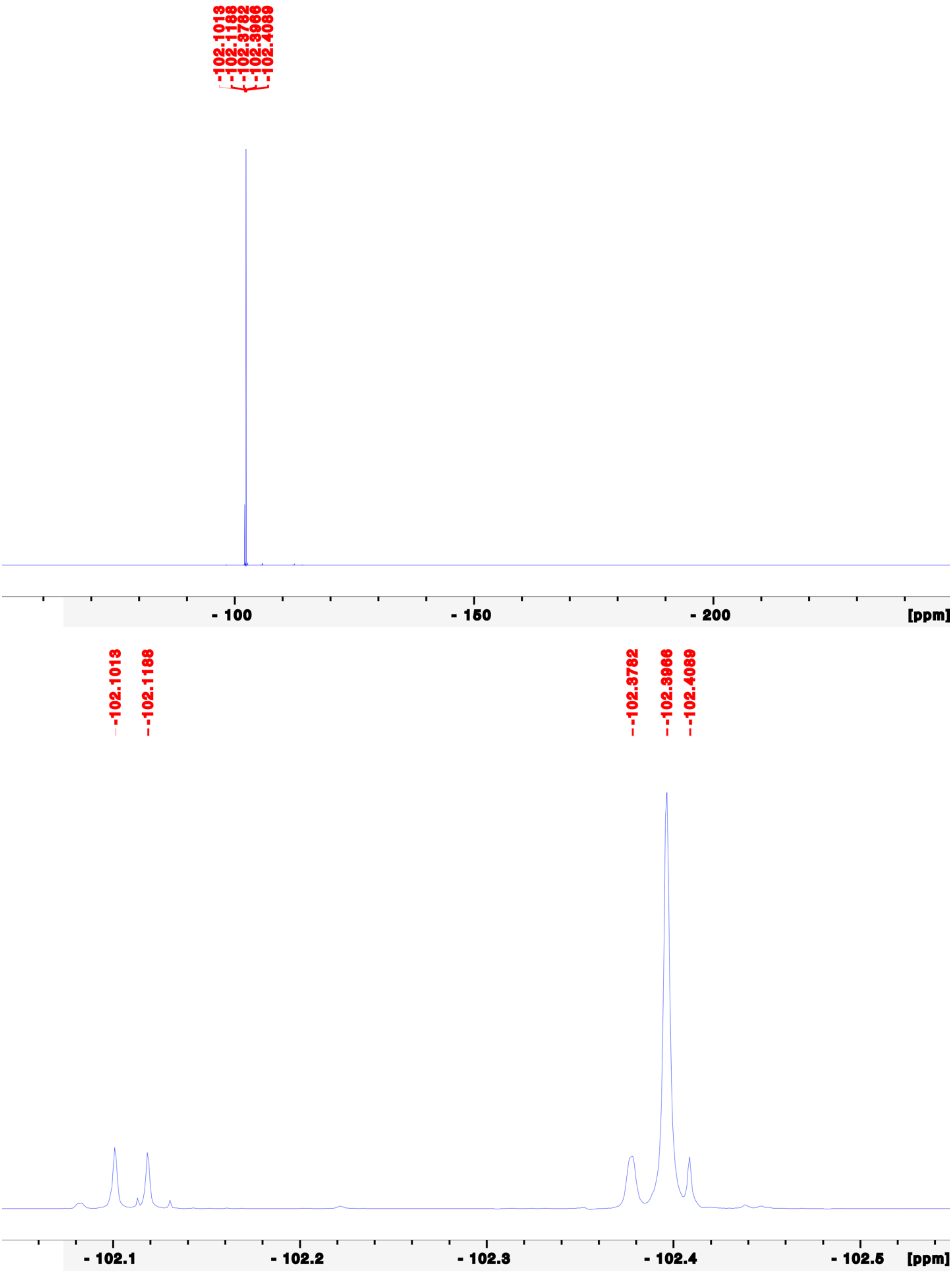

$^{19}F\{^{1}H\}$, 471 MHz, $CDCl_3$

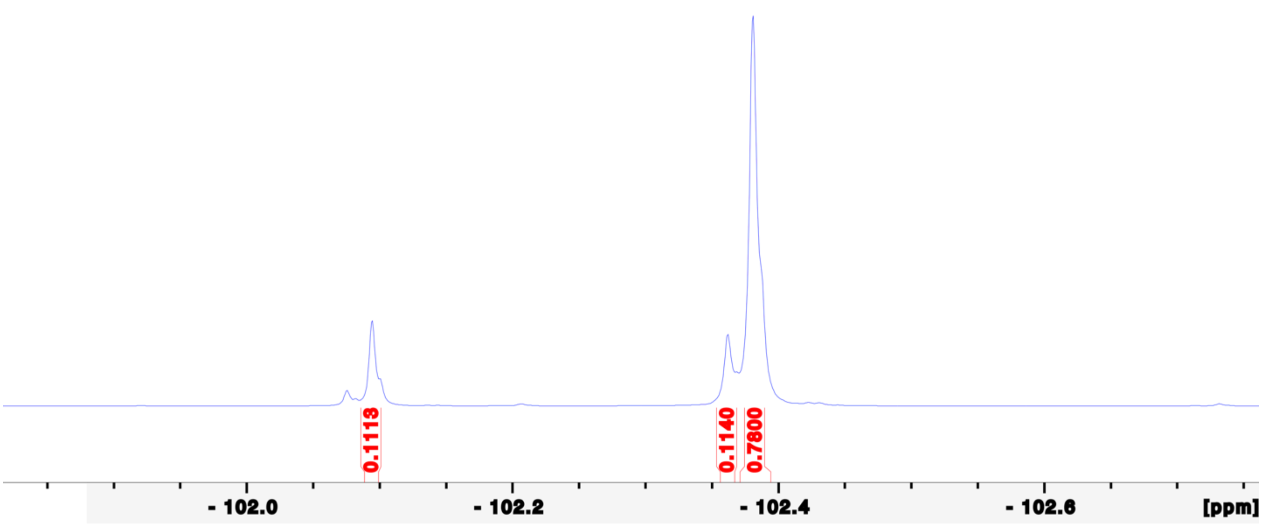

^{2}H, 77 MHz, $CHCl_3$

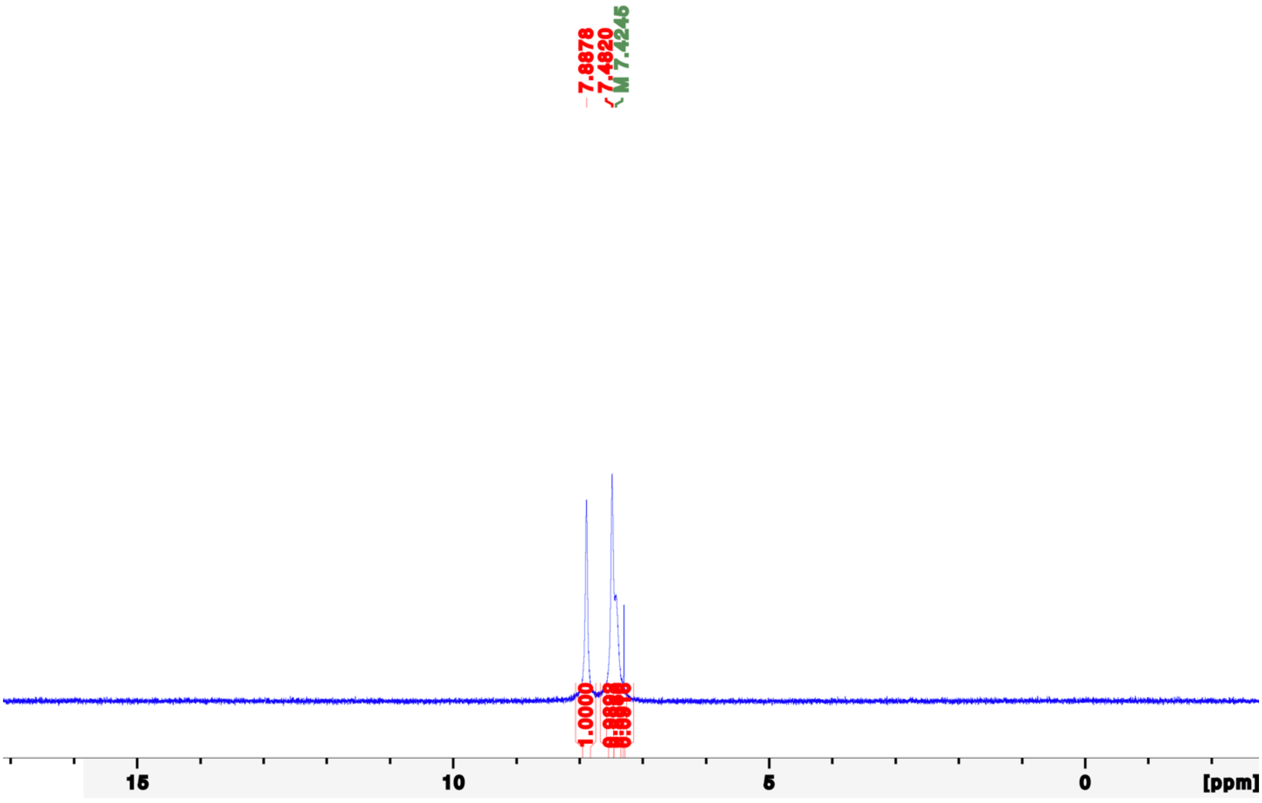

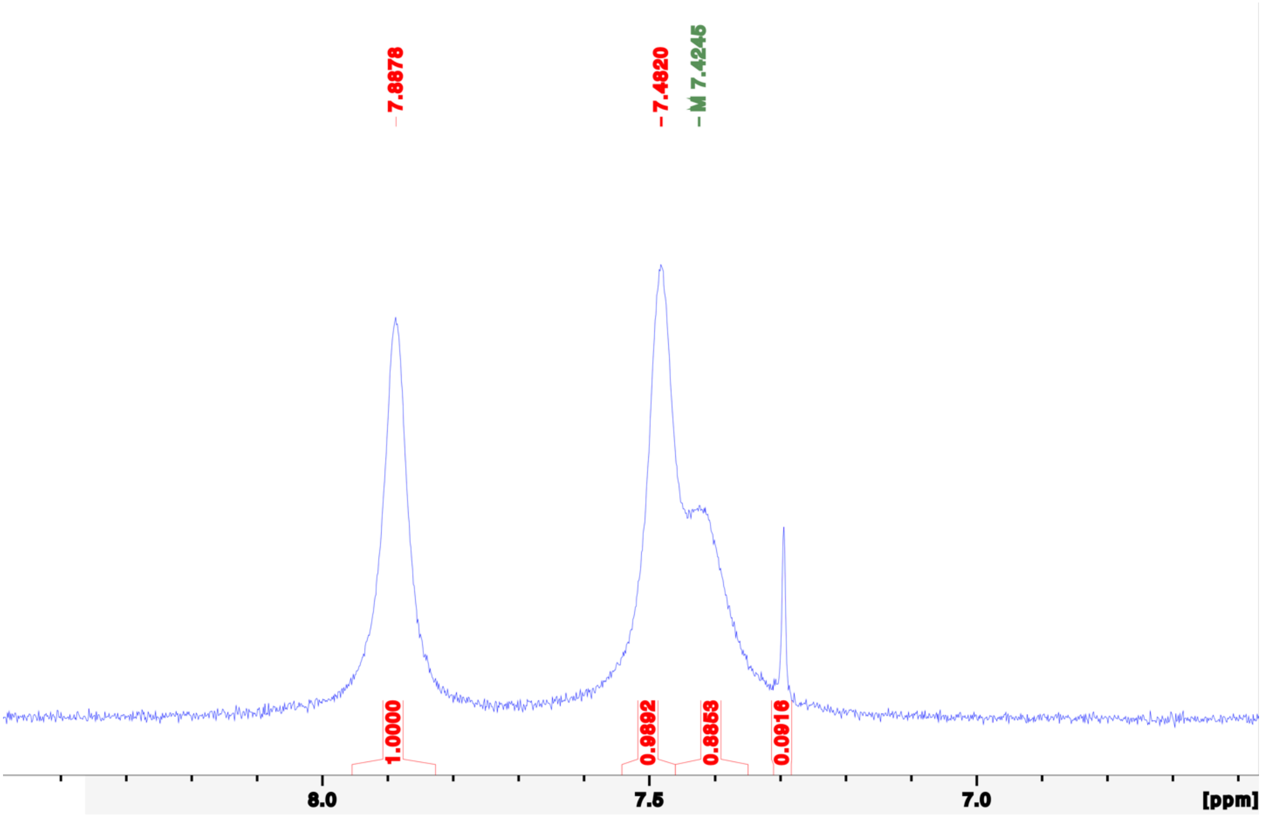
7.8878
7.4820
M 7.4245
1.0000
0.9892
0.8853
0.0916
8.0
7.5
7.0
[ppm]

Methyl 2-bromo-3-fluorobenzoate-4,5,6-d_3 12

^{1}H, 500 MHz, $CDCl_3$

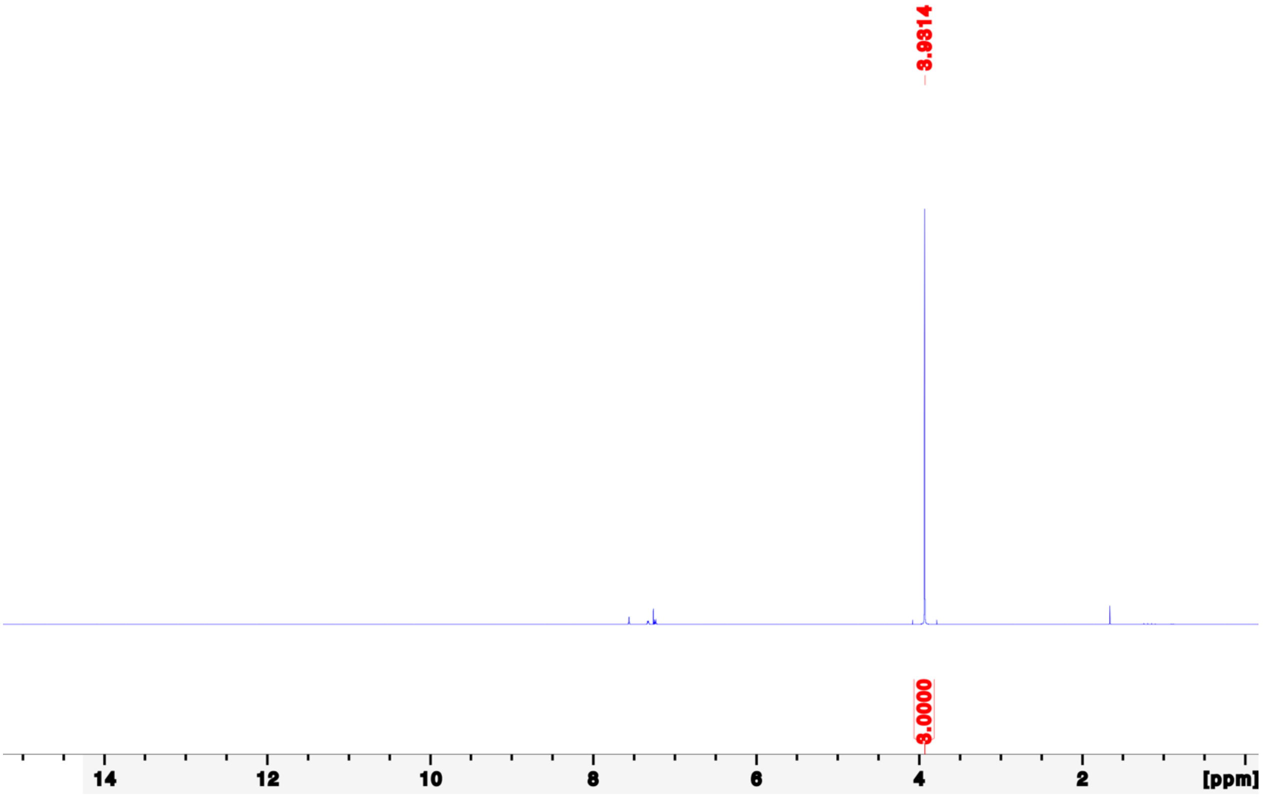

^{13}C, 126 MHz, $CDCl_3$

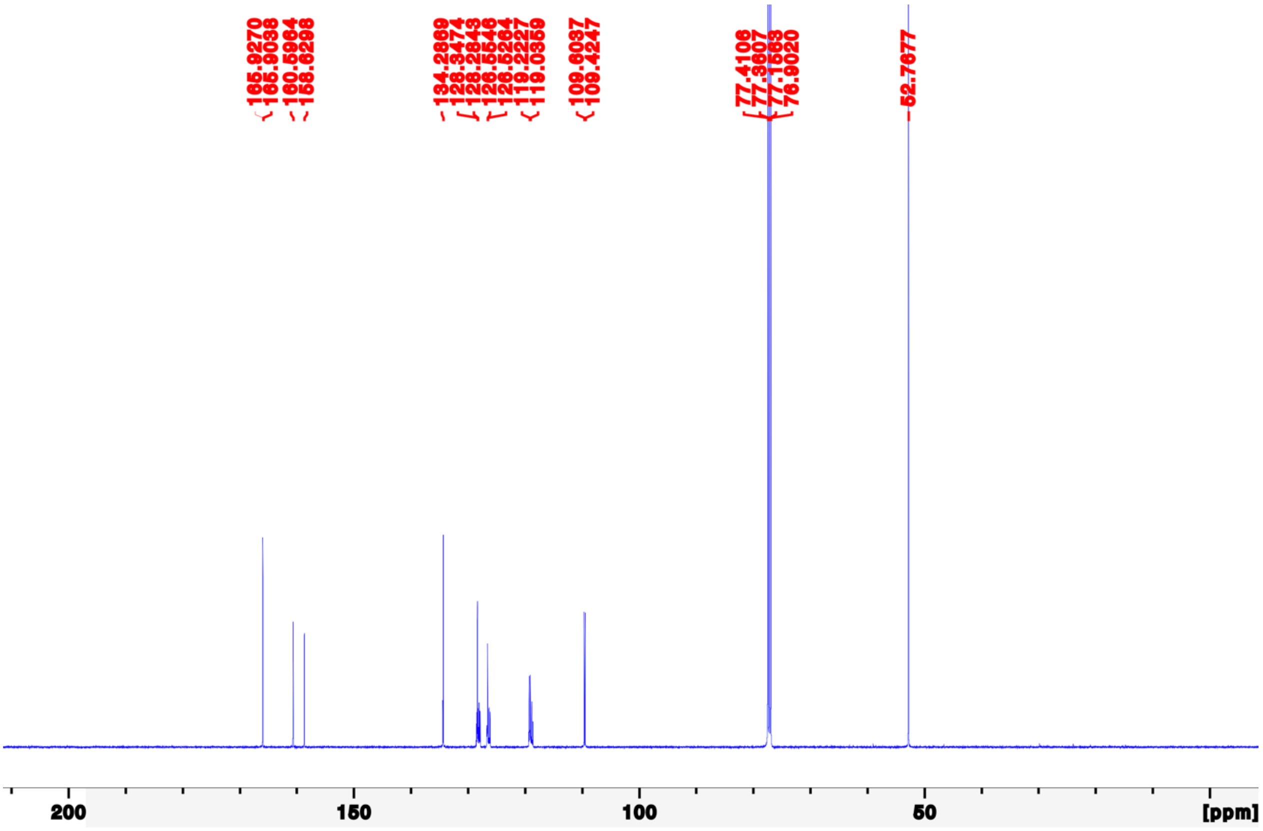

^{19}F, 471 MHz, $CDCl_3$

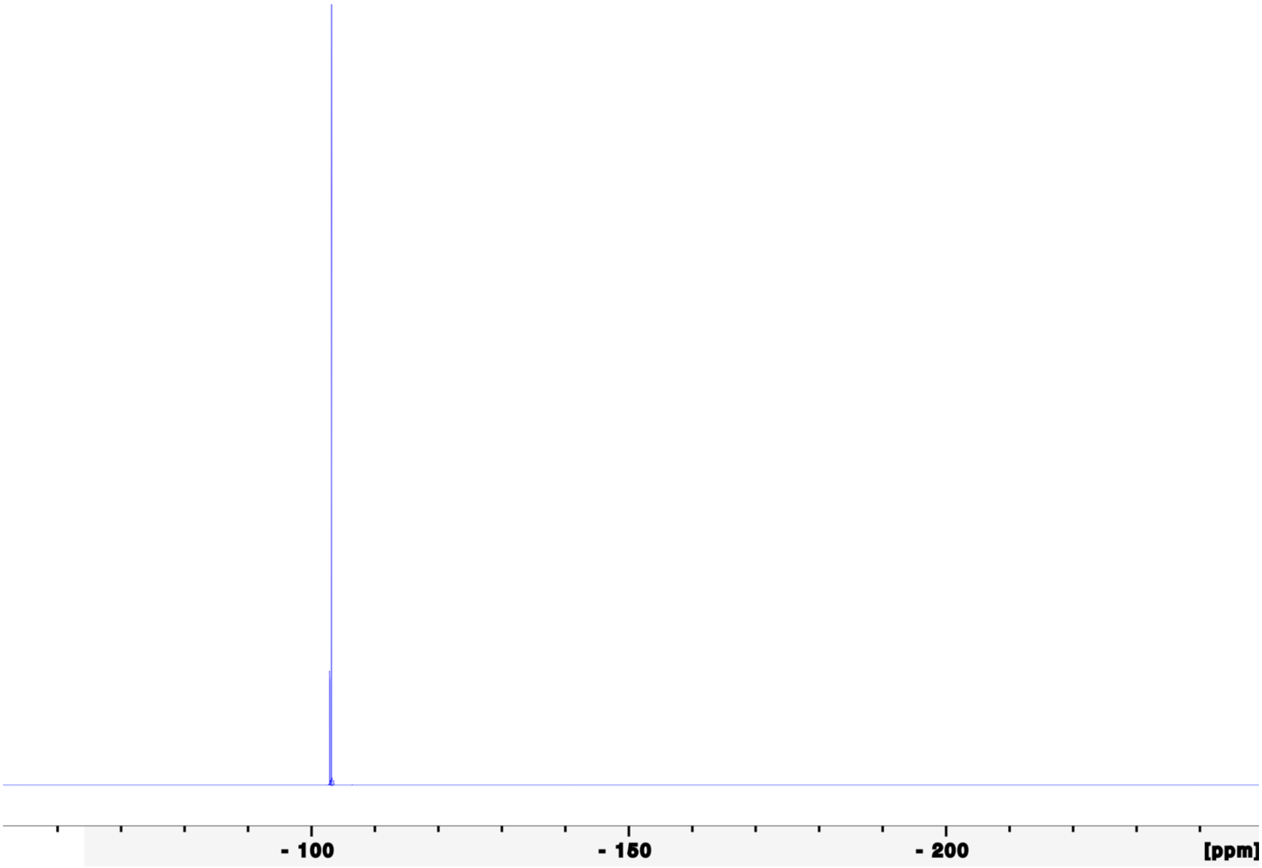

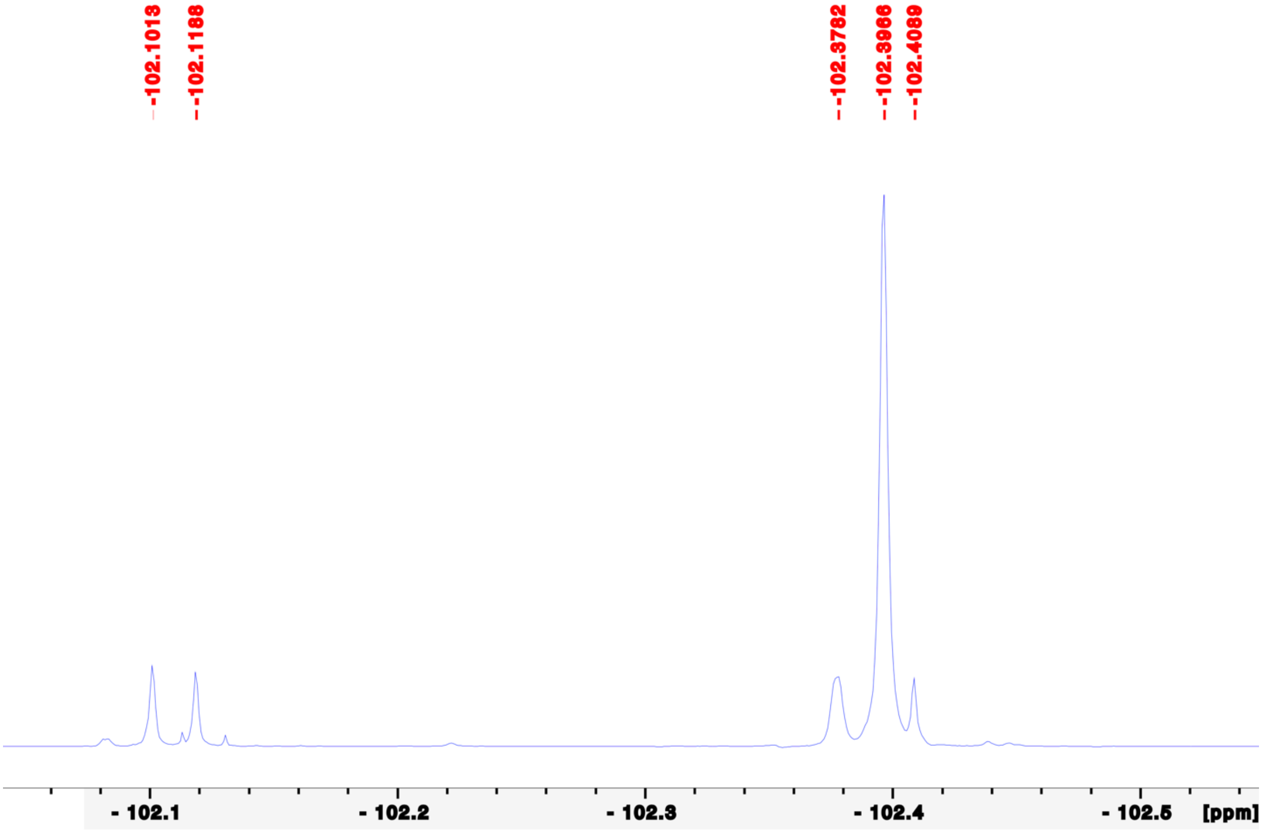

$^{19}F\ \{^{1}H\}$, 471 MHz, $CDCl_3$

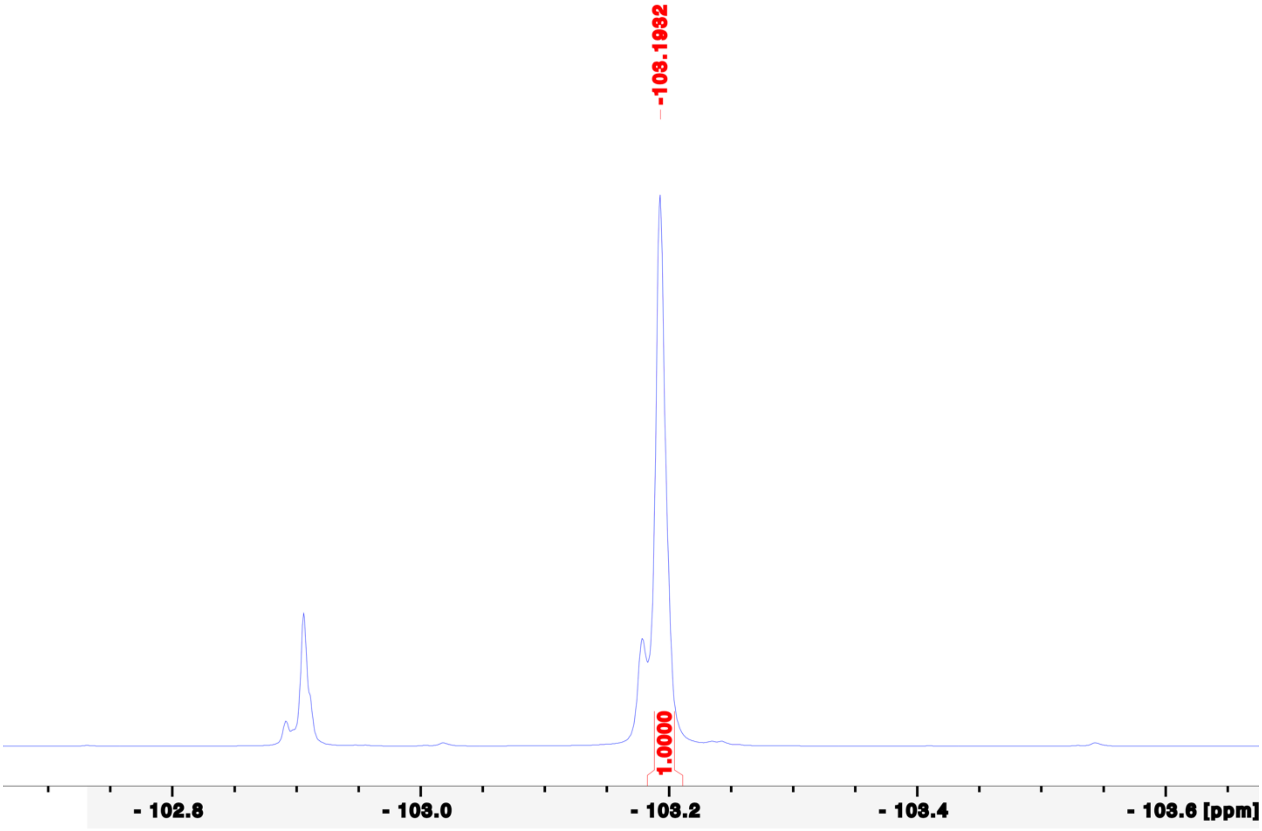

^{2}H, 77 MHz, $CHCl_3$

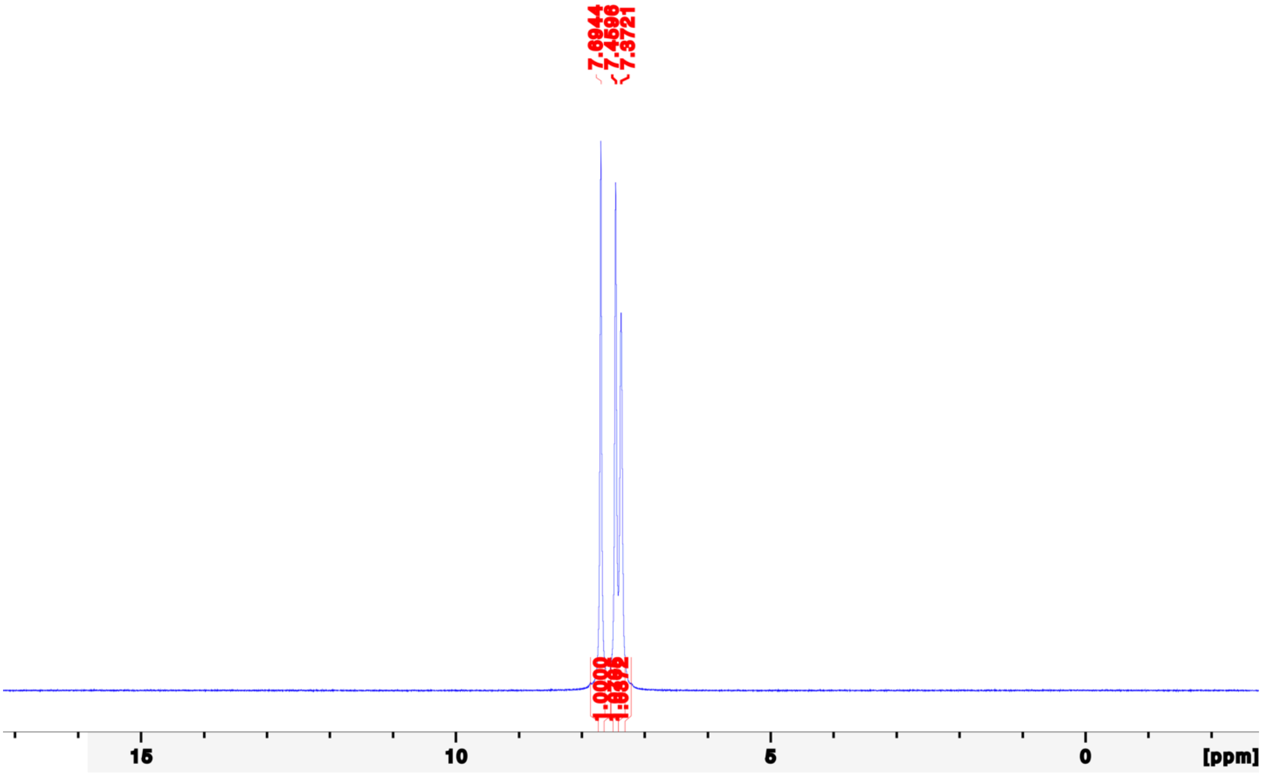

Dimethyl 6,6'-difluoro-[1,1'-biphenyl]-2,2'-dicarboxylate-3,3',4,4',5,5'-*d*$_6$ 13

^{1}H, 500 MHz, $CDCl_3$

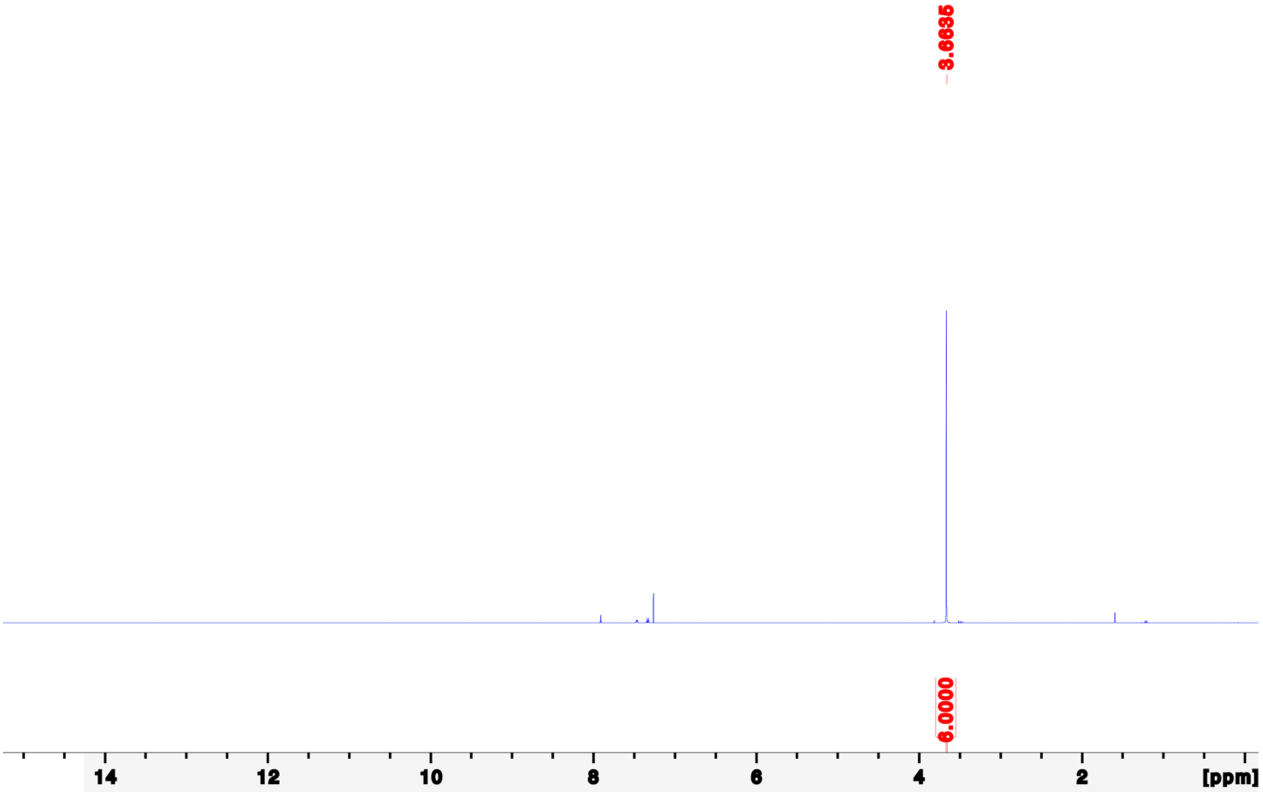

^{13}C, 126 MHz, $CDCl_3$

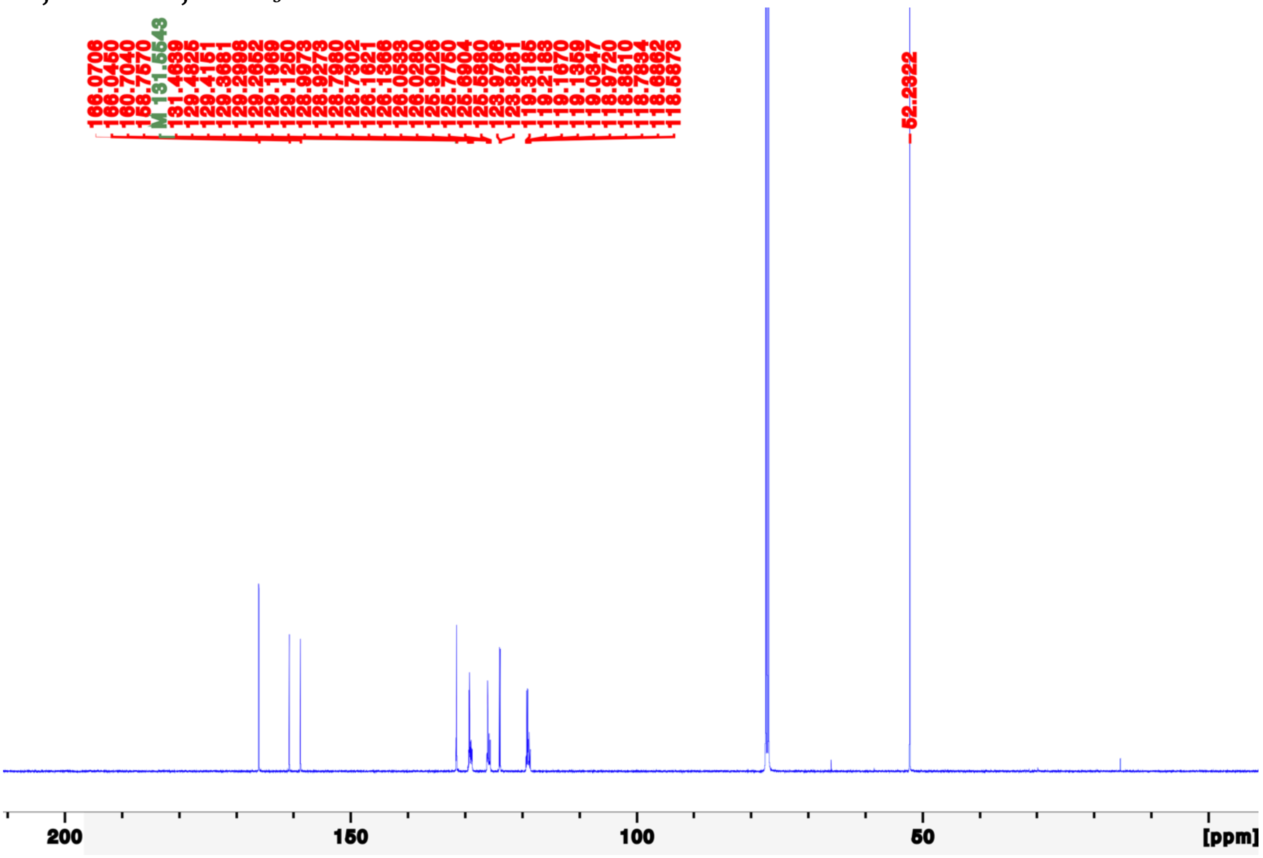

^{19}F, 471 MHz, CDCl$_3$

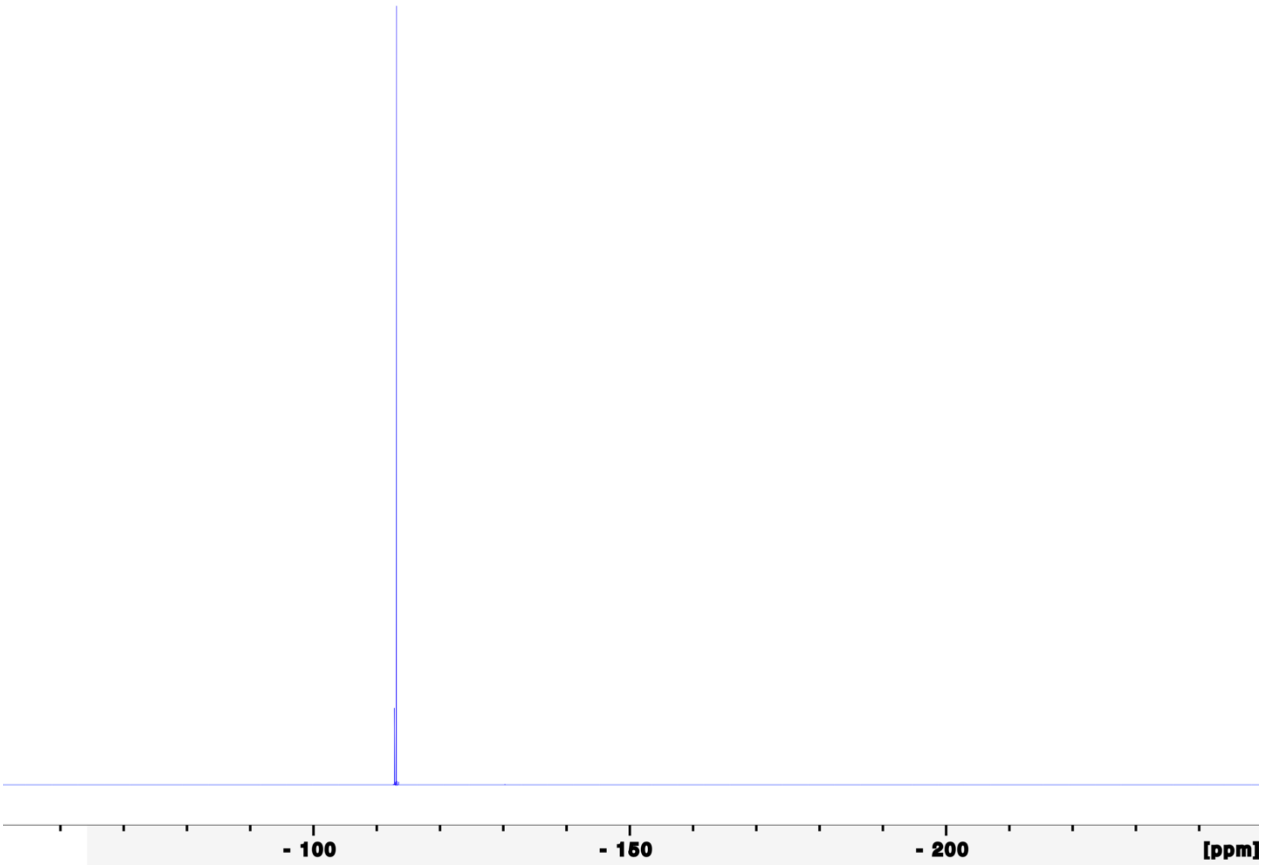

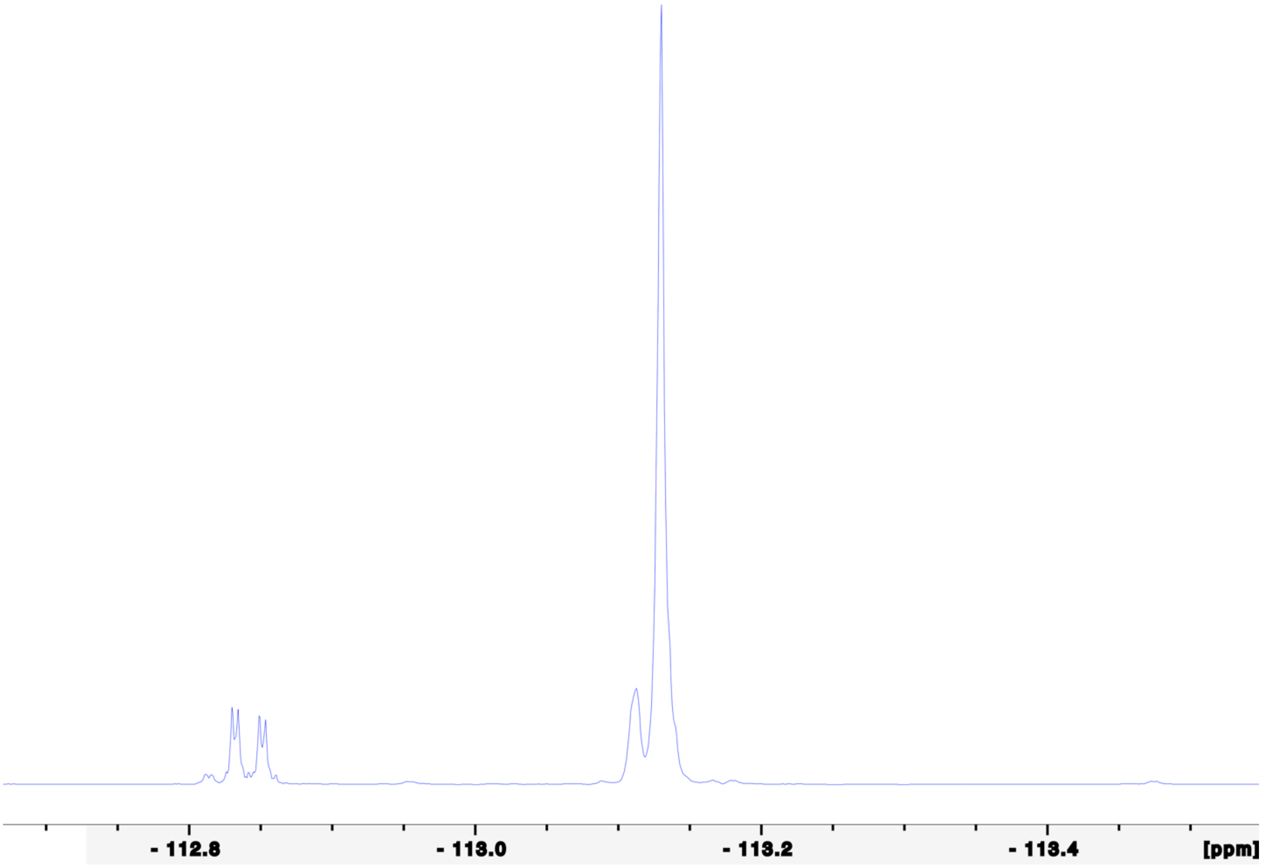

$^{19}F\{^{1}H\}$, 471 MHz, $CDCl_3$

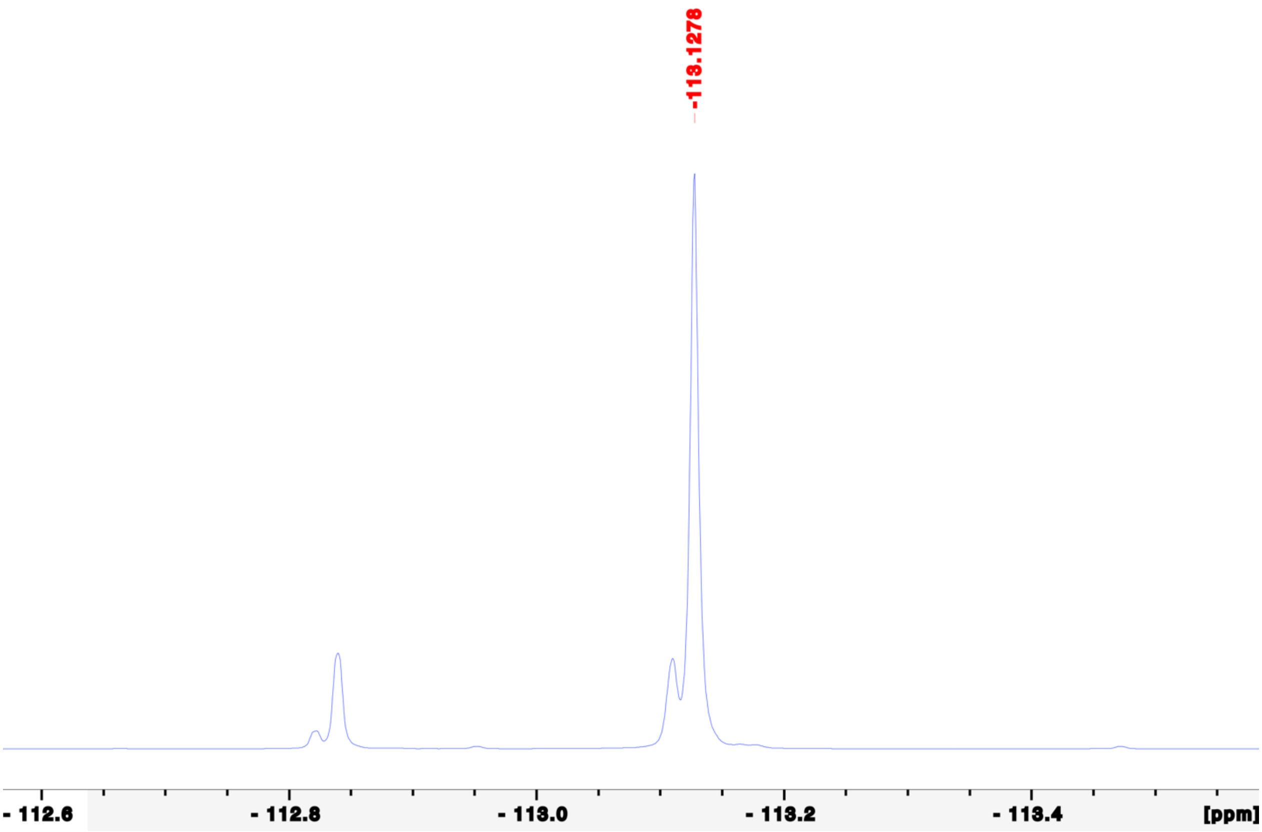

^{2}H, 77 MHz, $CHCl_3$

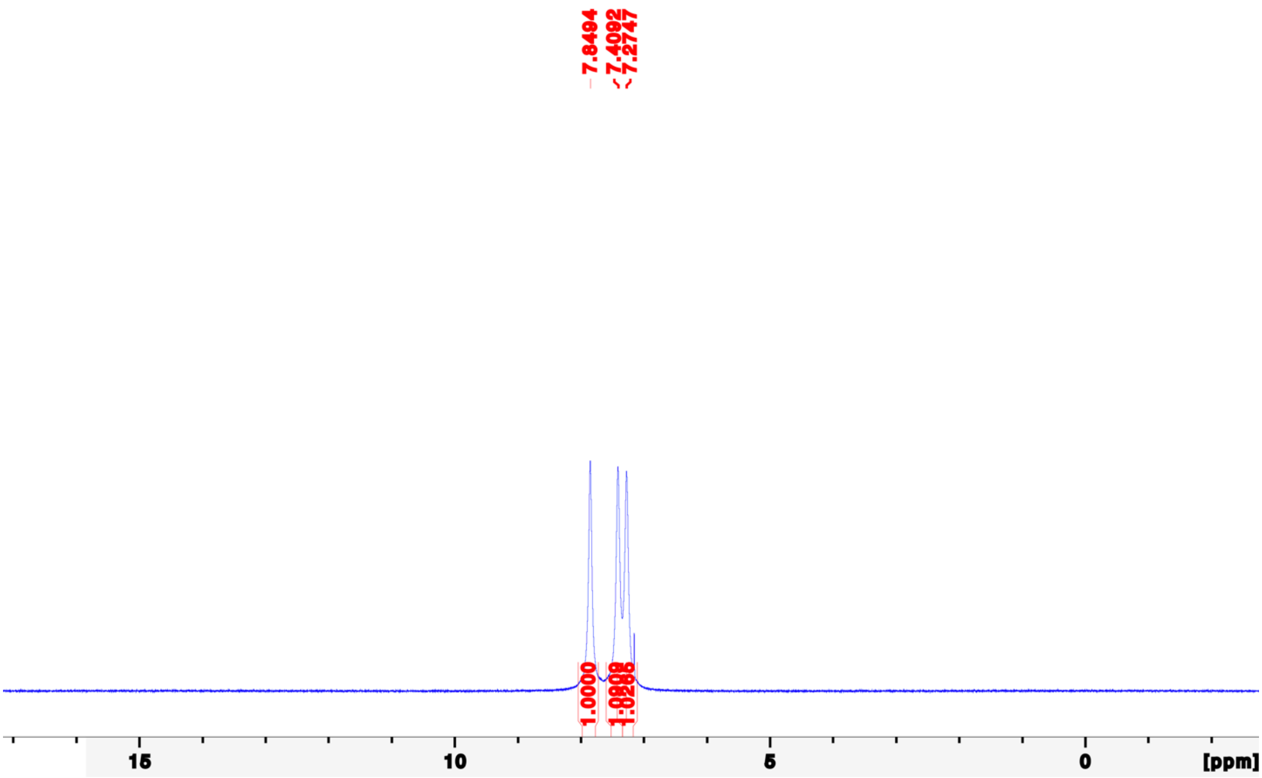

6,6'-Difluoro-[1,1'-biphenyl]-3,3',4,4',5,5'-d_6-2,2'-dicarboxylic acid 7

^{13}C, 126 MHz, CD_3OD

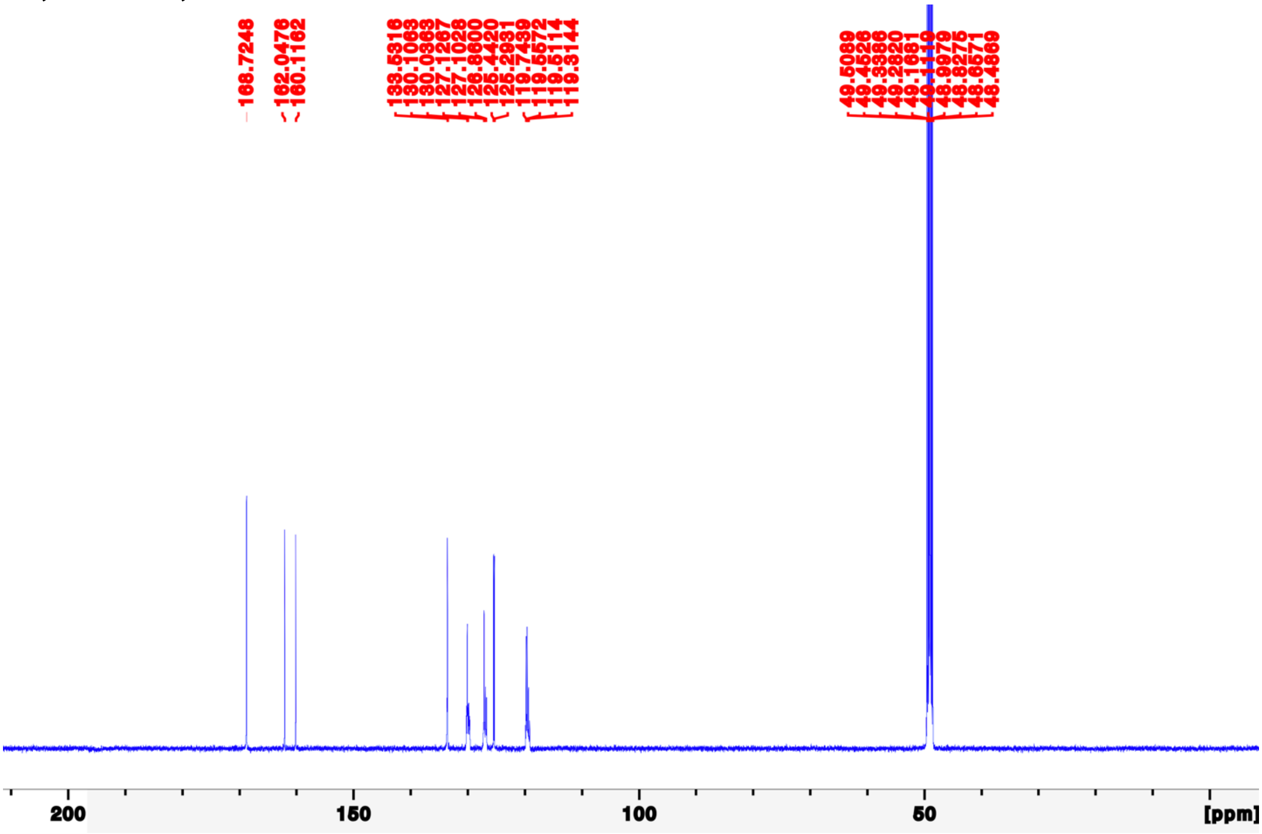

^{19}F, 471 MHz, CD_3OD

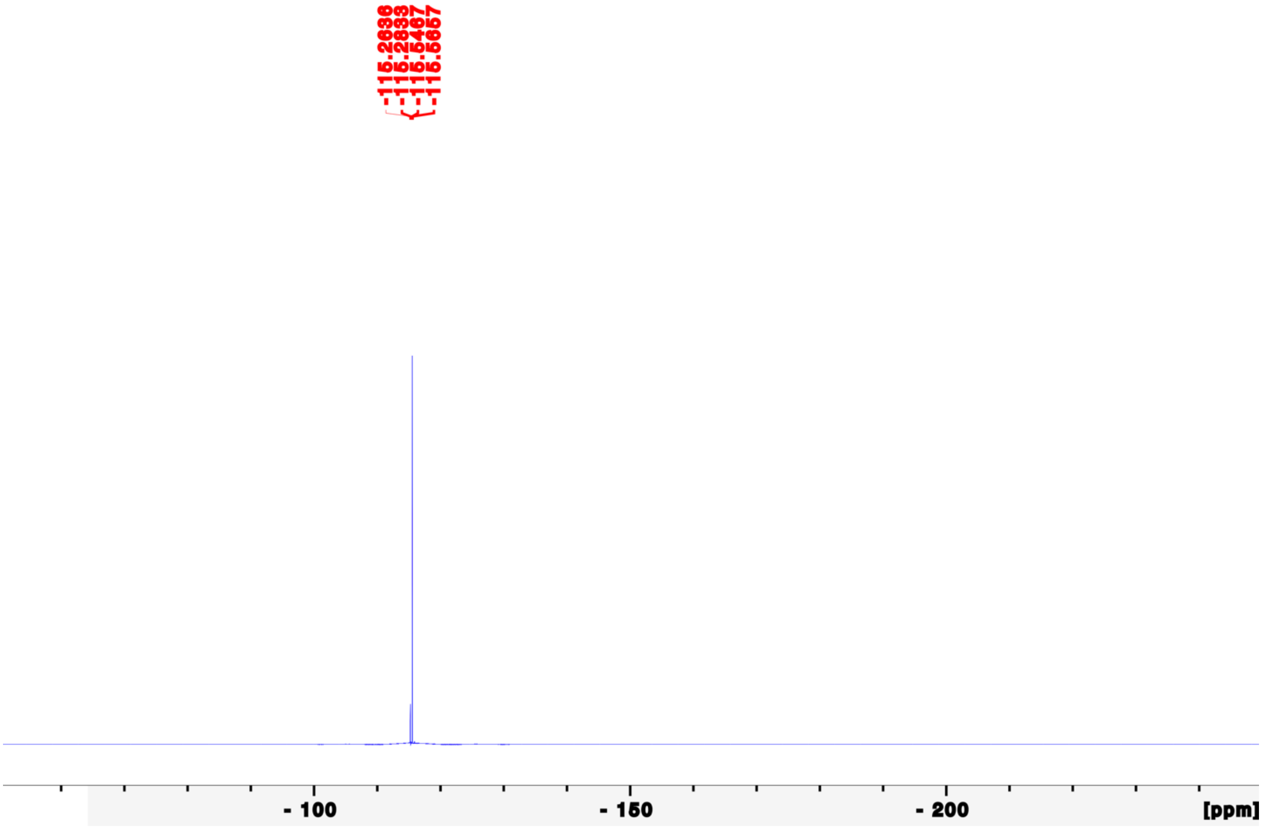

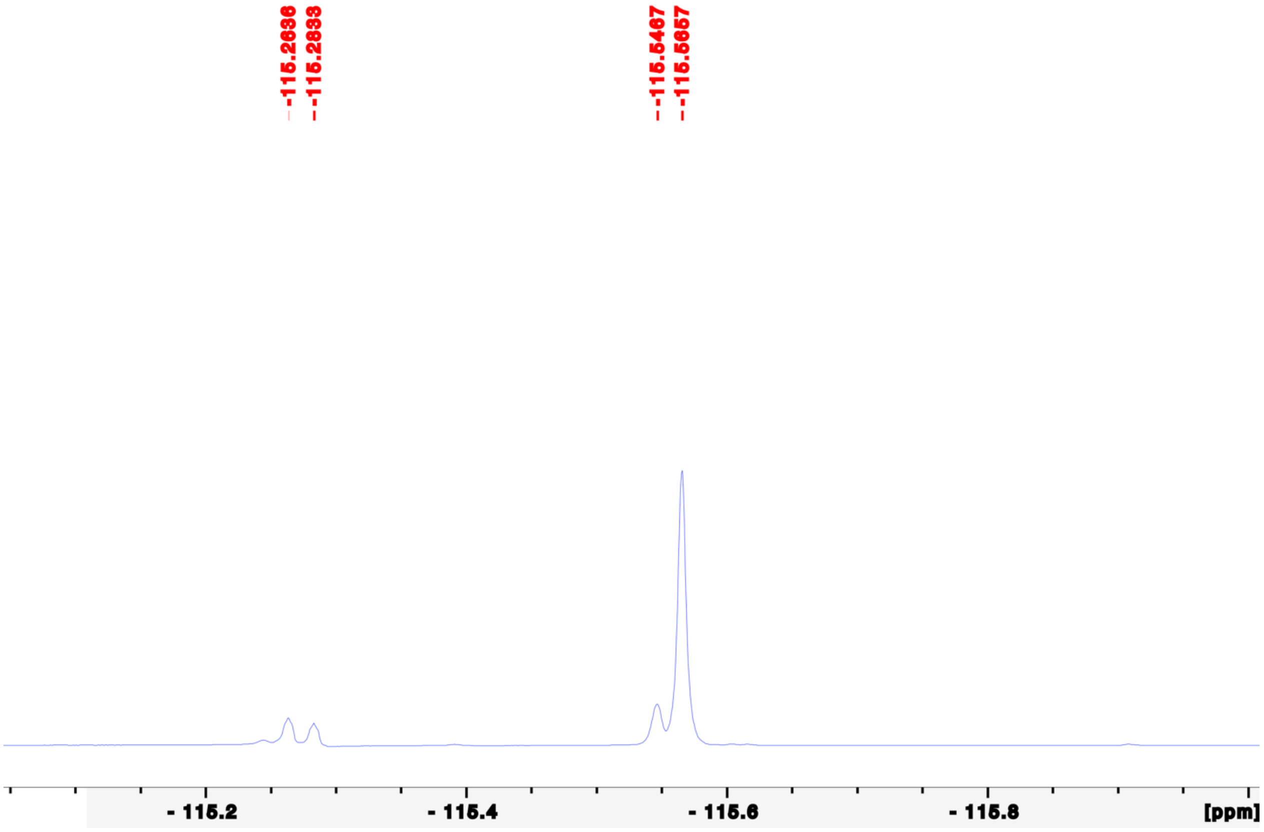

$^{19}F\ \{^{1}H\}$, 471 MHz, CD_3OD

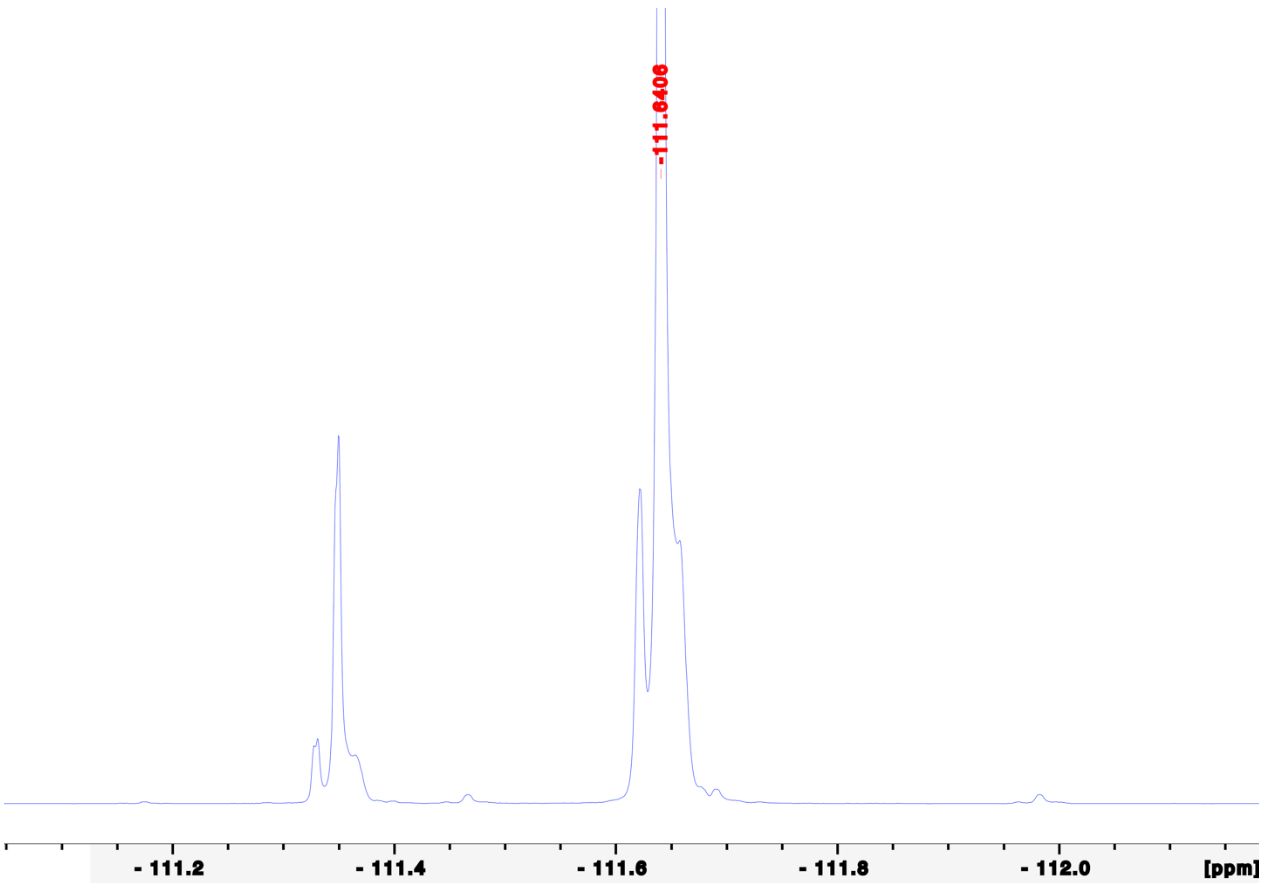

^{2}H, 77 MHz, CH_3OH

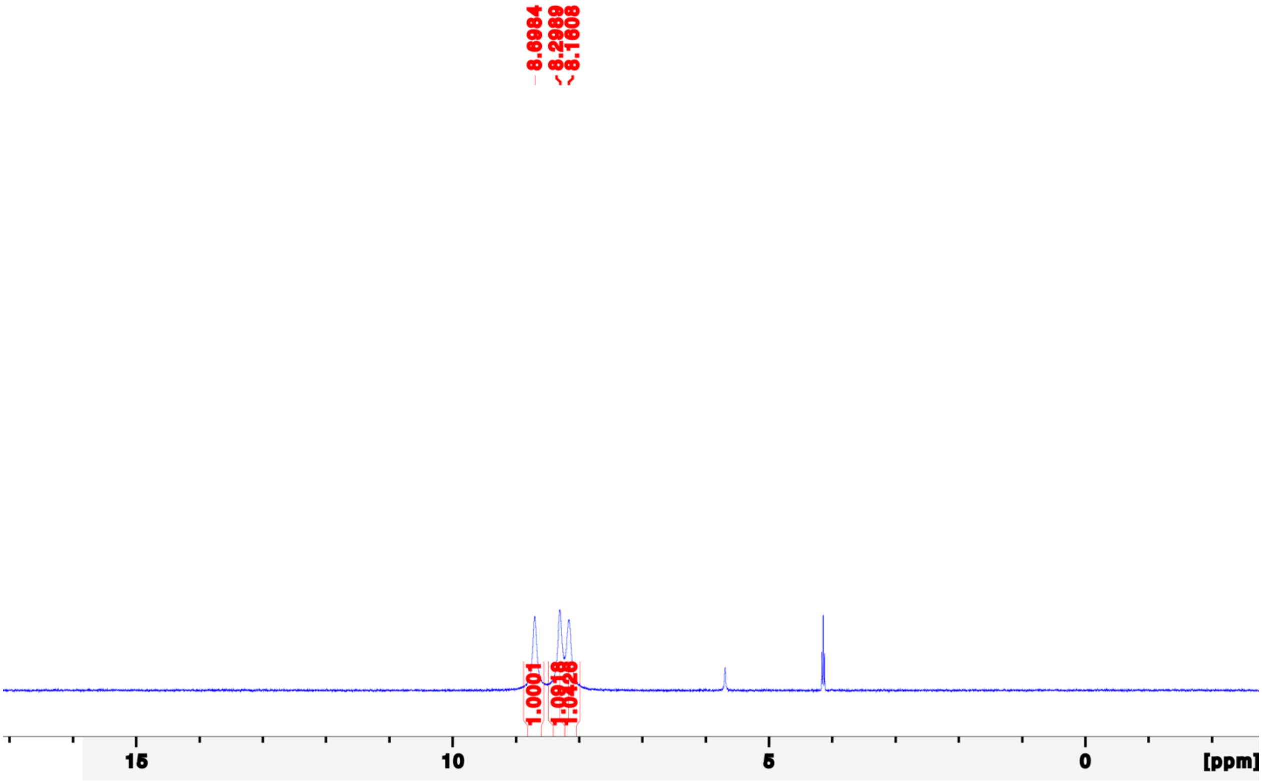

1,10-Difluorophenanthridin-6(5*H*)-one-2,3,4,7,8,9-d_6 ‘Molecule II’

^{1}H, 500 MHz, $(CD_3)_2SO$

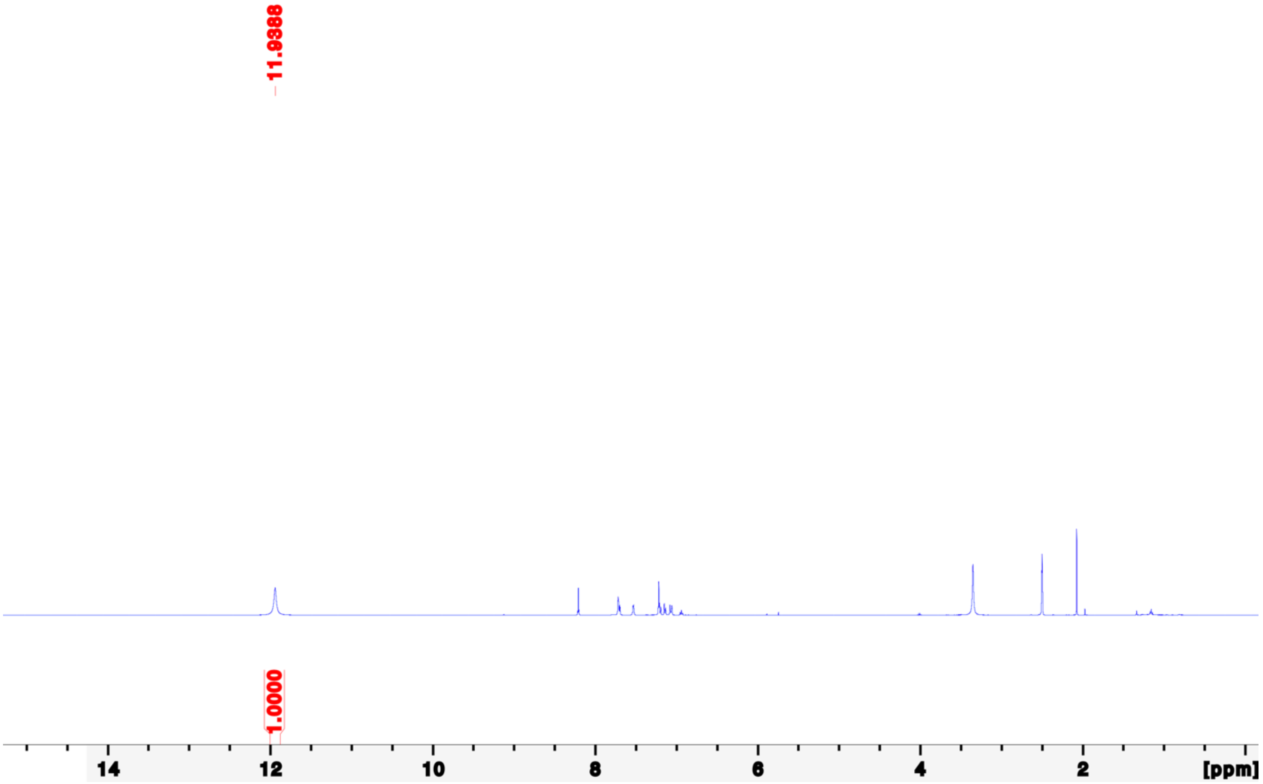

^{13}C, 126 MHz, $(CD_3)_2SO$

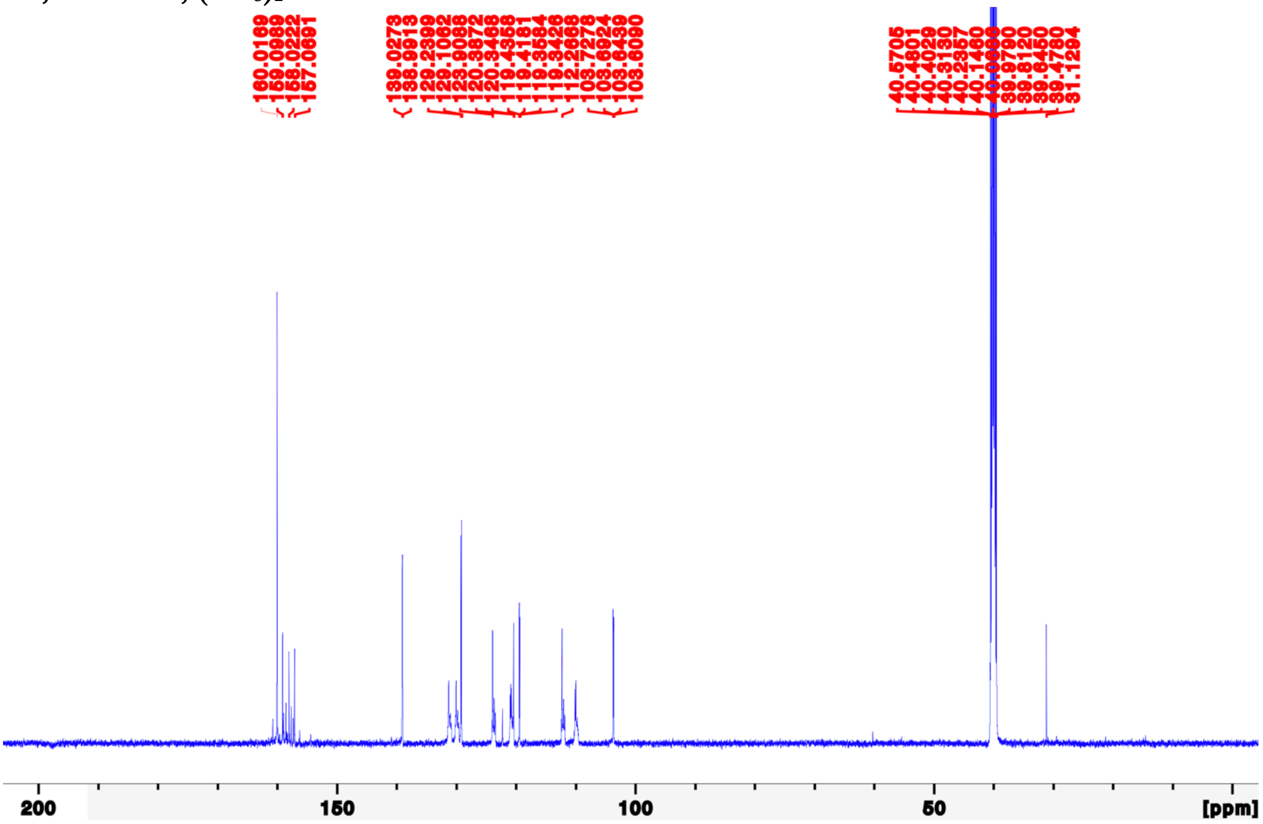

^{19}F, 471 MHz, $(CD_3)_2SO$

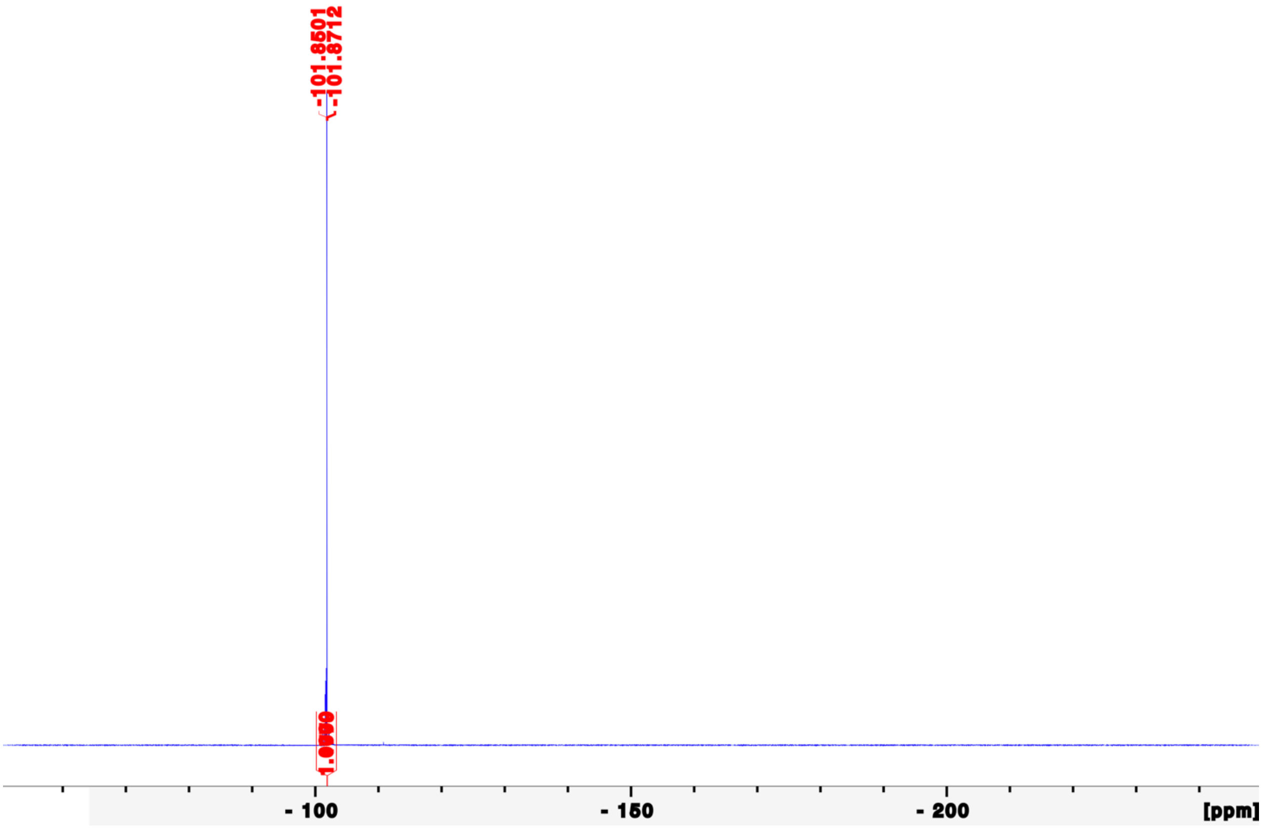

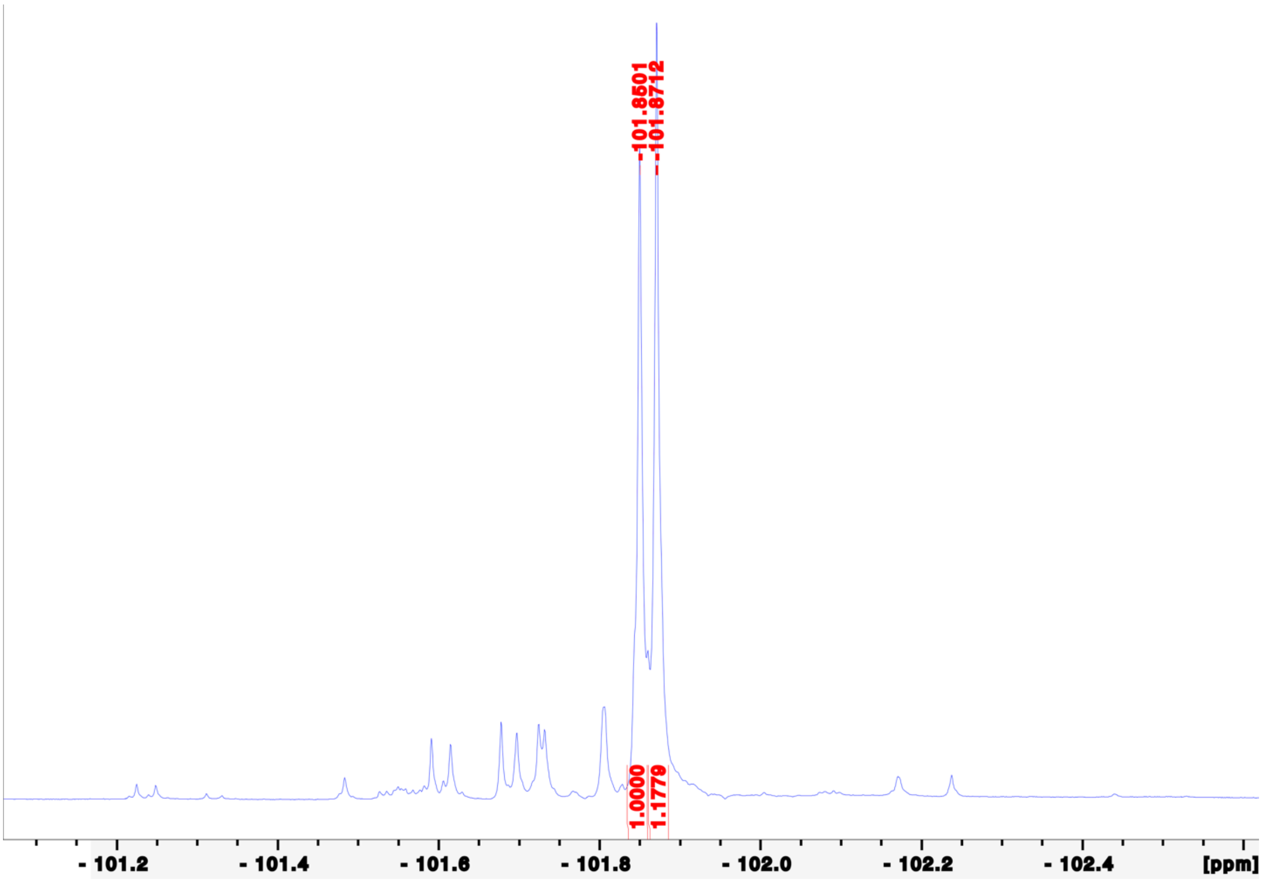

$^{19}F\ \{^{1}H\}$, 471 MHz, $(CD_3)_2SO$

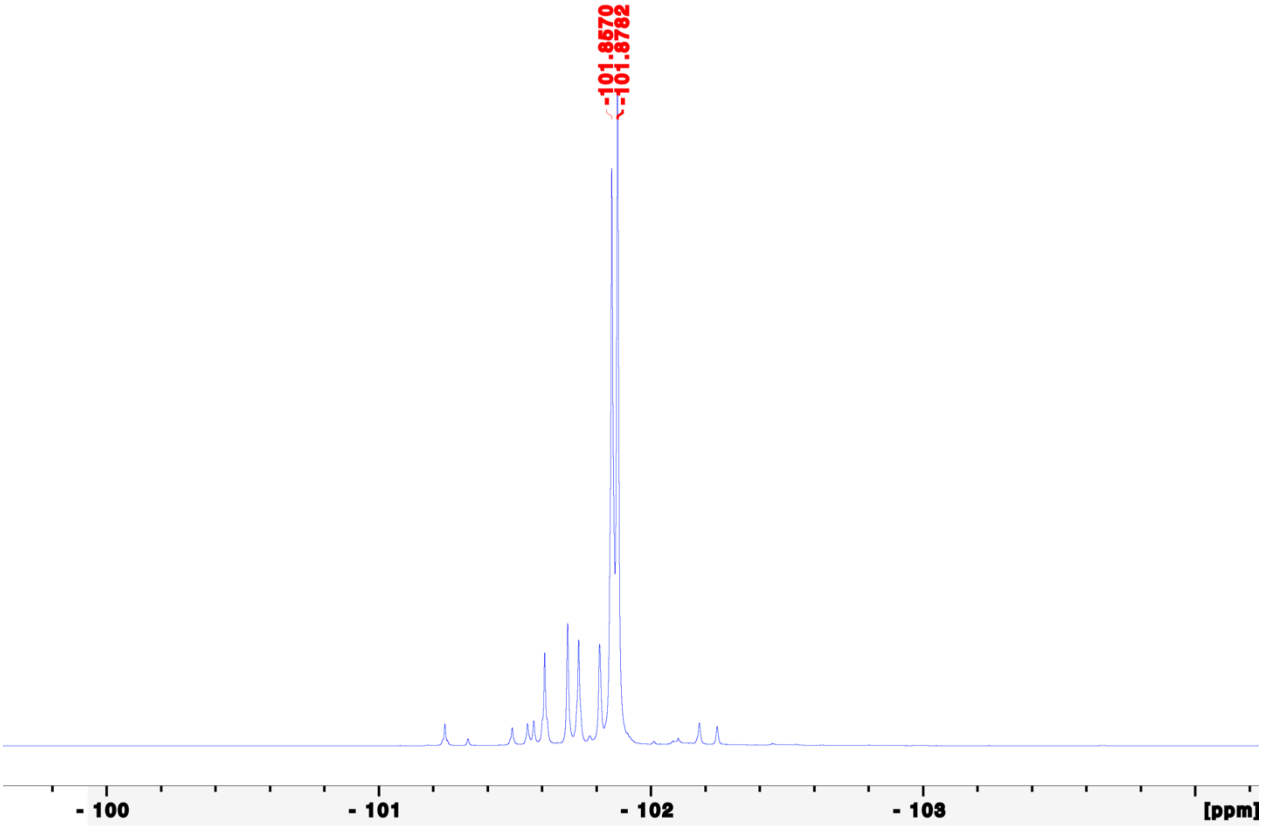

^{2}H, 77 MHz, $(CH_3)_2SO$

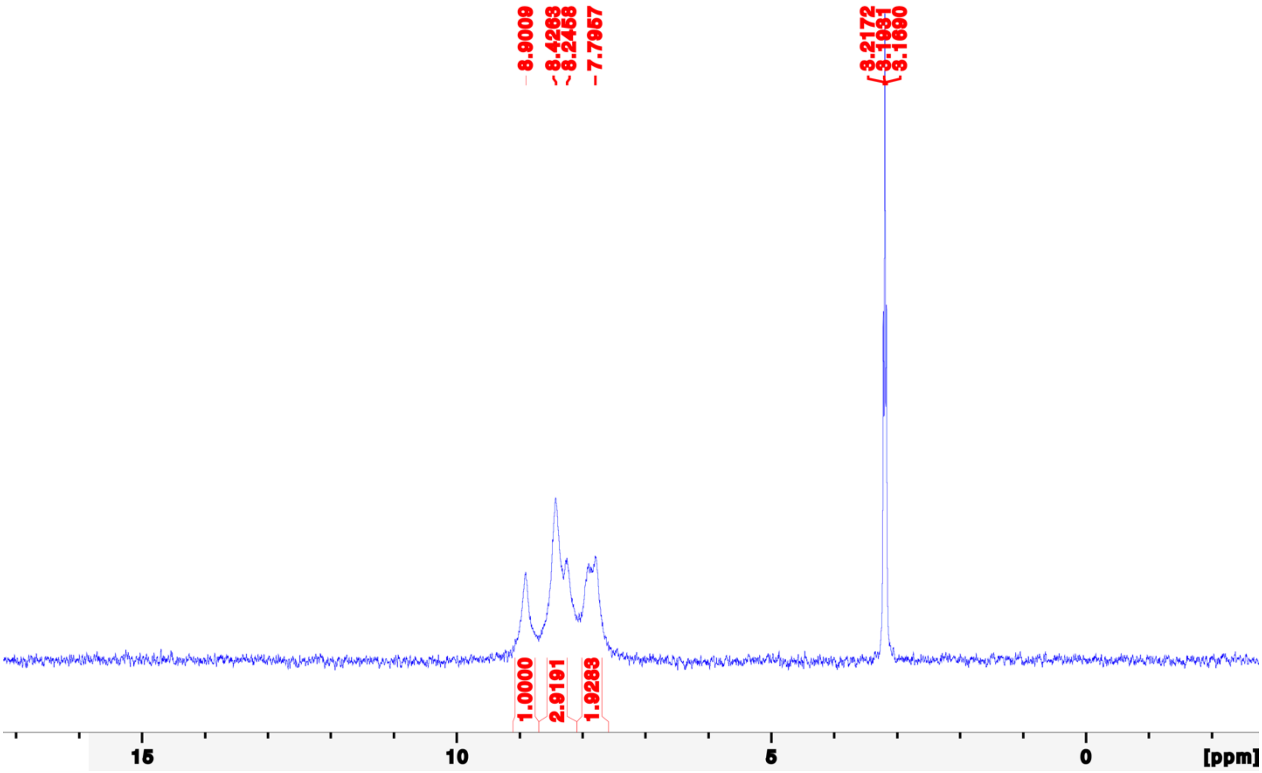

Methyl-d_3 2-bromo-3,4-difluorobenzoate 9

^1H, 500 MHz, $CDCl_3$

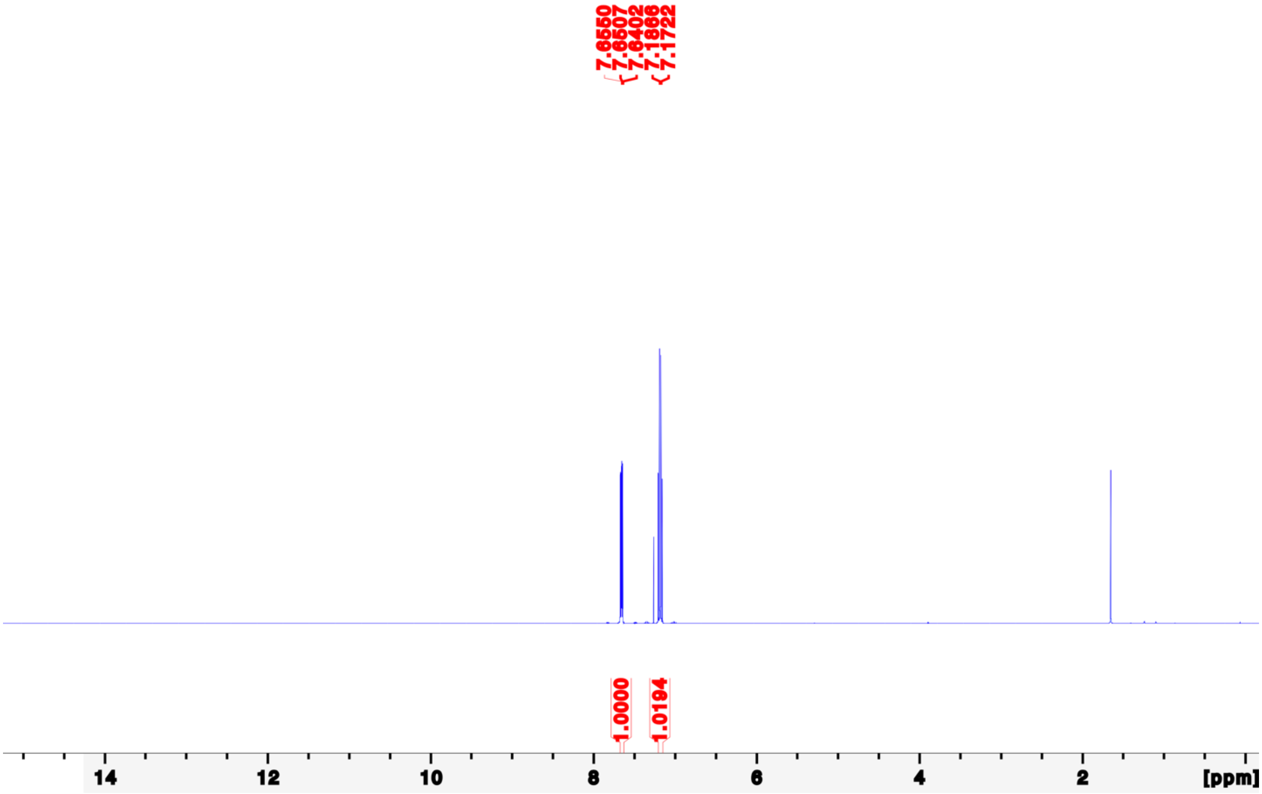

^{13}C, 126 MHz, $CDCl_3$

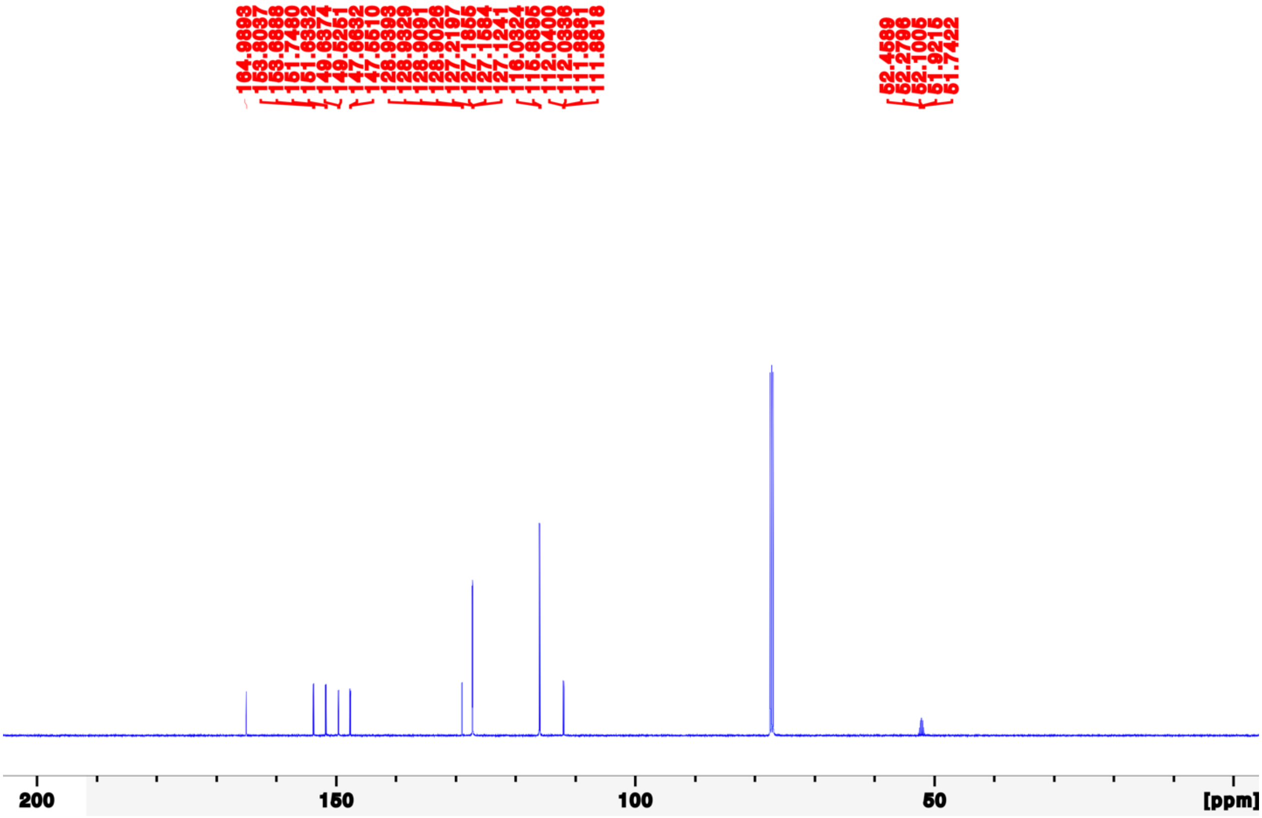

^{19}F, 471 MHz, $CDCl_3$

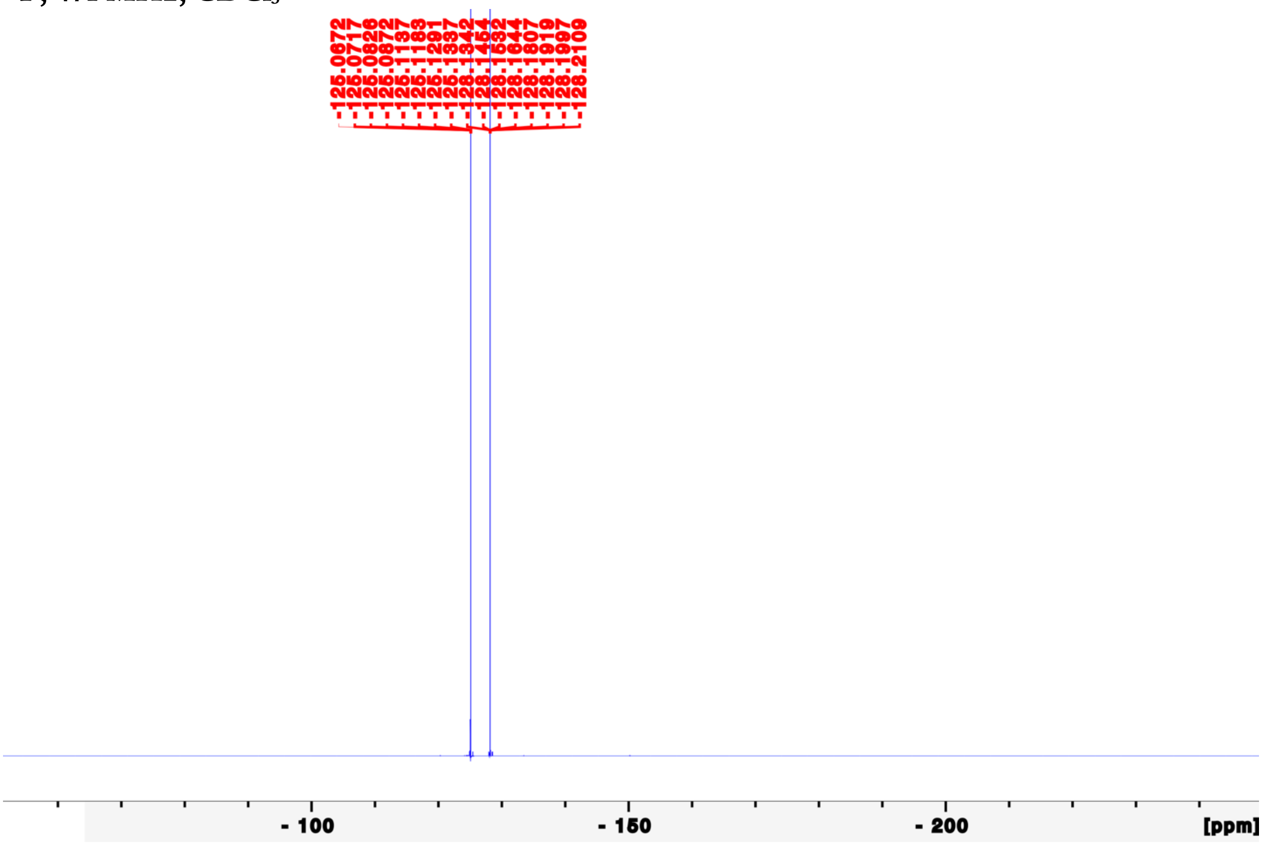

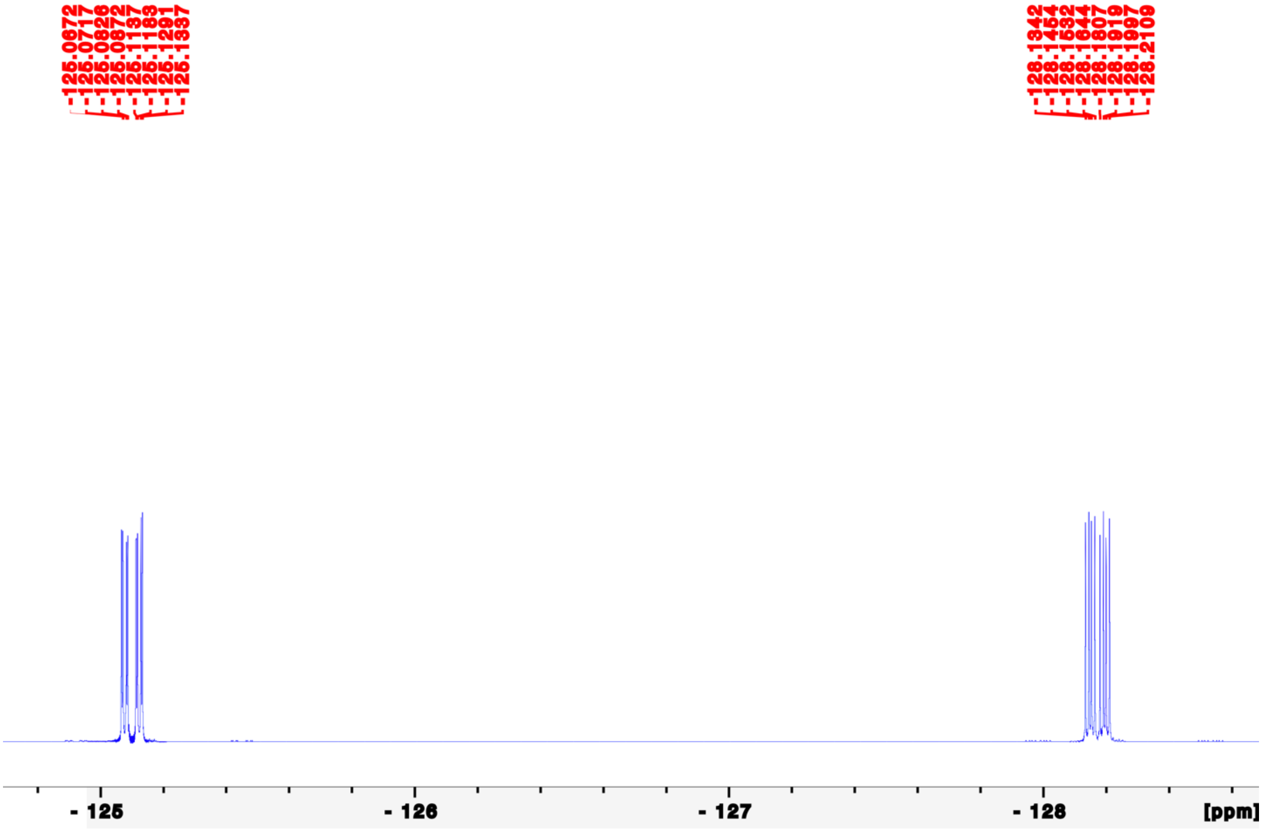

$^{19}F\ \{^{1}H\}$, 471 MHz, $CDCl_3$

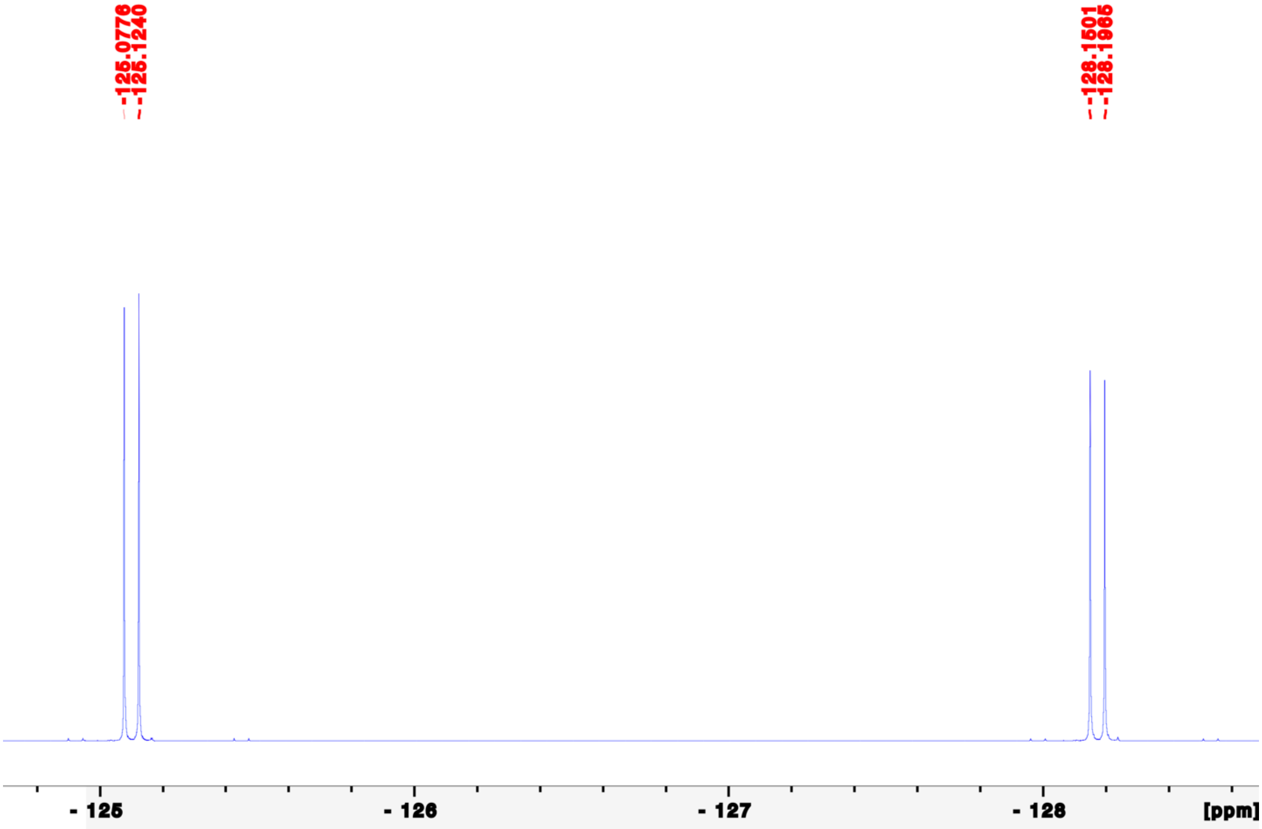

^{2}H, 77 MHz, $CHCl_3$

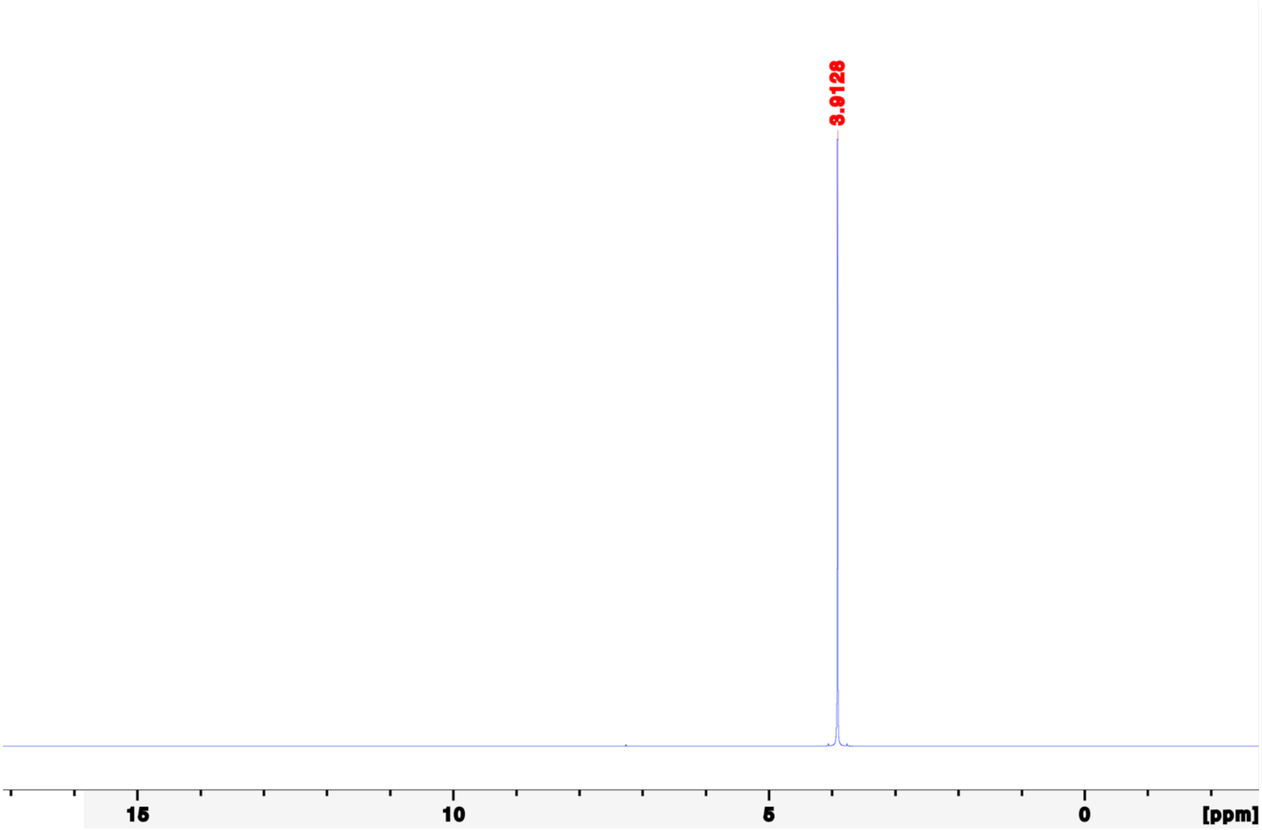

Methyl-d_3 2-bromo-3-fluoro-4-(methoxy-d_3) benzoate 10

^{1}H, 500 MHz, $CDCl_3$

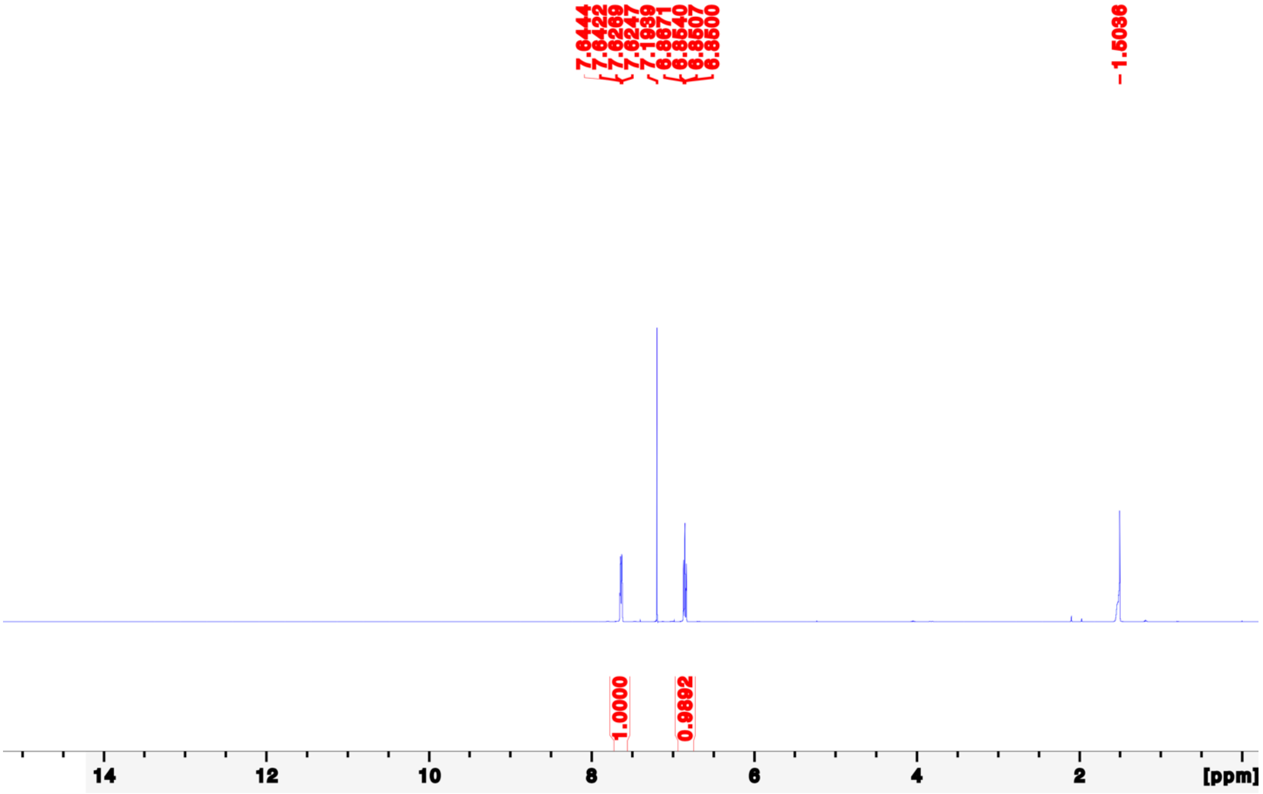

^{13}C, 126 MHz, $CDCl_3$

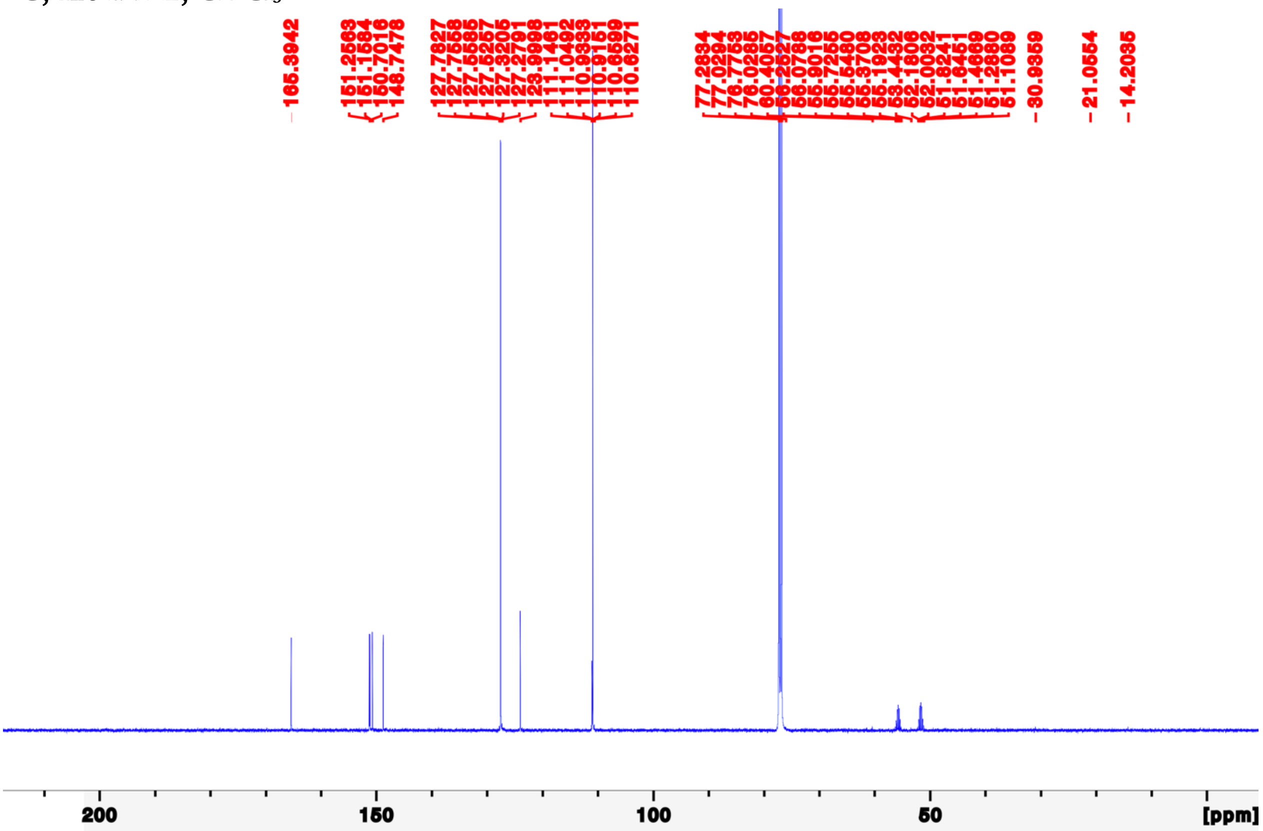

^{19}F, 471 MHz, $CDCl_3$

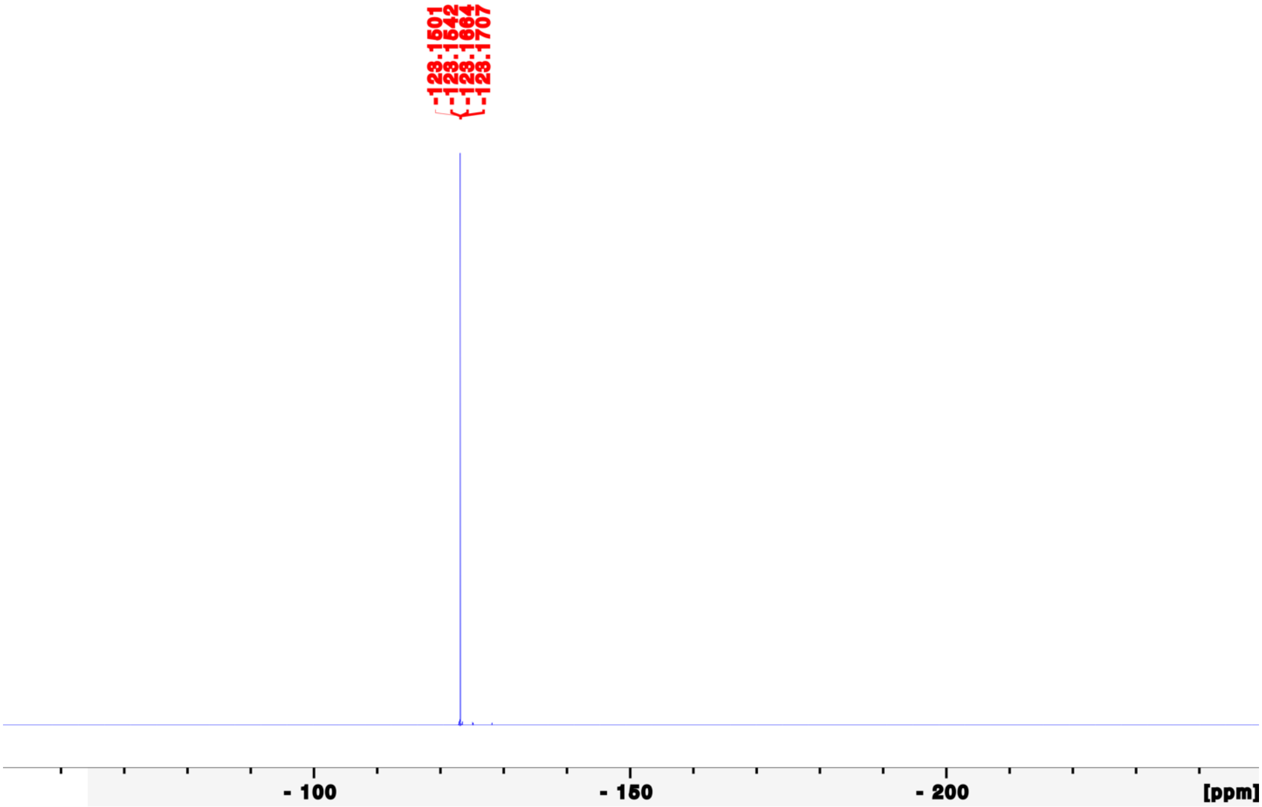

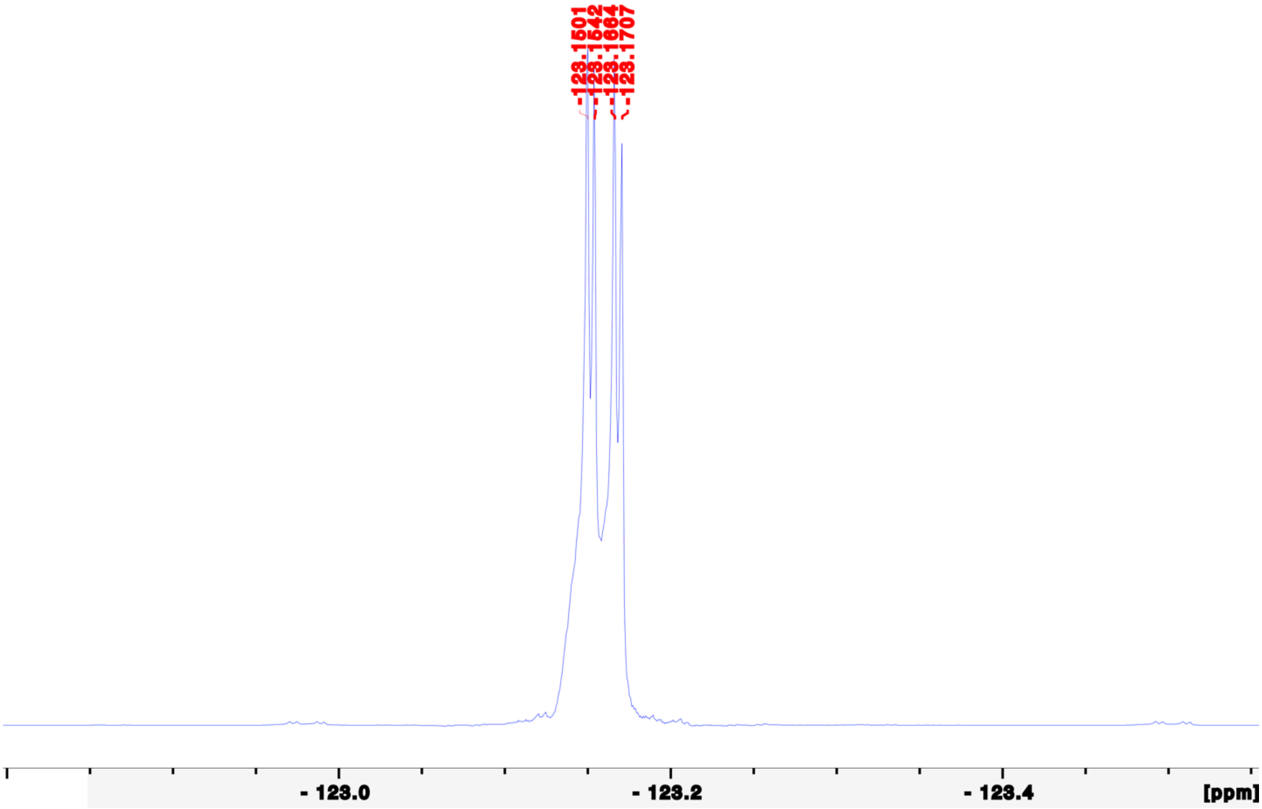

^{19}F {^{1}H}, 471 MHz, $CDCl_3$

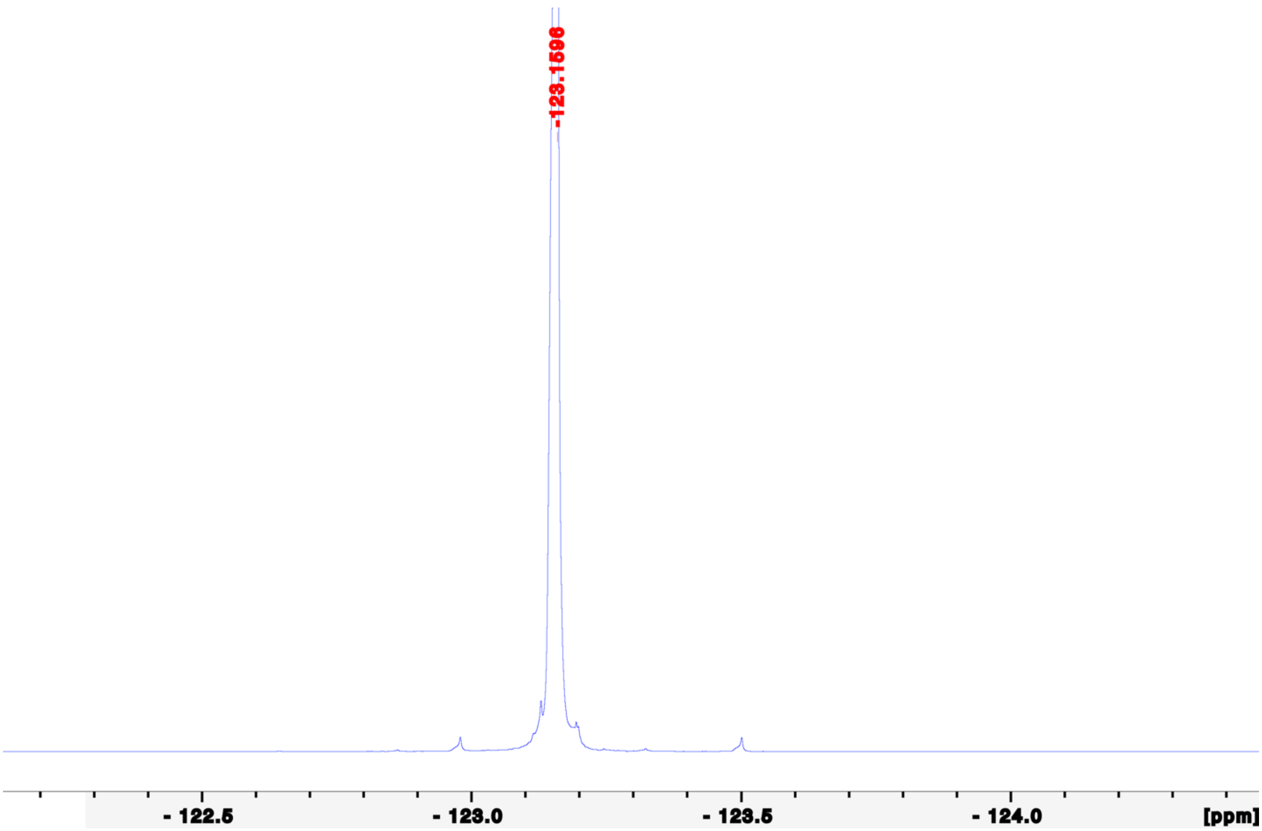

^{2}H, 77 MHz, $CHCl_3$

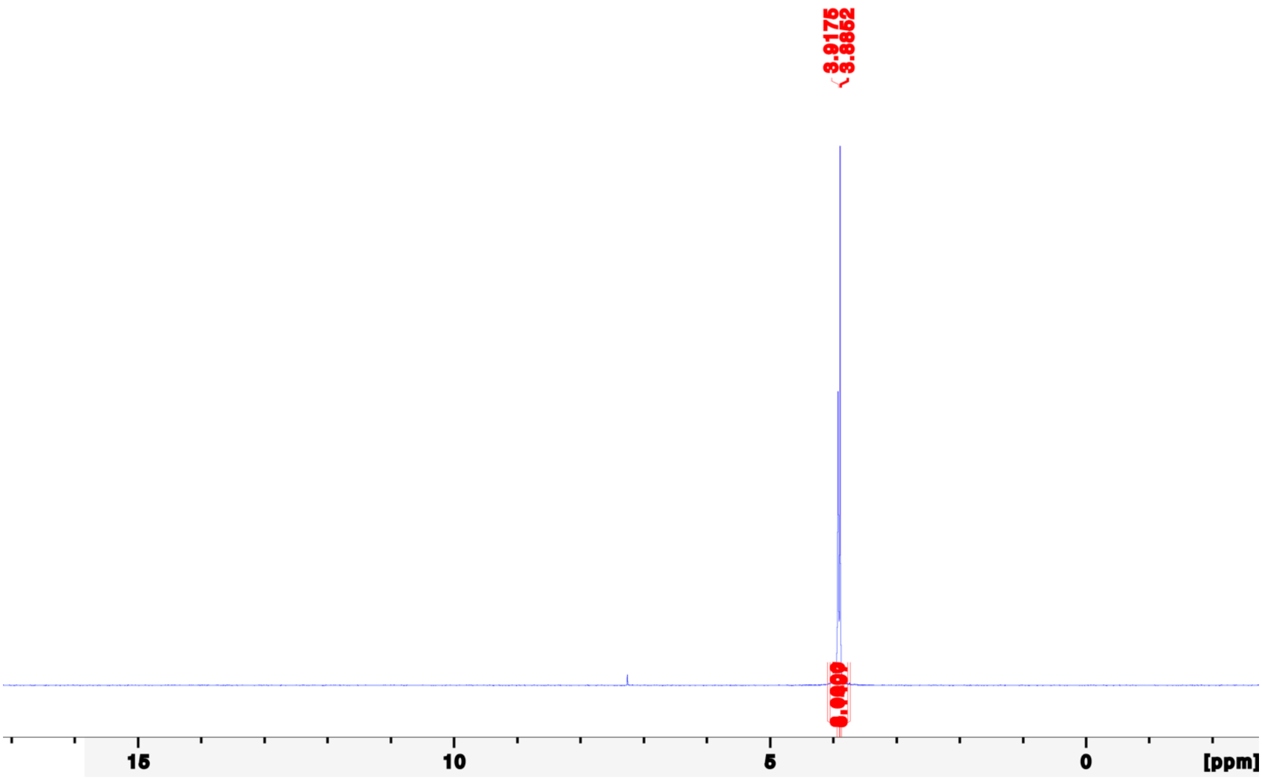

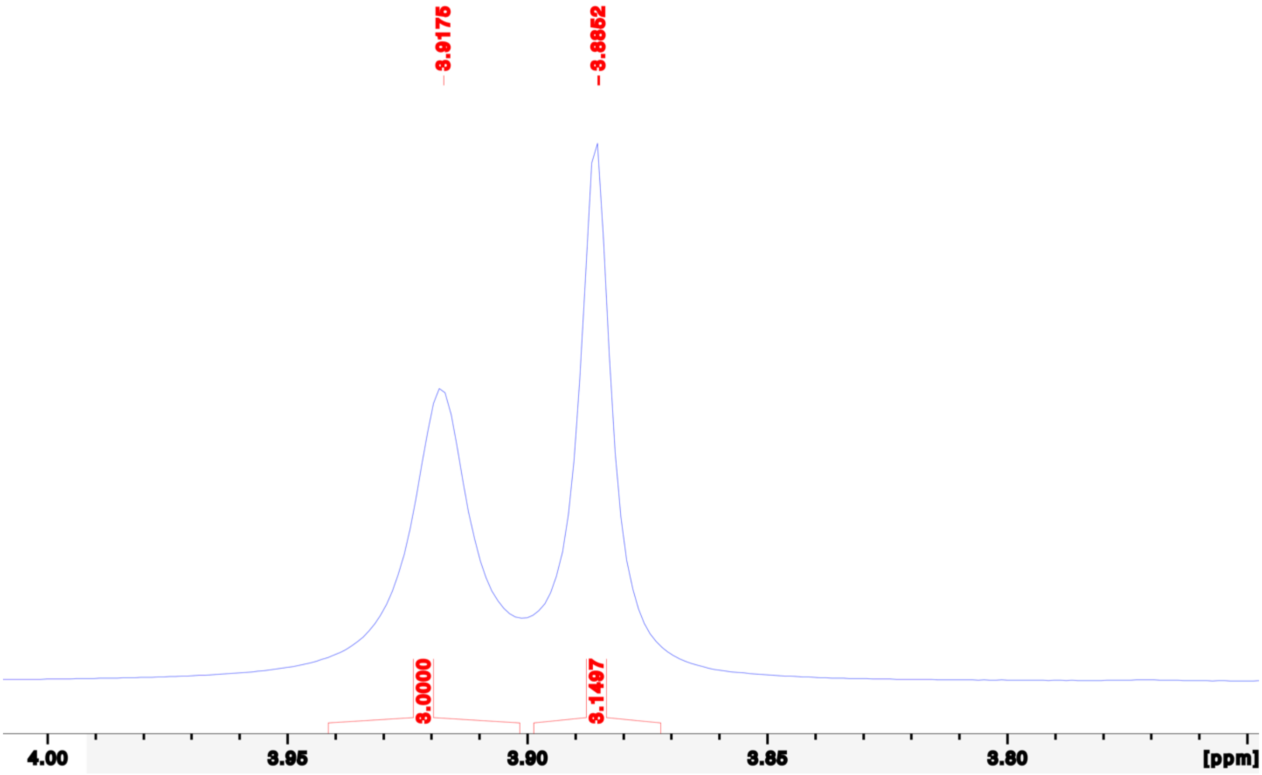

3.9175
3.8852
3.0000
3.1497
4.00
3.95
3.90
3.85
3.80
[ppm]

Bis(methyl-d_3) 6,6'-difluoro-5,5'-bis(methoxy-d_3)-[1,1'-biphenyl]-2,2'-dicarboxylate 14

^{1}H, 500 MHz, $CDCl_3$

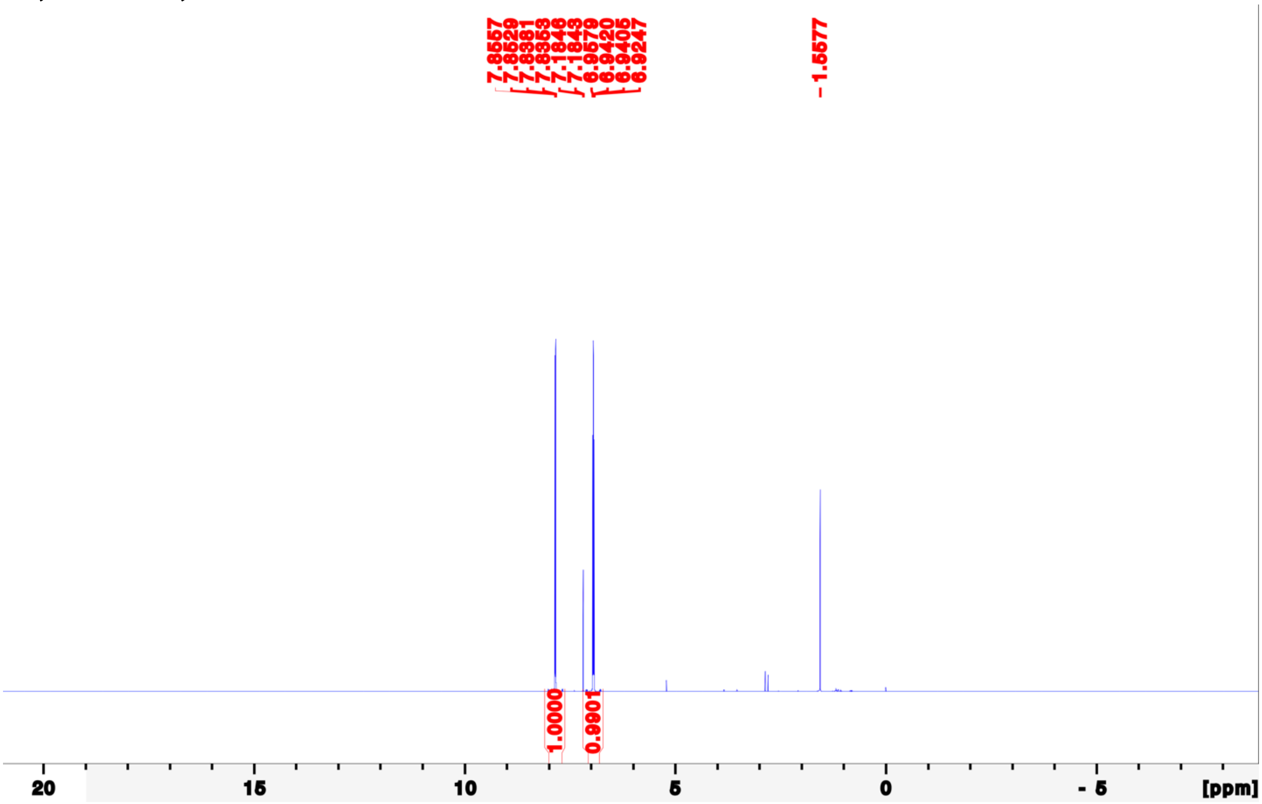

^{13}C, 126 MHz, $CDCl_3$

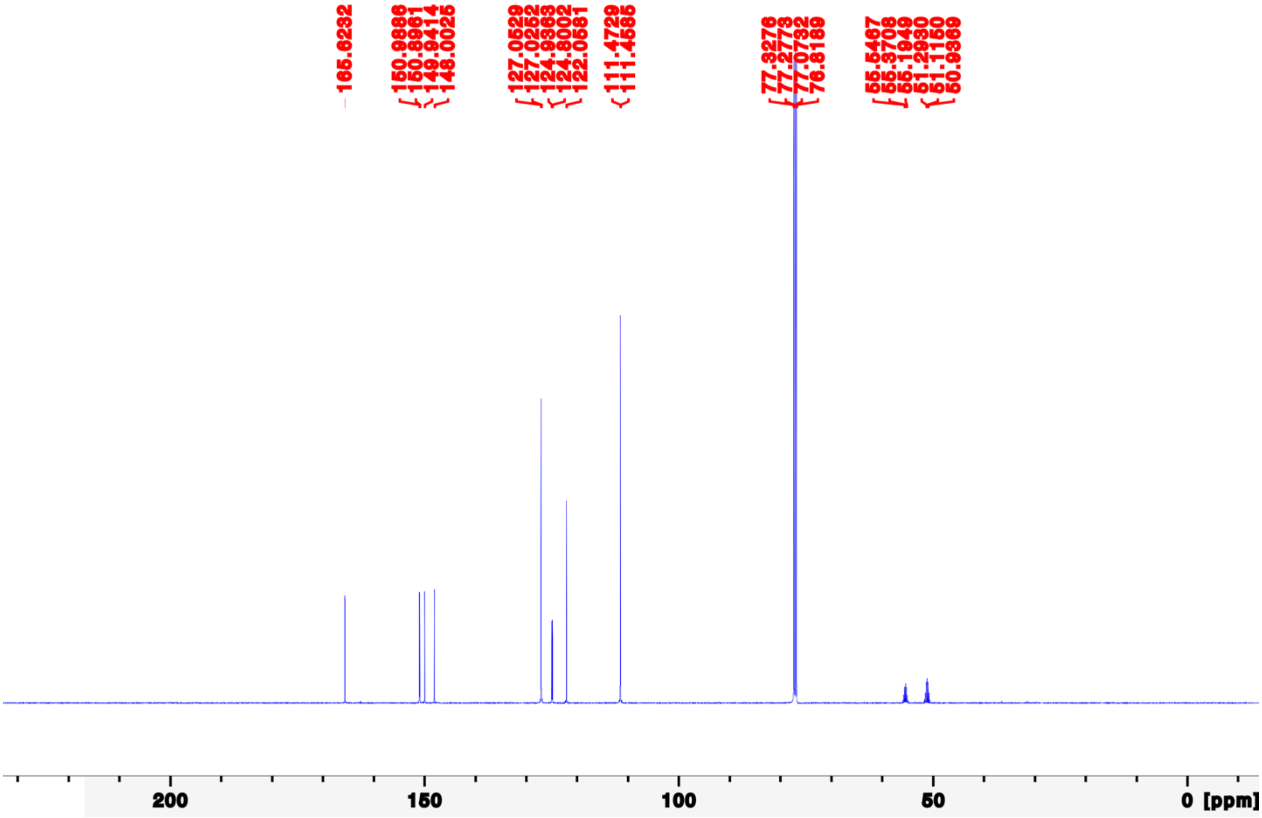

^{19}F, 471 MHz, $CDCl_3$

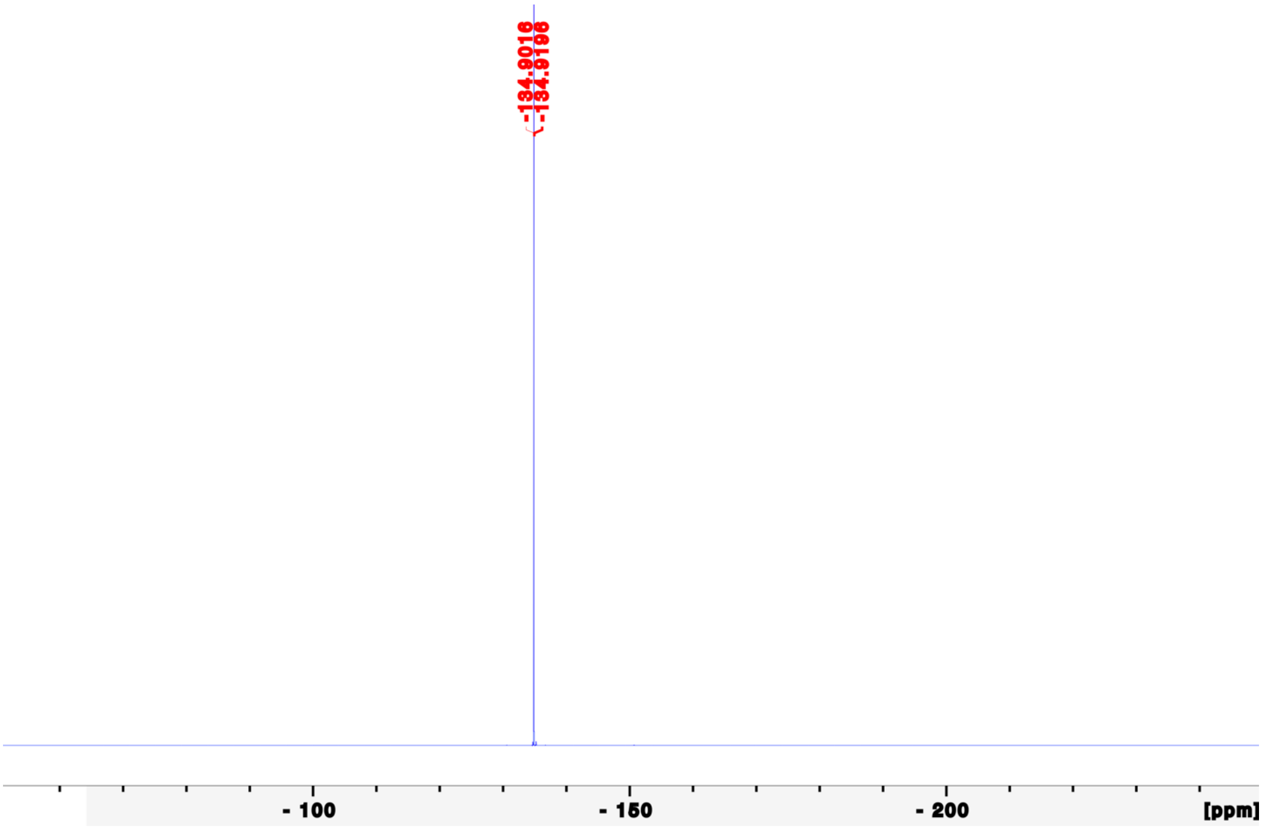

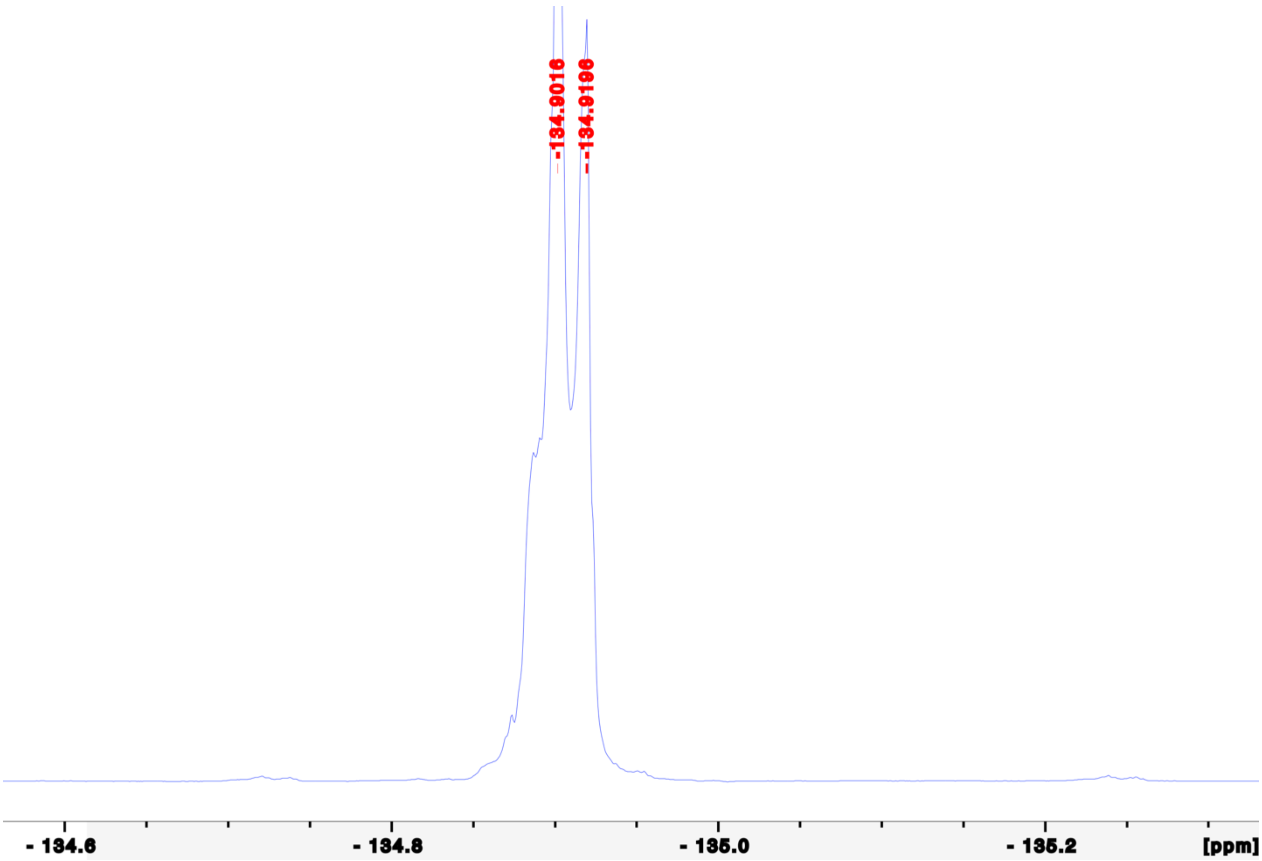

$^{19}F\ \{^{1}H\}$, 471 MHz, $CDCl_3$

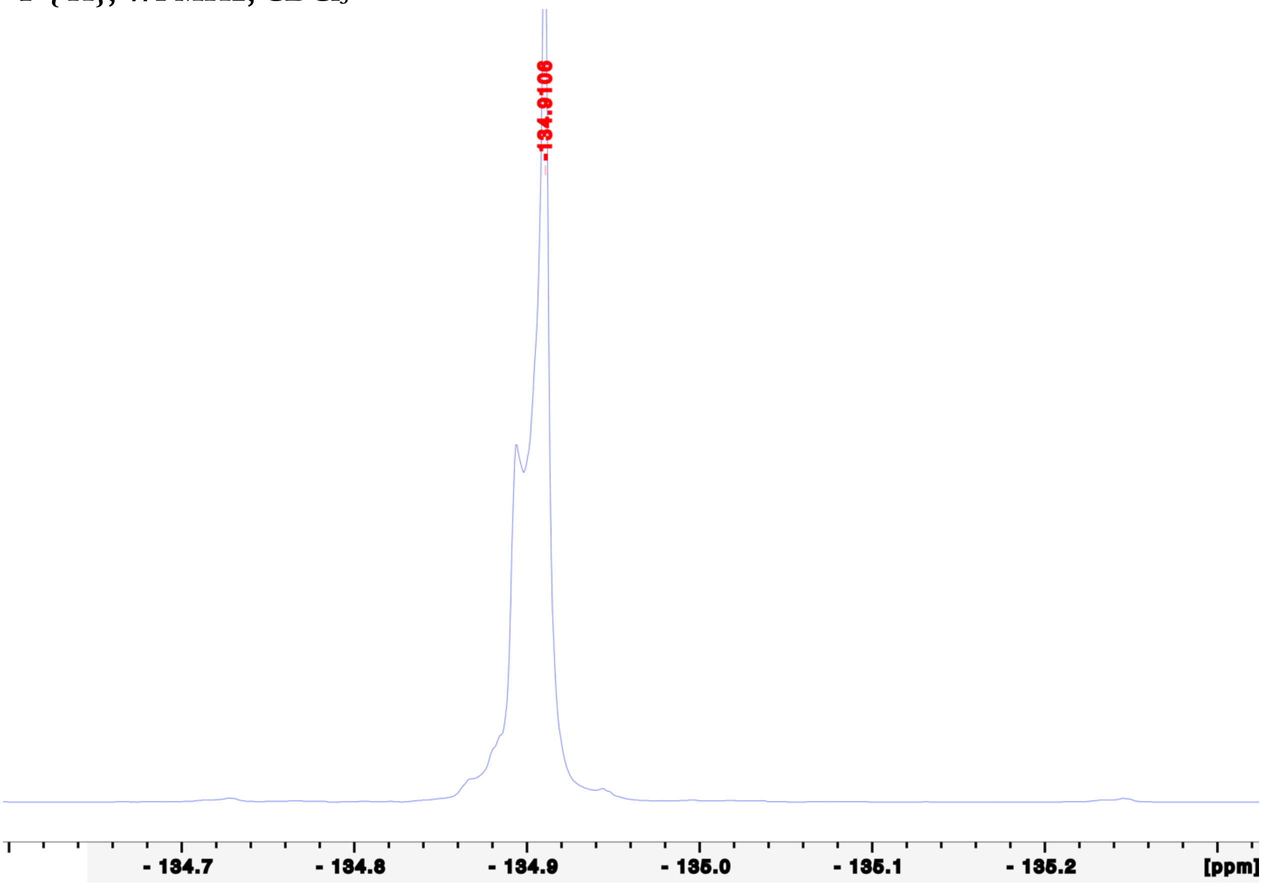

^{2}H, 77 MHz, $CHCl_3$

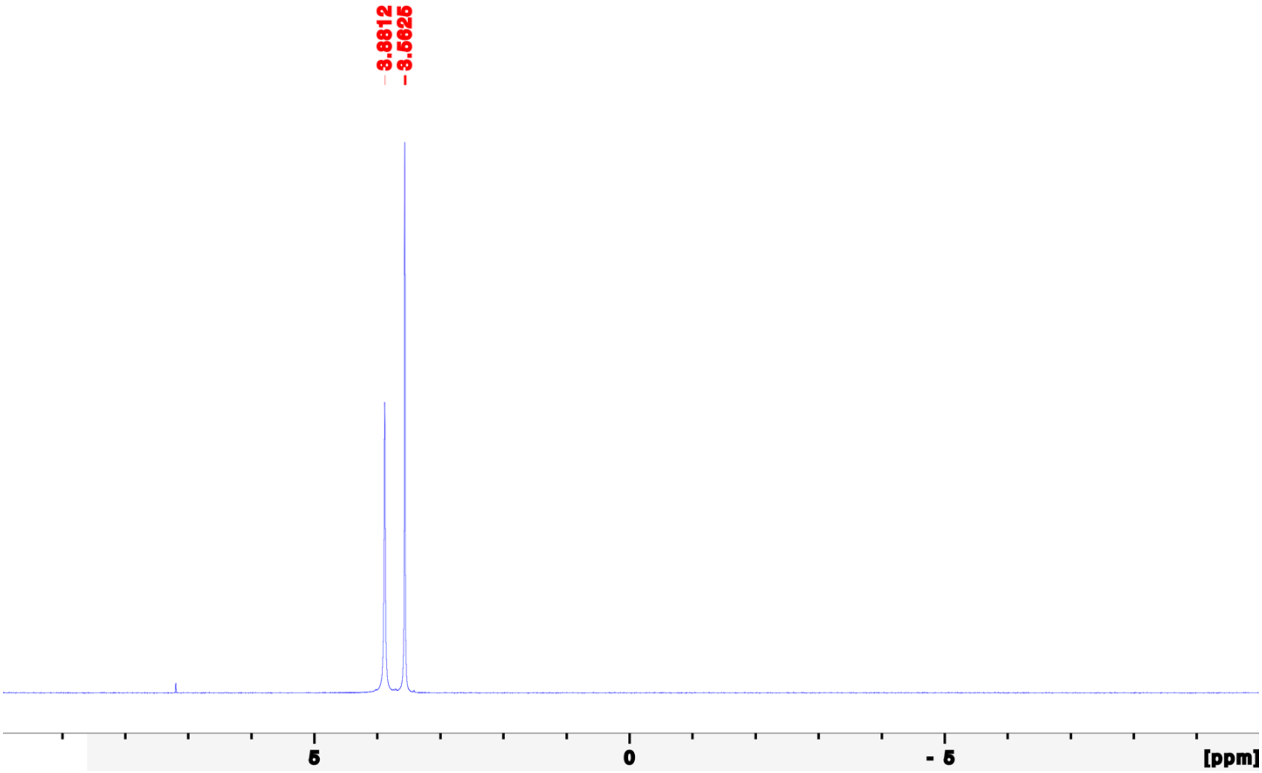

6,6'-Difluoro-5,5'-bis(methoxy-d_3)-[1,1'-biphenyl]-2,2'-dicarboxylic acid 15

^{1}H, 500 MHz, CD_3OD

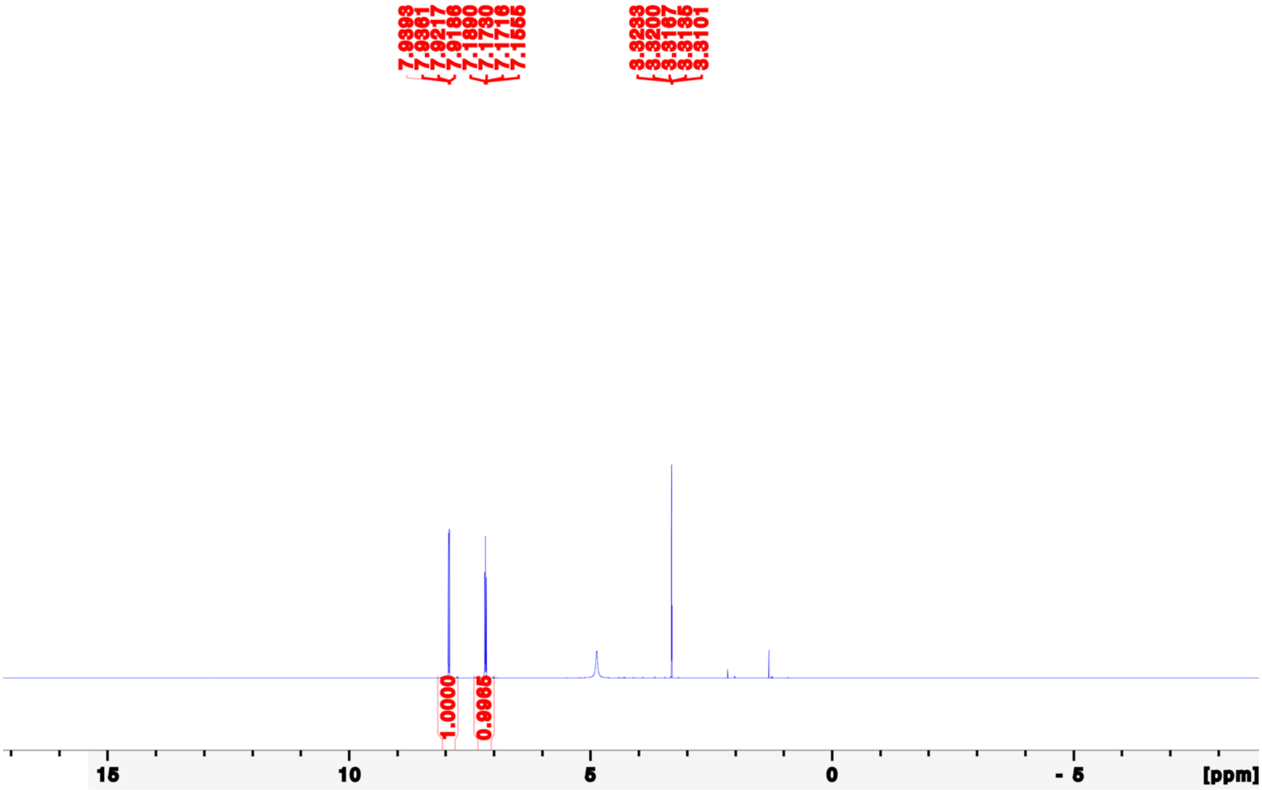

^{13}C, 126 MHz, CD_3OD

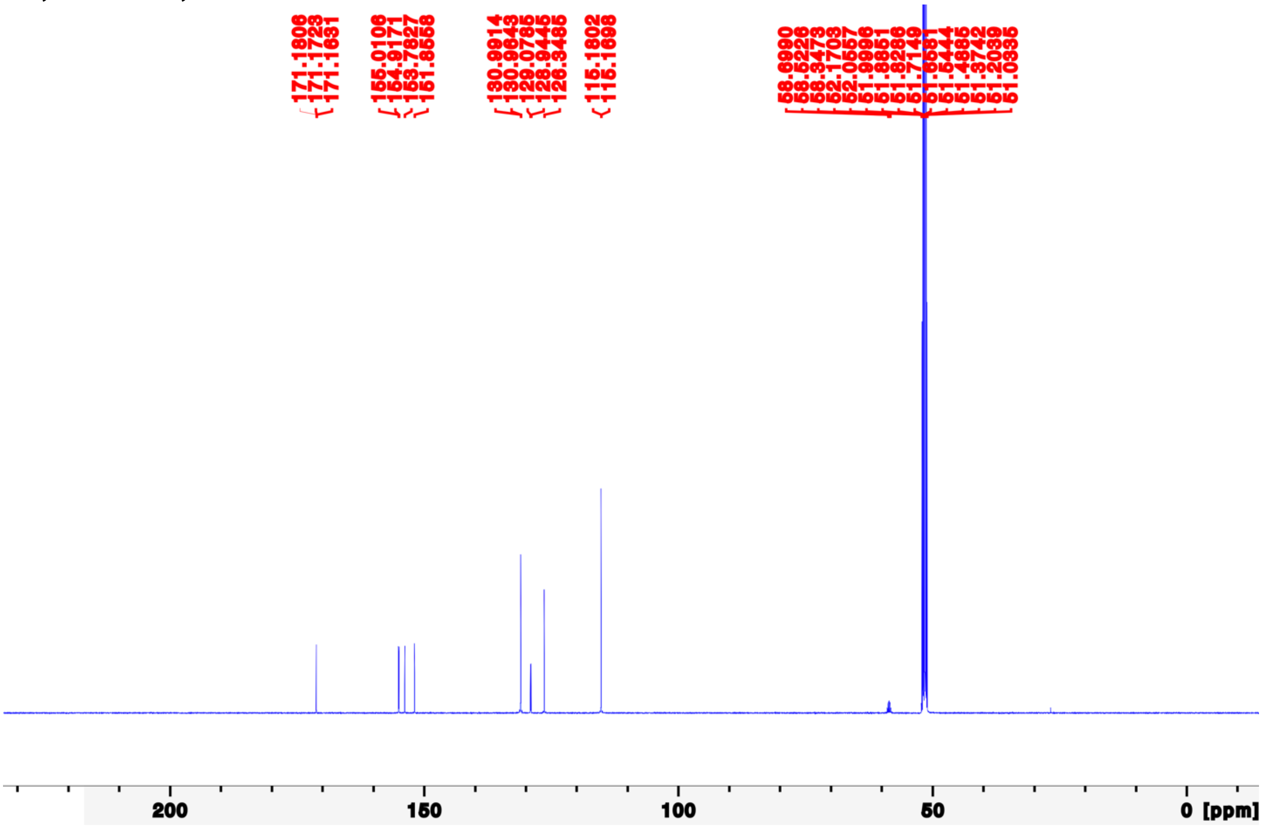

^{19}F, 471 MHz, CD_3OD

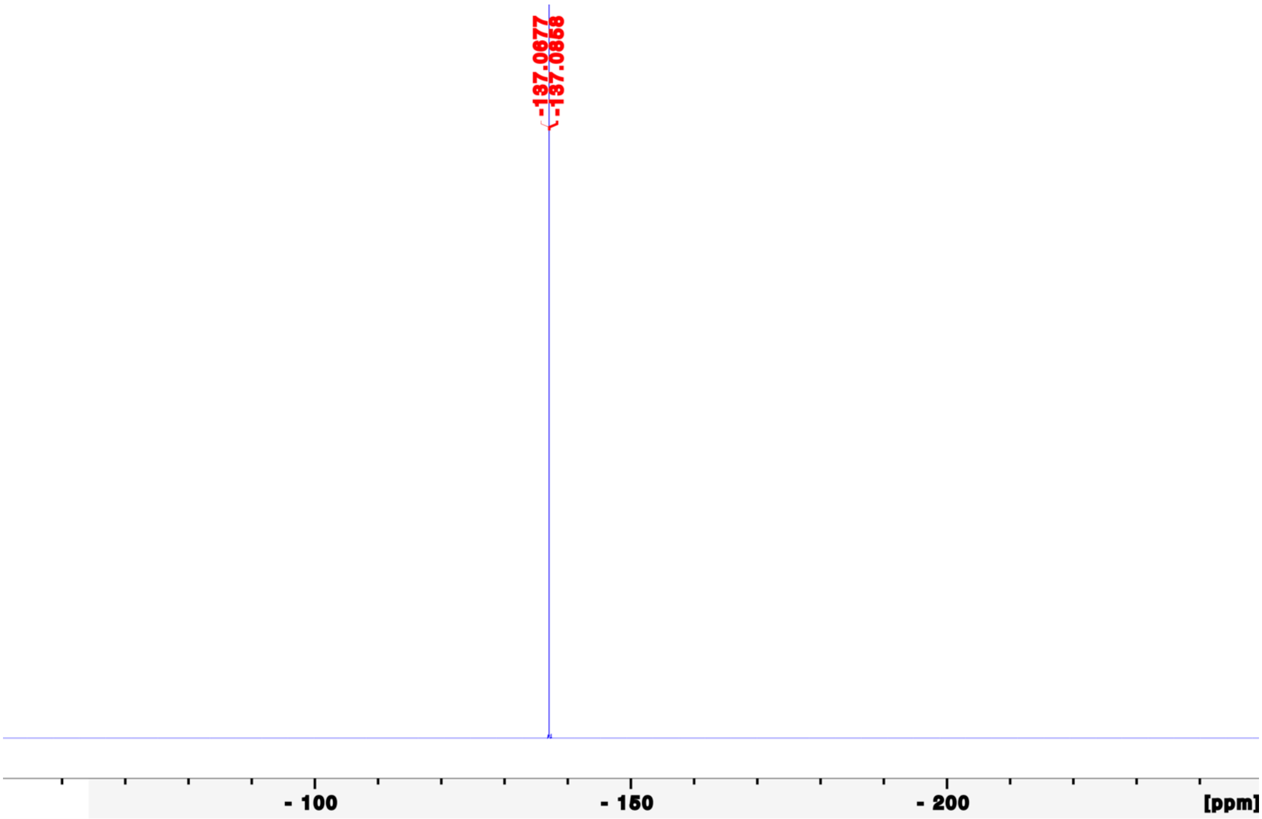

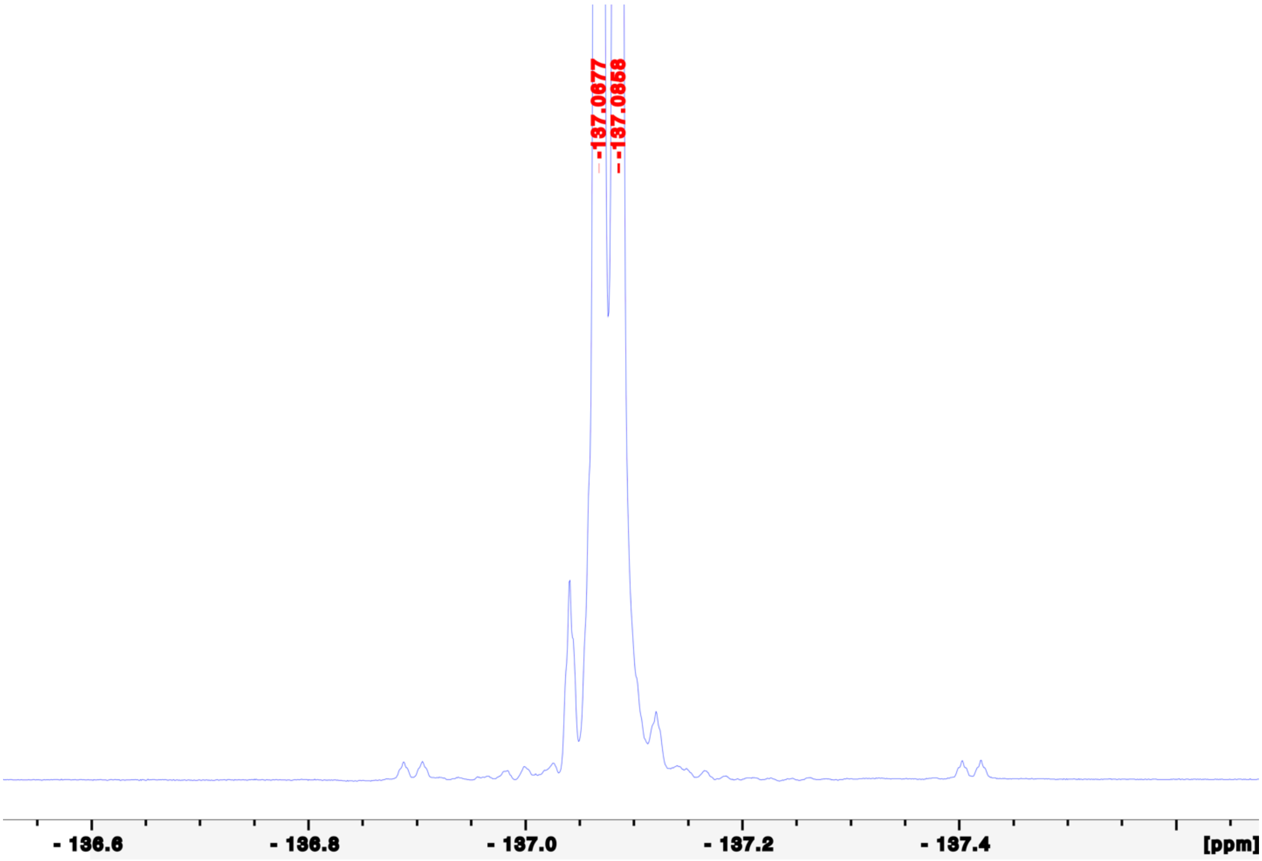

$^{19}F\{^1H\}$, 471 MHz, CD_3OD

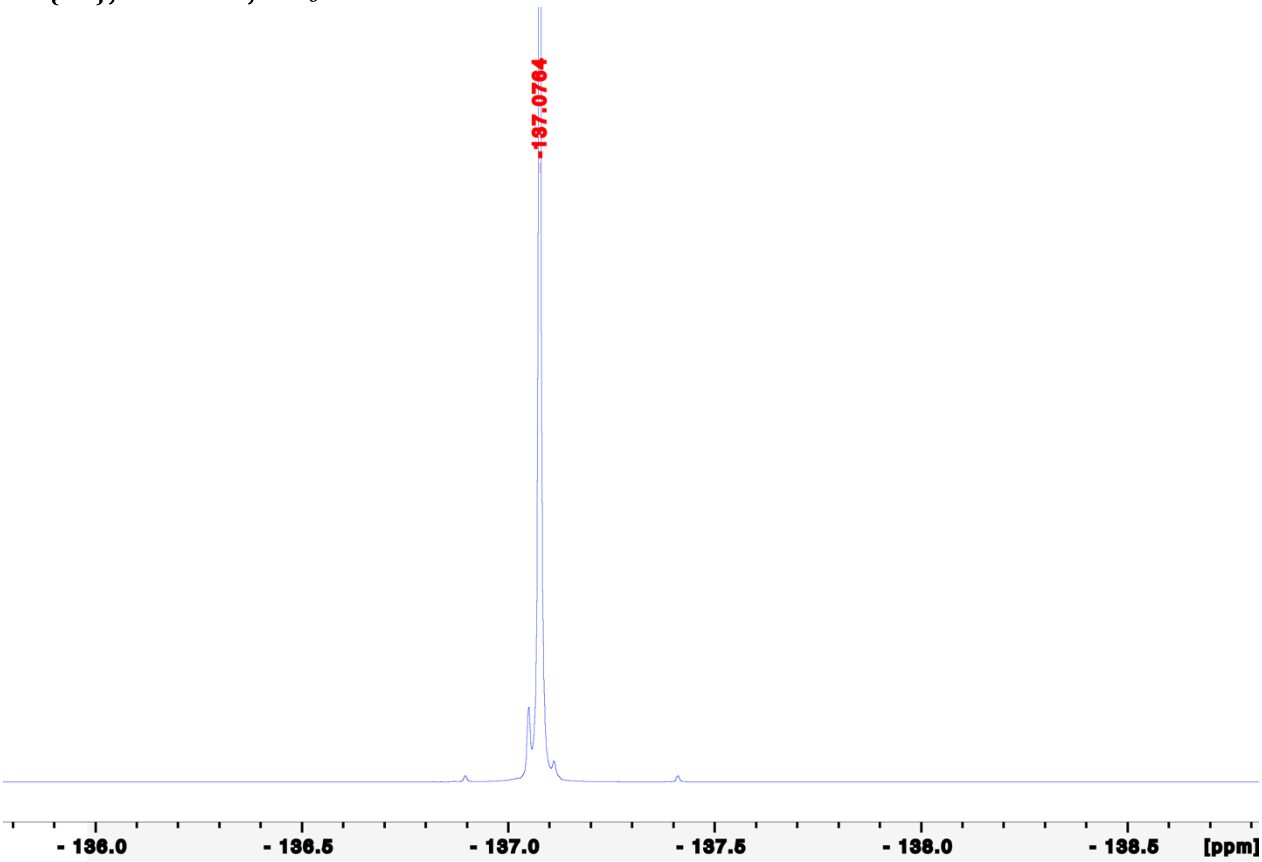

^2H, 77 MHz, CH_3OH

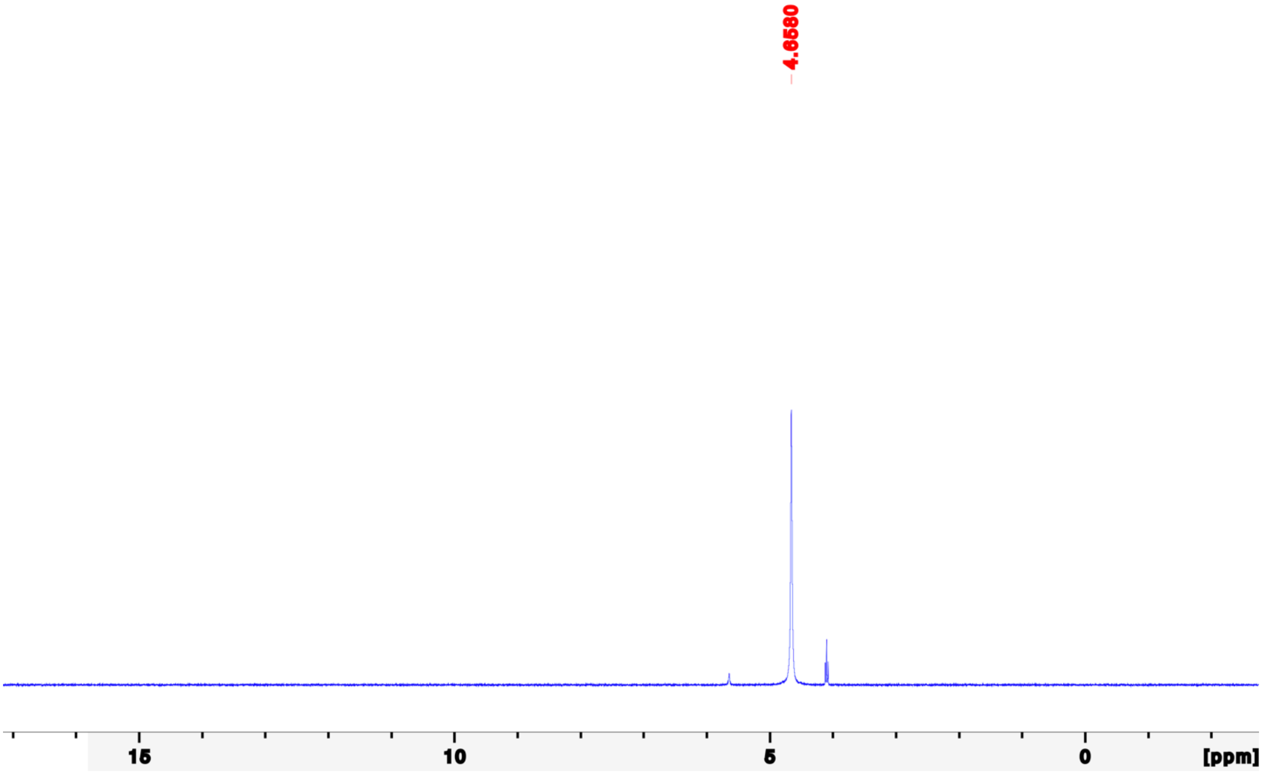

1,10-Difluoro-2,9-bis(methoxy-*d₃*) phenanthridin-6(5*H*)-one 'Molecule III'

^{1}H, 500 MHz, $(CD_3)_2SO$

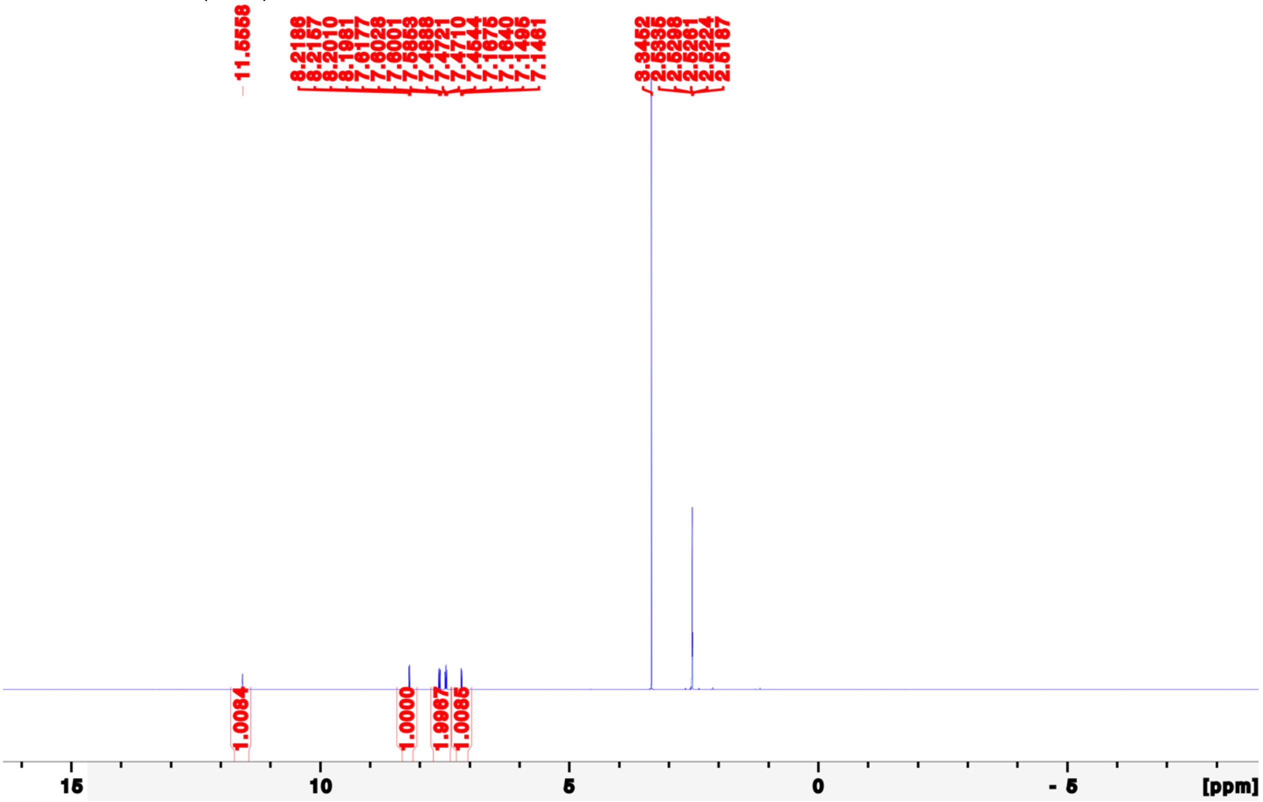

^{13}C, 126 MHz, $(CD_3)_2SO$

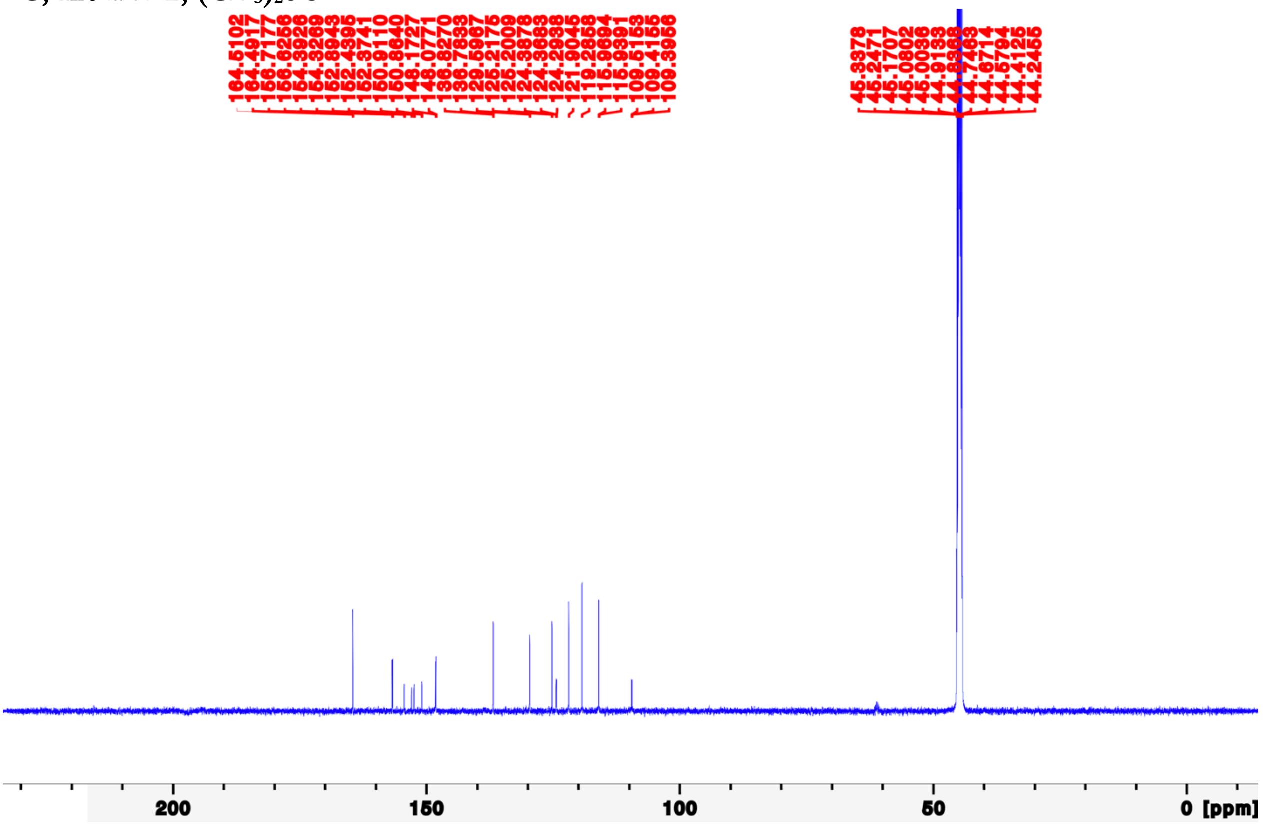

^{19}F, 471 MHz, $(CD_3)_2SO$

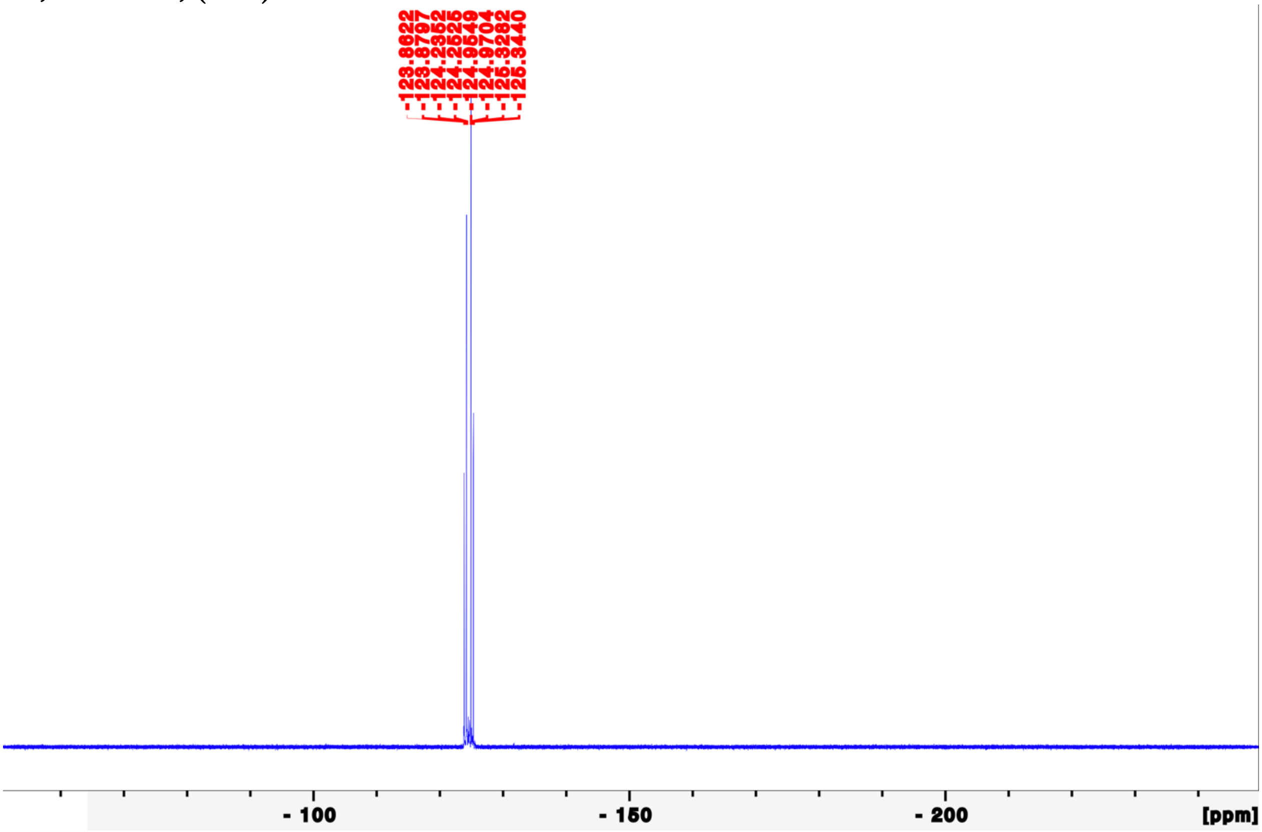

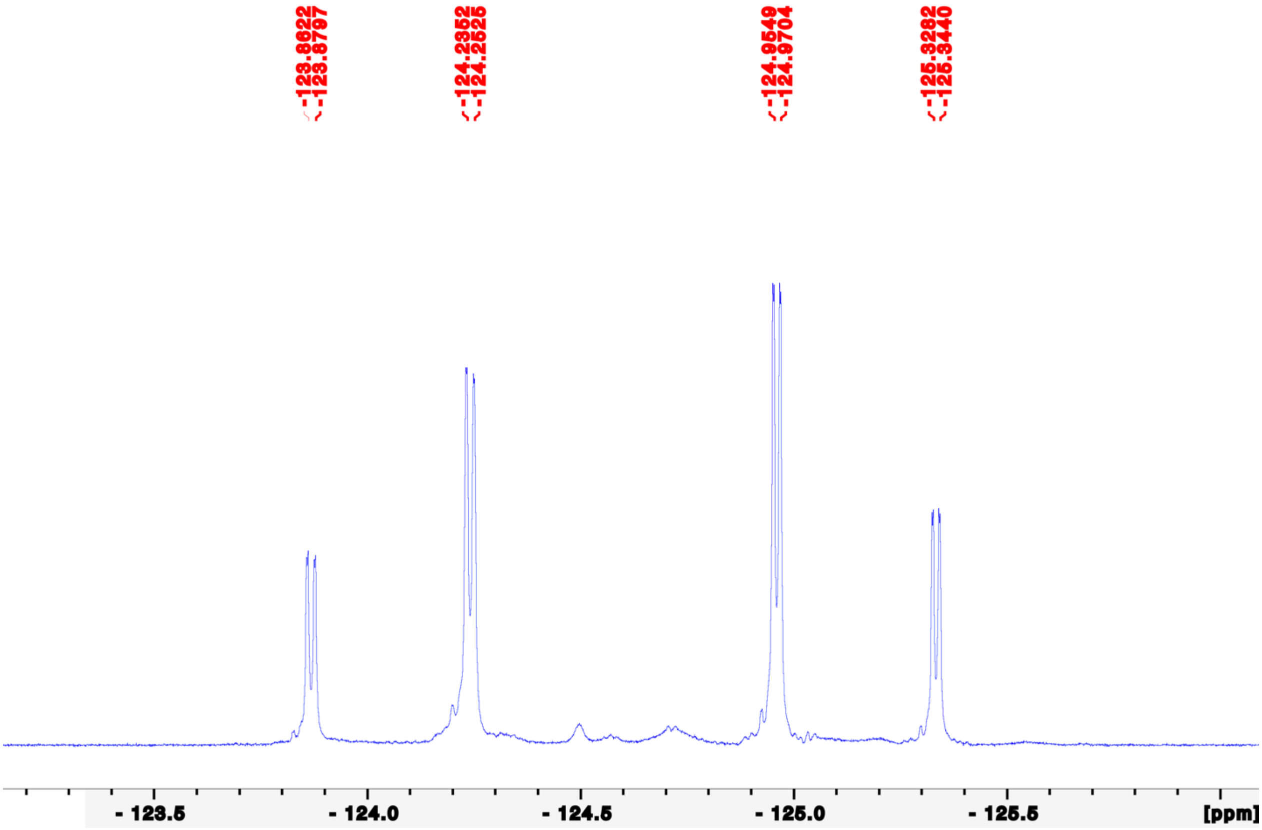

$^{19}F\ \{^1H\}$, 471 MHz, $(CD_3)_2SO$

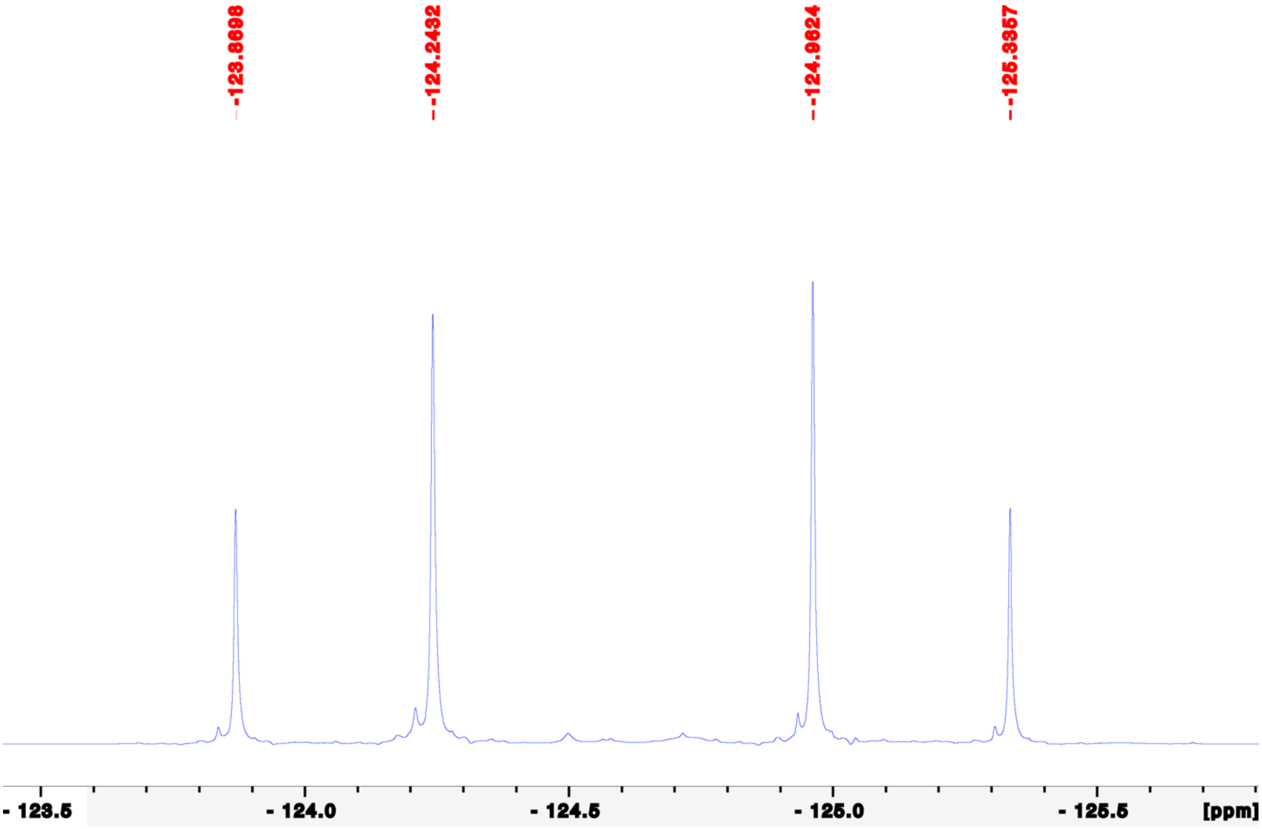

^2H, 77 MHz, $(CH_3)_2SO$

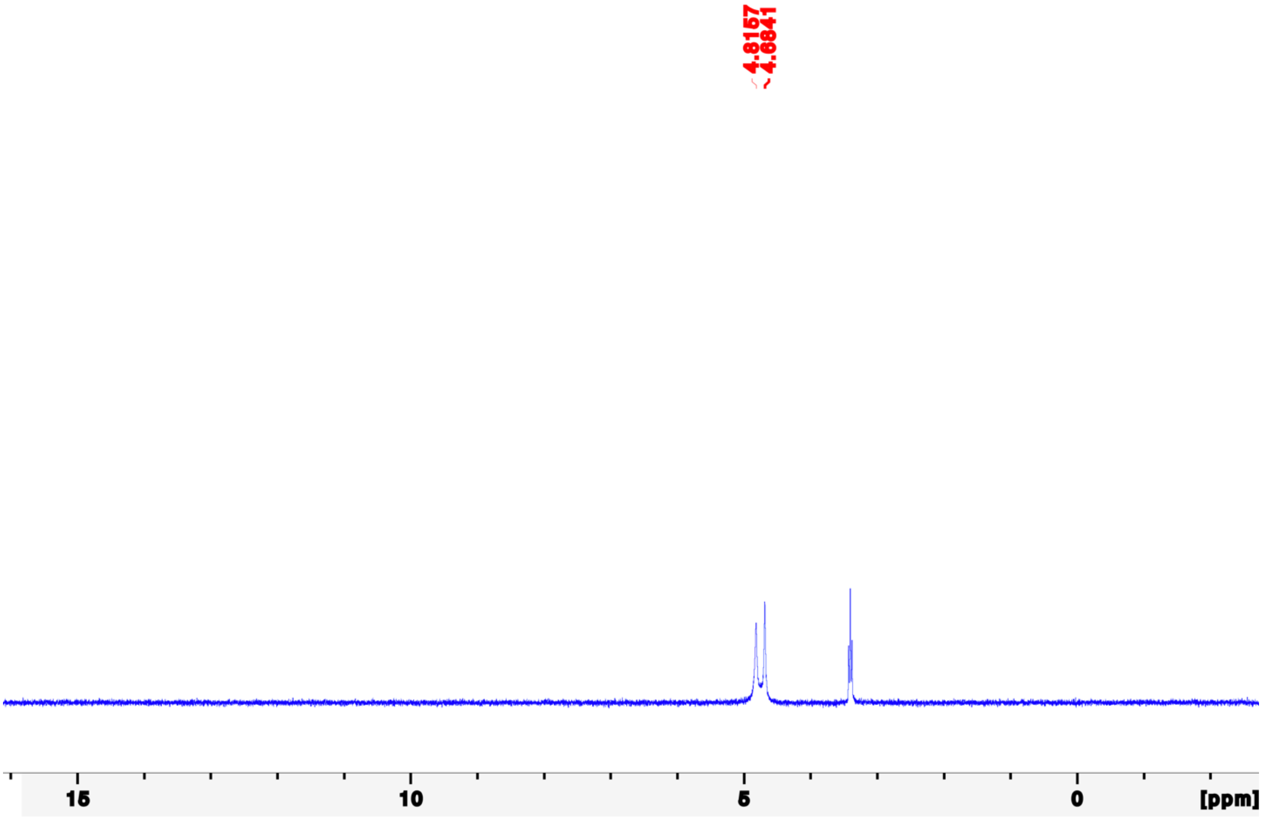

Supplement: Supplementary file 1 [file DataSheet1.pdf]
